# Edgetic perturbation signatures represent known and novel cancer biomarkers

**DOI:** 10.1038/s41598-020-61422-3

**Published:** 2020-03-09

**Authors:** Evans Kataka, Jan Zaucha, Goar Frishman, Andreas Ruepp, Dmitrij Frishman

**Affiliations:** 1 Department of Bioinformatics, Wissenschaftszentrum Weihenstephan, Technische Universität München, Maximus-von-Imhof-Forum 3, 85354 Freising, Germany; 2 Laboratory of Bioinformatics, RASA Research Center, St Petersburg State Polytechnic University, St Petersburg, 195251 Russia; 3 Institute of Experimental Genetics (IEG), Helmholtz Zentrum München-German Research Center for Environmental Health (GmbH), Ingolstädter Landstrasse 1, 85764 Neuherberg, Germany

**Keywords:** Data integration, Gene regulatory networks

## Abstract

Isoform switching is a recently characterized hallmark of cancer, and often translates to the loss or gain of domains mediating protein interactions and thus, the re-wiring of the interactome. Recent computational tools leverage domain-domain interaction data to resolve the condition-specific interaction networks from RNA-Seq data accounting for the domain content of the primary transcripts expressed. Here, we used The Cancer Genome Atlas RNA-Seq datasets to generate 642 patient-specific pairs of interactomes corresponding to both the tumor and the healthy tissues across 13 cancer types. The comparison of these interactomes provided a list of patient-specific edgetic perturbations of the interactomes associated with the cancerous state. We found that among the identified perturbations, select sets are robustly shared between patients at the multi-cancer, cancer-specific and cancer sub-type specific levels. Interestingly, the majority of the alterations do not directly involve significantly mutated genes, nevertheless, they strongly correlate with patient survival. The findings (available at EdgeExplorer: “http://webclu.bio.wzw.tum.de/EdgeExplorer”) are a new source of potential biomarkers for classifying cancer types and the proteins we identified are potential anti-cancer therapy targets.

## Introduction

Cancer involves the accumulation of somatic mutations^1^ and epigenetic modifications^2^, which drive the cells into the malignant state. Recurrent mutations implicated in tumorigenesis affect highly connected proteins within the protein interaction network^3,4^ and are enriched at the interaction interfaces^5,6^ and phosphorylation sites^7^ signifying their role in rewiring protein interactions^8^. For this reason, cancer has been described as the disease of the interactome^9^. Indeed, the network of protein-protein interactions (PPI) has repeatedly allowed for the extraction of molecular features predictive of various phenotypic traits relevant to cancer – the so-called disease biomarkers^10^. For example, Cui *et al*. have identified putative interaction-disrupting mutations occurring at the interfaces of protein complexes and demonstrated that their presence is prognostic of poor survival^11^. In another study, Li *et al*.^12^ developed the “OncoPPI” network of protein-protein interactions (PPIN) relevant to lung cancer, identifying biomarkers that can inform therapeutic decisions according to the drug sensitivity in certain conditions^12^. Nevertheless, the physical disruption of interaction sites by somatic mutations is only one mode of perturbing the interactome. Another relevant cellular process is regulating the expression (and thereby the local molecular concentration) of the interacting proteins^13^; this has been utilized in mining the network of protein-protein interactions to identify modules of differentially expressed genes serving as robust biomarkers indicative of breast cancer metastasis^14^ or stratifying patients from several breast cancer subtypes^15^. Furthermore, the phenomenon of “isoform switching”, i.e. altering the major splice variant of the gene that is favorably expressed within the cell, has been implicated in driving tumorigenesis and several such switches have been identified as biomarkers predictive of patient survival^16^. Interestingly, the majority of isoform switches observed across many cancer types could not be explained by somatic mutations in the same genomic locus suggesting that they usually arise through other complex molecular mechanisms^17^. In the case of multi-domain proteins, isoform switching can lead to the loss or gain of a domain responsible for mediating the interaction, thus perturbing the interactome. Recently developed computational tools leverage domain-domain interaction data in order to match transcriptomes to condition-specific interactomes, accounting for the major isoform of the protein that is expressed within the cell^18,19^. This allows comparing the healthy and cancer tissue interactomes from the same patient and identifying both the lost and the gained interactions (edgetic perturbations).

In this study, we analyzed all samples from The Cancer Genome Atlas for which both the healthy and cancer tissue RNA-Seq data was available, thus generating the first large-scale set of patient- and condition-specific interactomes along with the corresponding tumor-specific edgetic perturbations. Crucially, in contrast to recurrent somatic mutations that are typically present in only a small proportion of patients, many of the edgetic perturbations are consistently shared between the vast majority of patients across multiple cancer types, while other sets of perturbations are shared explicitly between patients in a given cancer type or sub-type. We show that in most cancer types the malignant tissue interactome is smaller than the interactome of the corresponding healthy tissue – the only significant exception to this trend was thyroid carcinoma (THCA). Interestingly, even though a considerable number of significantly mutated genes are cancer driver genes, they are not directly involved in a majority of the identified perturbations. Our results show high reproducibility of the perturbed co-occurring network biomarkers within patients of a cancer type (and subtype) and some shared network biomarkers across multiple cancer types. Furthermore, we found known (e.g. *TP73, NTRK1*, and *CDC25C*) and novel cancer biomarkers at the multi-cancer, cancer type and cancer subtype levels. These findings are a new source of robust biomarkers for detecting or classifying cancer types, may potentially point to new anti-cancer therapy targets and, owing to the extensive literature annotation we performed, they are also a comprehensive publicly available resource ready for experimental validation studies. We corroborate the relevance of the identified targets by demonstrating their strong correlation with overall patient survival and report the previously gathered insights on their role in tumorigenesis.

## Results

### Cancer PPINs are smaller than healthy PPINs in the majority of cancer types

**Figure 1:**
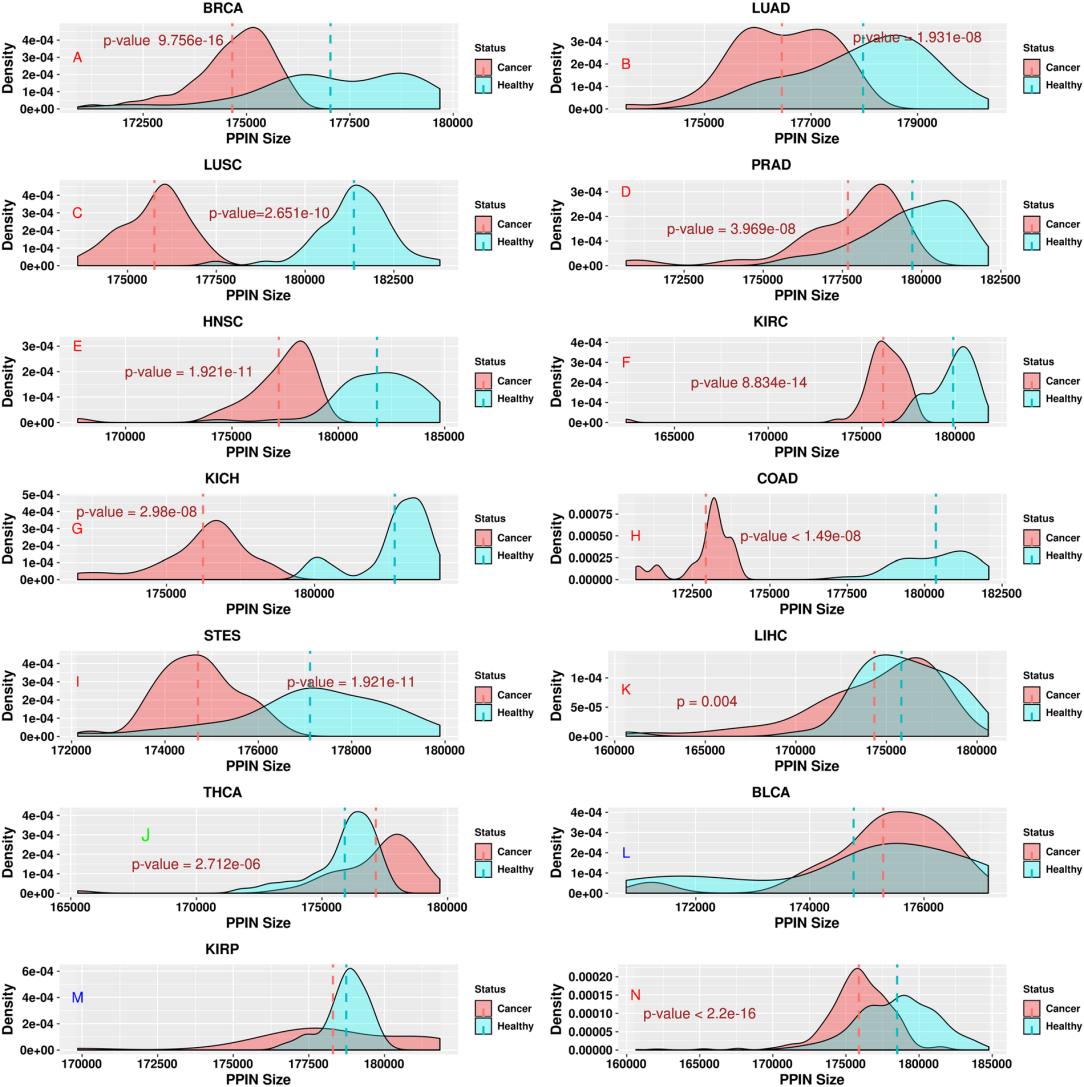
Healthy and cancer PPINs significantly differ in size in 11 out of 13 cancer types (p-value < 0.05). The density plots indicate the distribution of paired cancer and healthy PPIN sizes for individual cancer types (**A–M**) and across cancer types (**N**). The vertical dashed lines indicate the mean sizes of cancer PPINs (red) as compared to corresponding healthy PPINs (green). For BRCA, LUSC, PRAD, KIRP, KIRC, KICH, COAD, LIHC, HNSC and STES healthy PPINs were larger than the corresponding cancer PPINs but the difference was not significant in KIRP. For THCA and BLCA (green label), cancer PPINs were larger than the corresponding healthy PPINs, but the difference was not significant in BLCA.

We analyzed 642 paired cancer and healthy PPINs covering 13 cancer types derived from the global protein interaction network using patient-specific mRNA expression profiles. First, we used PPIXPress^18^ to construct cancer and healthy patient-specific PPINs. Next, using the Wilcoxon singed-rank test we tested the hypothesis that PPINs are disrupted during tumorigenesis by comparing the number of binary interactions observed in the healthy and the corresponding cancer PPIN for all patients with a given cancer type. Our results show that cancer PPINs are smaller than their corresponding healthy PPINs in 11 cancer types out of 13, and the difference is insignificant only in KIRP (Fig. 1A–K,M). In the remaining 2 cases (Fig. 1J,L) cancer PPINs are larger than the corresponding healthy PPINs, but the difference is only significant for THCA (p-value < 0.05). Similar results (apart from BLCA) were observed when using the randomized PPIN – Fig. S1.

While gene expression signatures have become the mainstay of cancer research, information about global transcriptome shifts between cancer and the corresponding healthy states is only beginning to emerge. In line with our findings, Danielsson *et al*.^20^ reported a reduction in the number of expressed genes in the course of malignant transformation. Distorted gene expression in cancer has been associated with genetic instability (e.g. chromosomal gains and losses^21^) and epigenetic control^21,22^. Anglani *et al*.^23^ reported that gene co-expression networks associated with pancreatic, cervical, gastric and non-small cell lung cancers exhibit losses of connectivity compared with healthy samples while colorectal cancer exhibits more gains in connectivity. We also find that edgetic losses prevail in STES (a type of gastric cancer) and in both LUSC and LUAD (non-small cell lung cancer subtypes). In contrast to Anglani *et al*.^23^ we found that colorectal cancer experienced more edgetic losses than gains, probably because our cohort consisted of only colon cancer (but not rectal cancer) patients. However, our results are in agreement with those of Cordero *et al*.^24^, where a significant reduction in colon tumor regulatory networks when compared with healthy samples was reported.

### Isoform switches and resultant domain changes between cancer and healthy states result in edgetic perturbations

**Figure 2:**
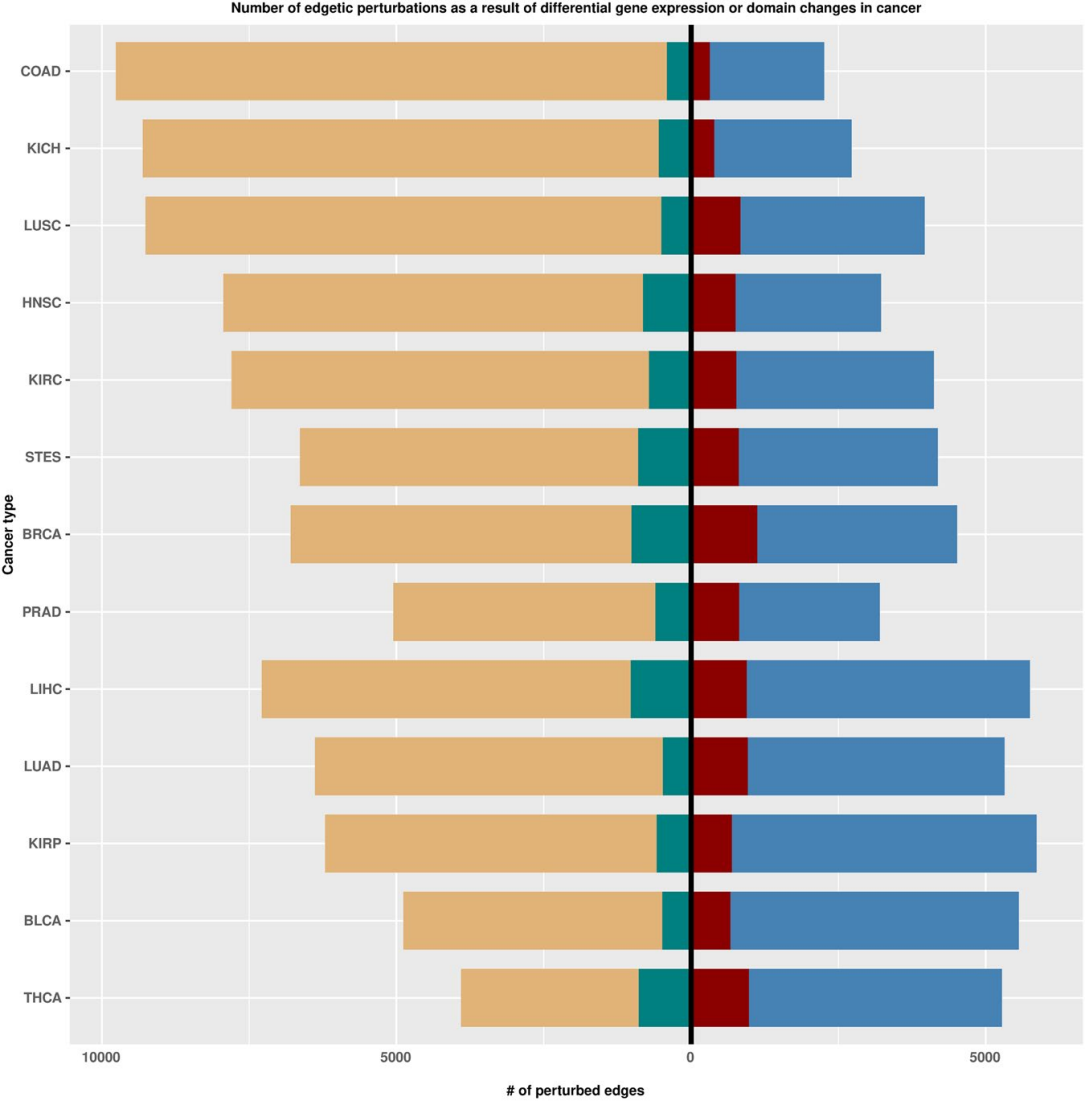
Bar plots indicating the number of edgetic perturbations obtained as a result of gene expression changes or domain changes that come about after isoform switches between cancer and healthy states. Sky blue: edgetic gains as a result of more genes being expressed in the cancer state, dark brown (left of zero intercept): edgetic gains as a result of isoform/domain changes (left of zero intercept). Light brown: edgetic losses as a result of the depletion of genes in the cancer state (right of zero intercept), light green: edgetic losses as a result of isoform/domain changes (right of zero intercept).

**Figure 3:**
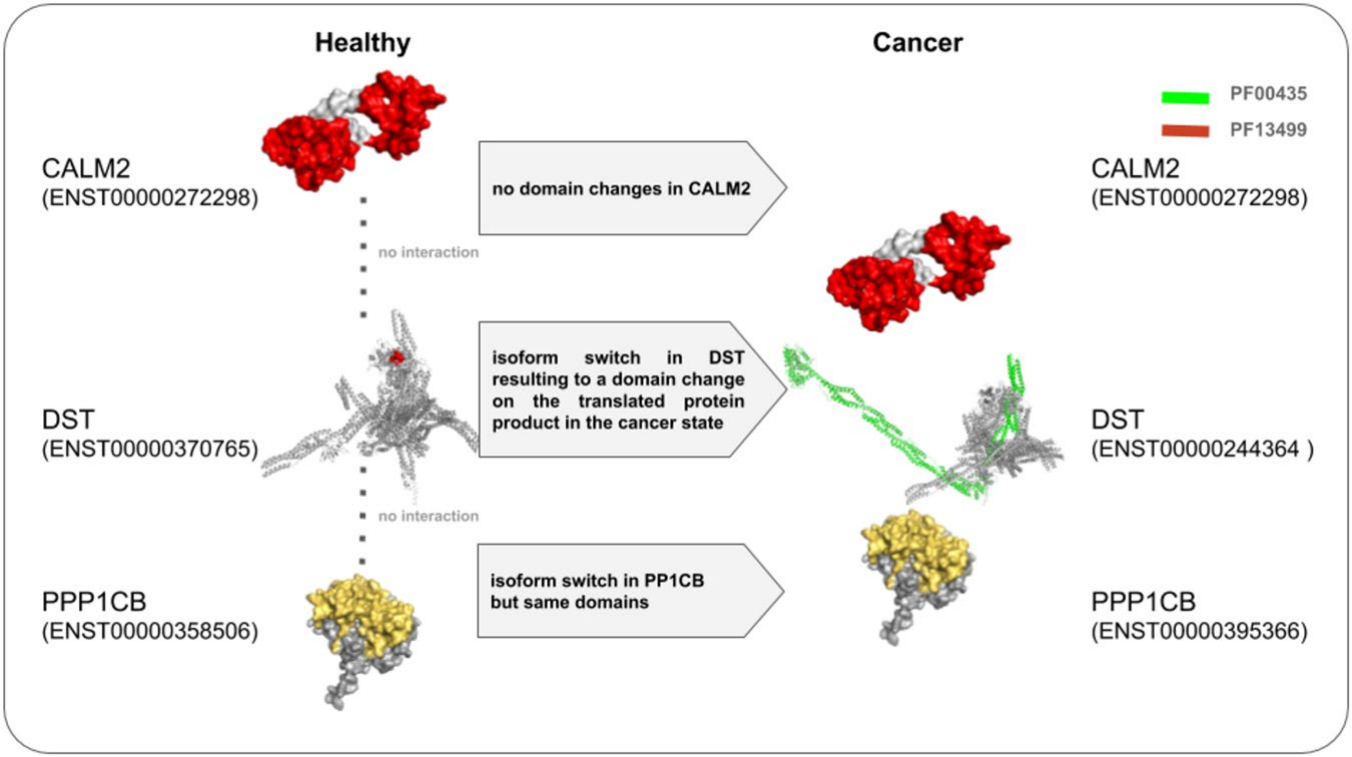
An example showing the consequences of domain changes between the cancer state and healthy state in patients diagnosed with BRCA. The protein structures of both (P0DP23) *CALM1* and (P62140) *PP1CB* were obtained from PDB while those of *DST* were modelled using the ensemble transcript sequences in SWISS-MODEL and visualized in PyMol. Following an isoform switch from ENST00000370765 (in healthy) to ENST00000244364 (in cancer), the protein Q03001 (*DST*) gained the domain PF13499. The consequence is the gain of interactions with the genes *PPP1CB* and *CALM1*.

The majority of the identified perturbations across the cancer types resulted from complete-protein-product losses or gains as a consequence of gene expression changes between the healthy and cancer states. Across all cancer types, the cancer state expressed slightly fewer genes than the healthy state apart from THCA, BLCA and KIRP (Dataset 1). Nevertheless, we obtained additional perturbations that were attributed to differential isoform expression (resulting in domain composition changes of the majorly expressed protein transcript) between cancer and healthy states – as exemplified in Figs. 2 and 3. Of the latter, most perturbations involved an isoform switch in either one of the interacting partners, however, we also identified cases of proteins where across patients of a given cancer type, isoform switches in both proteins were responsible for disrupting the interaction – Table S1. When using the randomized network derived from BiRewire, we were able to reobtain the prominent proteins involved in edgetic perturbations as a result of differential gene expression changes between the cancer and healthy state – Text S1 and Dataset 2. Our findings show that the transformation from the healthy to the cancer state results in (i) the loss or gain of gene expression, which alters the pool of proteins available within the interaction network, and (ii) differential isoform and domain expression, which further translates to the loss or gain of edges between the available proteins. Standard differential co-expression network analyses cannot detect such perturbations, thus making our approach appealing especially in the detection of the repertoire of proteins rewiring the interactome. Here, we corroborate a recent study by Climente-González *et al*.^25^, where the authors suggested that alternative splicing events promote tumor growth by, among other ways, remodelling the protein-protein interaction network.

### The identified edgetic perturbations are retained in the protein-abundance filtered PPIN

To test whether our approach yields reliable results, we additionally generated patient-specific PPINs using a smaller network with nodes constituted by highly abundant proteins (see Methods). The majority of the edgetic perturbations identified based on the global PPIN were retained within the reduced high-confidence set (see Table S2, Dataset 3 and in Dataset 4 for details). For instance, among the significant edgetic losses in BLCA samples, the protein abundance-filtered PPIN recovered one less edgetic perturbation involving the actin alpha skeletal muscle protein (*ACTA1*) and one of its interactors, neurabin-2 protein (*PPP1R9B*). The protein abundance database (PaxDb) does not report any abundance data for the neurabin-2 protein, and the protein is mainly undetected in the bladder samples from the human proteome map. Due to the modest correlations between mRNA and protein expression data^26,27^, it is still challenging to infer protein levels from transcriptome studies. Nevertheless, the majority of our results involve the highly-abundant proteins, which indicates that these interactions constitute the most relevant processes occurring within the cells and corroborates the reliability of the identified perturbations.

### Proteins involved in edgetic perturbations affect the overall patient survival and can serve as cancer type biomarkers

To find out whether changes in the expression of significantly mutated genes (SMGs) are the leading causes of the observed edgetic perturbations, we compared the proportions of perturbations involving SMGs versus those involving randomly generated proteins with a similar network degree. Surprisingly, across the majority of cancer types, more perturbations could be associated with the randomly selected genes rather than the SMGs (Table S3). For example, when looking at newly gained interactions connected to SMGs in comparison to randomly selected genes of similar degrees, only BLCA and LUSC showed significant enrichment. While the SMGs in our PPINs tended to be high-degree nodes, only a small number of their interactions exhibited frequent disruptions (in agreement with previous reports^28^), unlike the case for many other genes of a similar degree whose interactions were often perturbed. One possible explanation for this is that a majority of the randomly selected genes were house-keeping genes occupying more central positions in the PPINs^29^ and thus highly prone to rewiring as detailed by Kim *et al*.^30^. Also, this can mean that SMGs have subtle effects on the PPIN and affect the same interaction partner consistently across patients. Nevertheless, among the frequently perturbed edges across patients of a cancer type, we found multiple SMGs among the perturbed edges in all cancer types except in LIHC (Table S4a). With a rise in the interest for therapeutic targeting of cancer enabling proteins at the PPIN level^31^, our findings suggest that extending the range of target proteins beyond only the SMGs may augment the efficacy of anti-cancer treatments. To gain insight into the possible roles the proteins involved in edgetic perturbations may have in tumorigenesis, we used SurvExpress (except for KICH whose data is absent in the database) to analyze if the expression changes of these proteins could predict overall patient survival (OS) and distinguish between patients with longer and shorter lifespans following tumorigenesis. For each cancer type, we selected proteins connected by the significantly perturbed edges and randomly chose a similar number of proteins from the non-perturbed edges to predict overall patient survival. In all cancer types, we found that all the significantly perturbed edges harbor proteins that significantly affect patient survival (log-rank p-value < 0.05, Table S4 and Fig. S2) while the non-perturbed edges did not contain proteins that could predict the overall patient survival. To understand the roles these proteins play in KICH tumorigenesis, we performed text mining in PubMed using the protein identifiers plus the term cancer for each protein involved in significant edgetic perturbations^32^. The results for each individual cancer type are summarized in the Text S1, with the corresponding images available in Figs. S2 and S3. For the results of the survival analysis using the proteins obtained after network randomization, see Fig. S4.

### Proteins involved in cancer-specific edgetic gains and losses possess distinct functional roles

Based on the perturbation profiles associated with each cancer type, we identified two different edgetic events – those occurring in only one patient (patient-specific perturbations) and those occurring in at least 2 samples (cancer type perturbations). In the latter, perturbed edges present in only one cancer type are cancer-specific perturbations (Table S5) while those present in at least 2 cancer types are multi-cancer perturbations. A detailed summary of the results is available in Table S5. Overall, LIHC had the highest number of both cancer-specific edgetic gains and losses (6087), meaning that LIHC is more susceptible to cancer-specific perturbations (unique perturbations) than other cancer types. On the other hand, LUAD had the least number of both cancer-specific gains and losses (1218), suggesting that LUAD is least susceptible to cancer-specific perturbations, and is more likely to share most perturbations with other cancer types. These findings are in line with previously published results, which suggest that the liver has a large number of genes showing tissue specific expression^33,34^, while the lung has a low number of such genes^35^.

**Figure 4:**
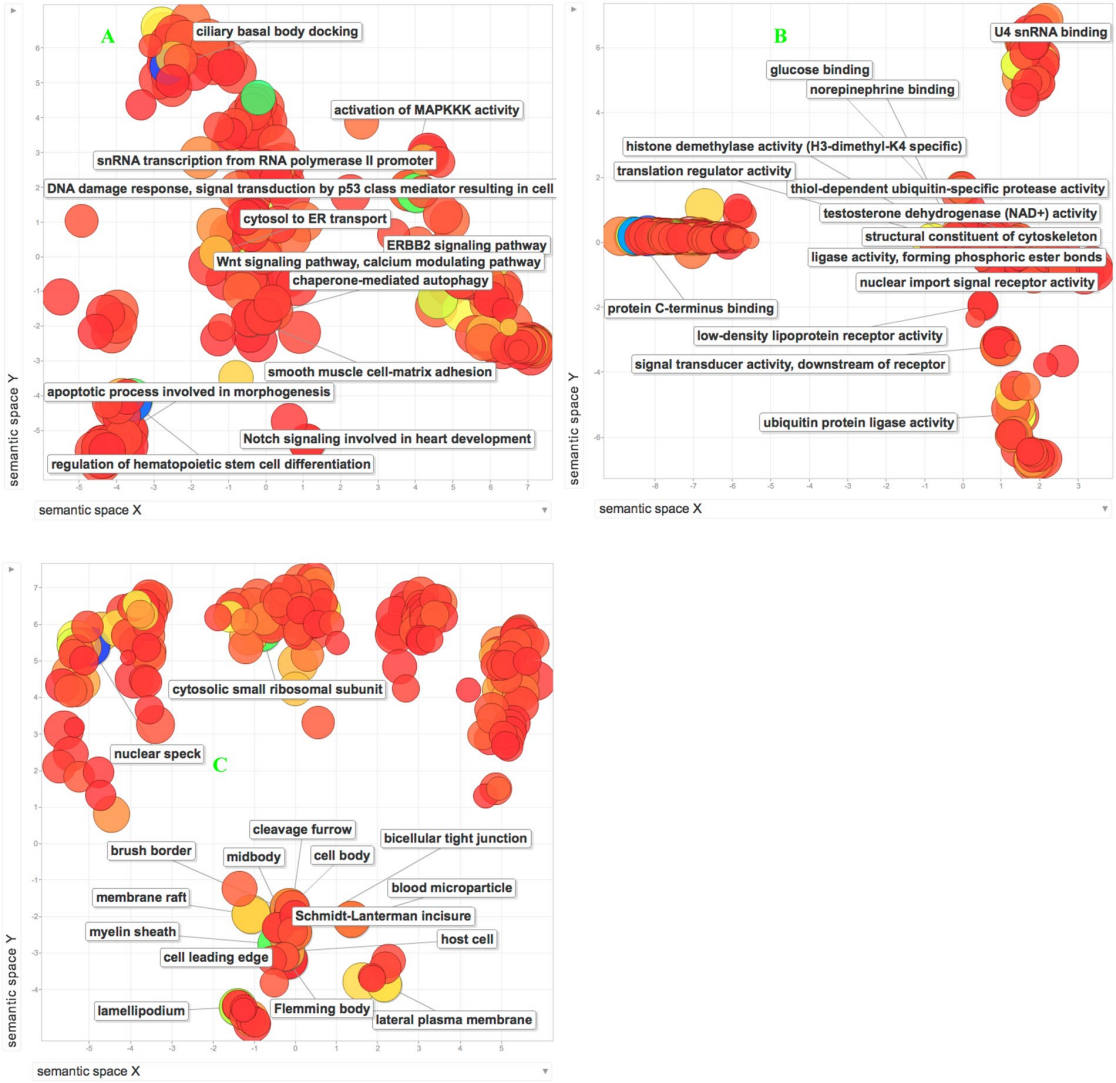
A two-dimensional scaling projection of the enriched Biological processes (**A**), Cellular Components (**B**) and Molecular functions (**C**) for proteins involved in cancer-specific edgetic gains after REVIGO pruning (dispensability value < 0.005). Dispensability of a term represents both the degrees of redundancy and enrichment. The lower the dispensability of a term, the least redundant and more significant a term is. The axes show the distribution of the GO terms based on their semantic similarities. The bubble color reflects the degree of significance (p-value) with blue color indicating a higher significance than the red color. The richly colored bubbles in the foreground represent GO terms with a dispensability value of <0.005. The bubble sizes indicate how often a GO term occurs, the bigger the size the more frequent the term is.

To explore the biological implications of edgetic perturbations we carried out a GO enrichment analysis using topGO and then employed REVIGO to group together the enriched GO terms. Among the proteins involved in edgetic gains, REVIGO summarized their enriched GO terms into 8 biological processes (BP), 14 cellular components (CC), and 51 molecular functions (MF) (dispensability value < 0.05 after REVIGO pruning, Fig. 4A–C). Of these enriched GO terms, 2 biological processes, 5 cellular components, and 8 molecular functions had a dispensability value of 0 (see details in Table S6). Our results support previous findings that suggest that lysosomal transport and viral processes mediate cell proliferation and apoptosis in cancer cells^36,37^ by targeting cellular components such as the focal adhesions or retromer complex^38,39^.

**Figure 5:**
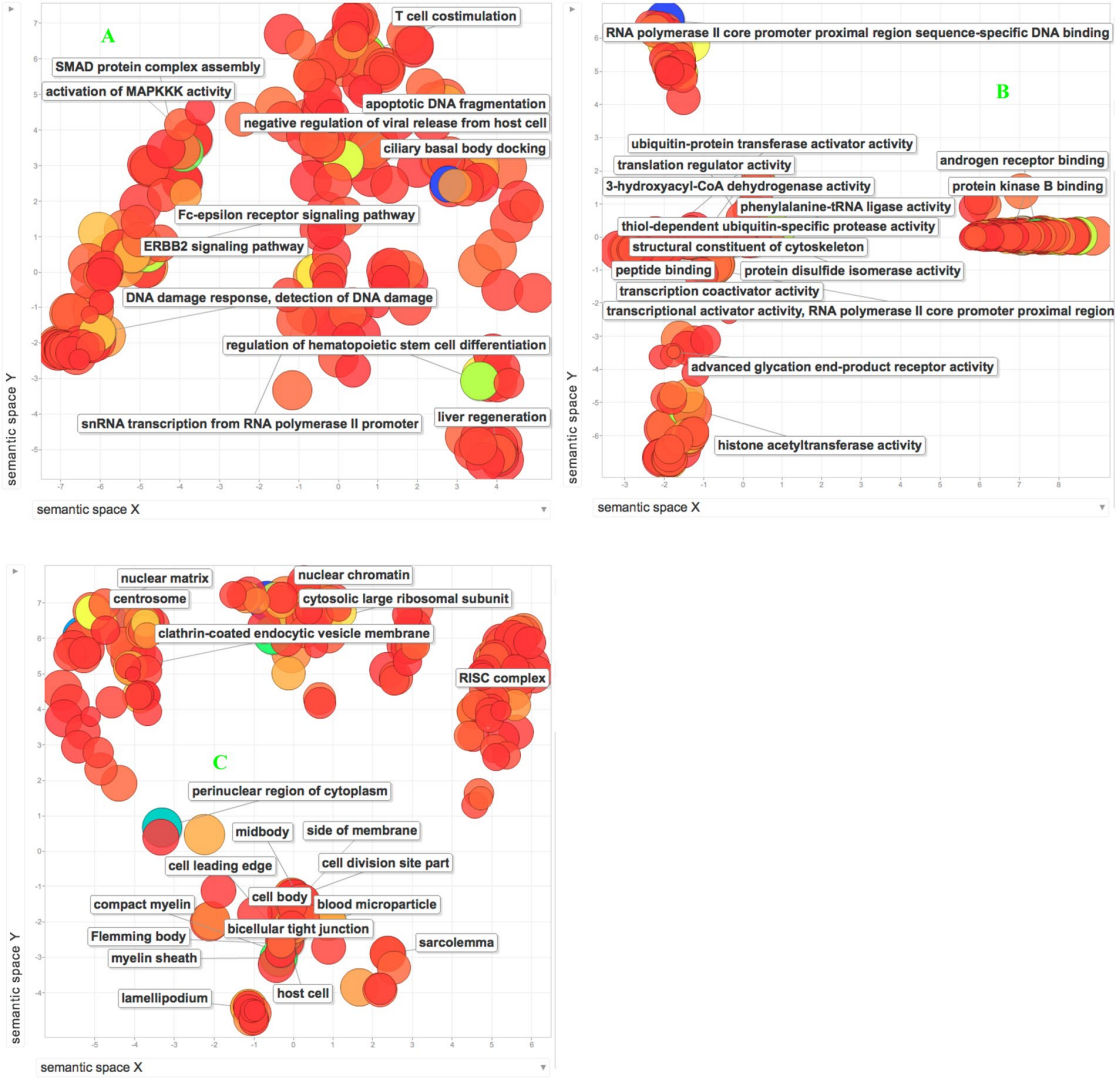
Two-dimensional scaling projections of the enriched Biological processes (**A**), Cellular Components (**B**) and Molecular functions (**C**) for proteins involved in cancer-specific edgetic losses after REVIGO pruning (dispensability value < 0.05). Dispensability of a term represents reduced redundancy and a high degree of enrichment. The lower the dispensability of a term, the least redundant and more significant a term is. The axes show the distribution of the GO terms based on their semantic similarities. The bubble color reflects the degree of significance (p-value) with blue color indicating a higher significance than the red color. The richly colored bubbles in the foreground represent GO terms with a dispensability value of < 0.005. The bubble sizes indicate how often a GO term occurs, the bigger the size the more frequent the term is.

For the proteins involved in edgetic losses, REVIGO clustered their enriched terms into 7 biological processes, 17 cellular components, and 51 molecular functions (dispensability value < 0.05 after REVIGO pruning, Fig. 5A–C). Of these, 3 biological processes, 3 cellular components, and 11 molecular functions (see details in Table S6) had a dispensability value of 0. These results complement previous work that indicates the importance of pathogens and transcription deregulation via the RISC complex during tumorigenesis^40,41^.

On the one hand, our results may suggest that proteins involved in edgetic gains may be recruited to upregulate cancer cell proliferation and put critical pathways under stress, as previously suggested^42^. On the other hand, edgetic losses appear to cause the deregulation of transcription activities as well as the distortion of epithelial cell polarity, an essential process in cancer cell transport membranes^43^.

### Hierarchical clustering of perturbed edges reveals cancer types sharing similar perturbation signatures

Cancer hallmarks often to cut across cancer types^44^, we thus sought to find out whether cancer types might also share perturbed network edges. To this end we merged all lost and gained edges to build multi-cancer loss and gain profiles, respectively (Table S7). The maximum number of cancer types sharing edgetic perturbations (either gains or losses) was 9 out of 13. We found 82 and 2178 gained and lost edges shared across 9 cancer types, respectively (Table S7), with the Q9BZD4 (*NUF2)* and P04629 (*NTRK1)* proteins associated with the largest number of perturbations (see Table S7 for details). The majority (98.78%) of edgetic gains involved 6 proteins, with the *NUF2* protein alone being a subject in 36.58% of the perturbations. Most edgetic losses (98.76%), on the other hand, involved a common set of 35 proteins, with the *NTRK1* protein being involved in 88.34% of the perturbations. Both of these proteins are known cancer drug targets and are now being considered as crucial molecules in the development of tumor-agnostic drugs to treat diverse cancer types^45^. Silencing of the *NUF2* protein has been shown to hinder tumor growth across cancer types^46,47^ while deregulation of the *NTRK1* protein has been successfully targeted by the drug Entrectinib^48^. Our results, therefore, suggest that the drug Entrectinib may be a choice in the treatment regimen of a diverse number of cancer types but may not be beneficial to patients diagnosed with STES, BLCA, LUSC (apart from ROS1-positive) and PRAD. A higher proportion of the multi-cancer edgetic losses compared to edgetic gains implies that cancer progression favors the loss of crucial protein interactions preventing the cell’s safeguards from inhibiting malignant proliferation. This phenomenon was also observed in the SMGs, most of them were involved in edgetic losses rather than in edgetic gains (see detailed results in Table S3e,g).

**Figure 6:**
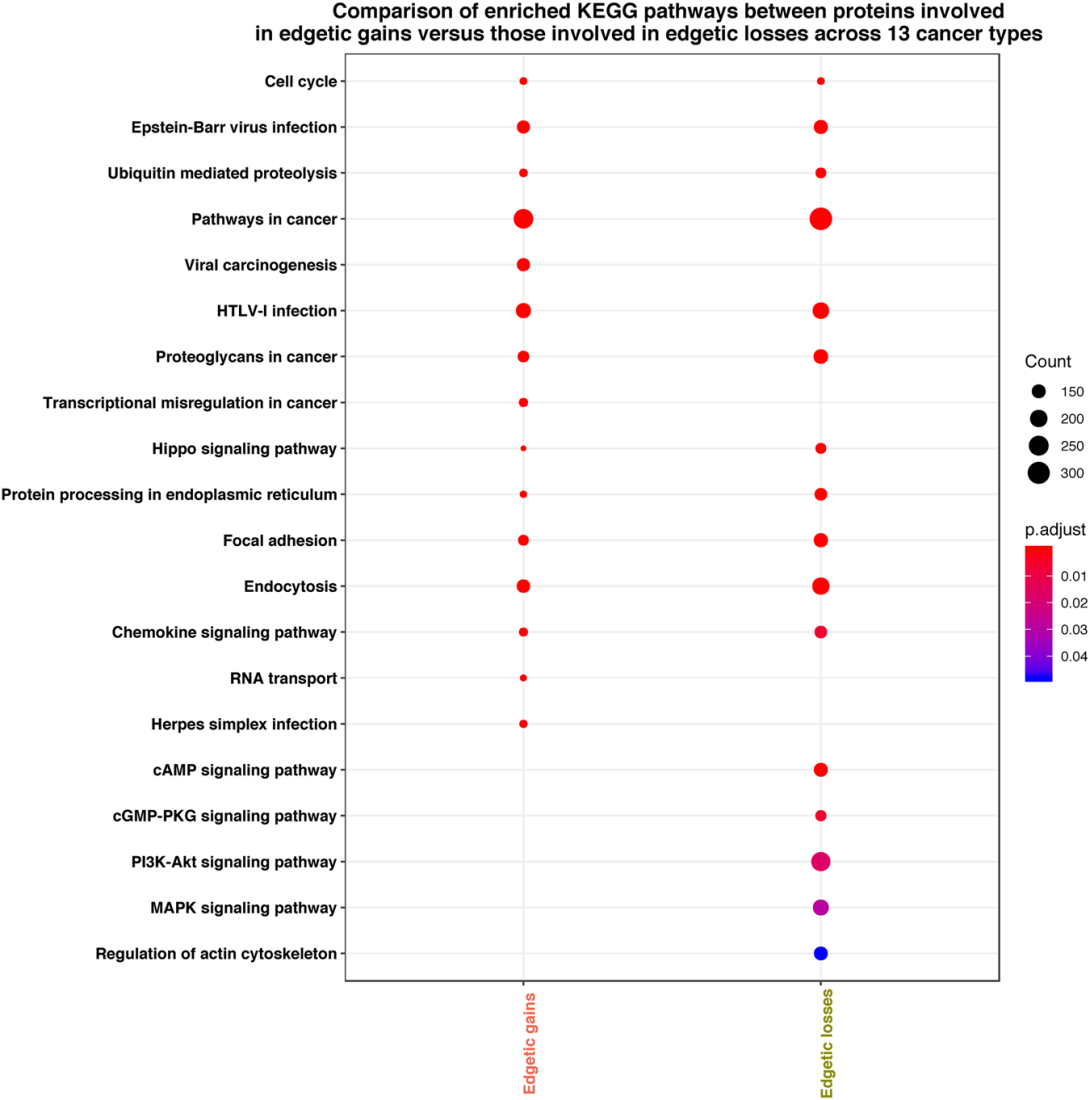
KEGG pathways differentially enriched between the proteins engaged in edgetic gains and those involved in edgetic losses. The dot colour reflects the degree of significance (p-value) with red colour indicating a higher significance than the blue colour. The dot sizes indicate how often a KEGG pathway term occurs. The bigger the size, the more frequent the term occurs.

Analysis of the significantly enriched KEGG pathways affected by the proteins involved in multi-cancer edgetic perturbations revealed known pathways^49,50^ deregulated across cancer types - Fig. 6 and Dataset 3. Additionally, we observed pathways that were unique only to the proteins involved in edgetic gains or in edgetic losses (Dataset 3).

**Figure 7:**
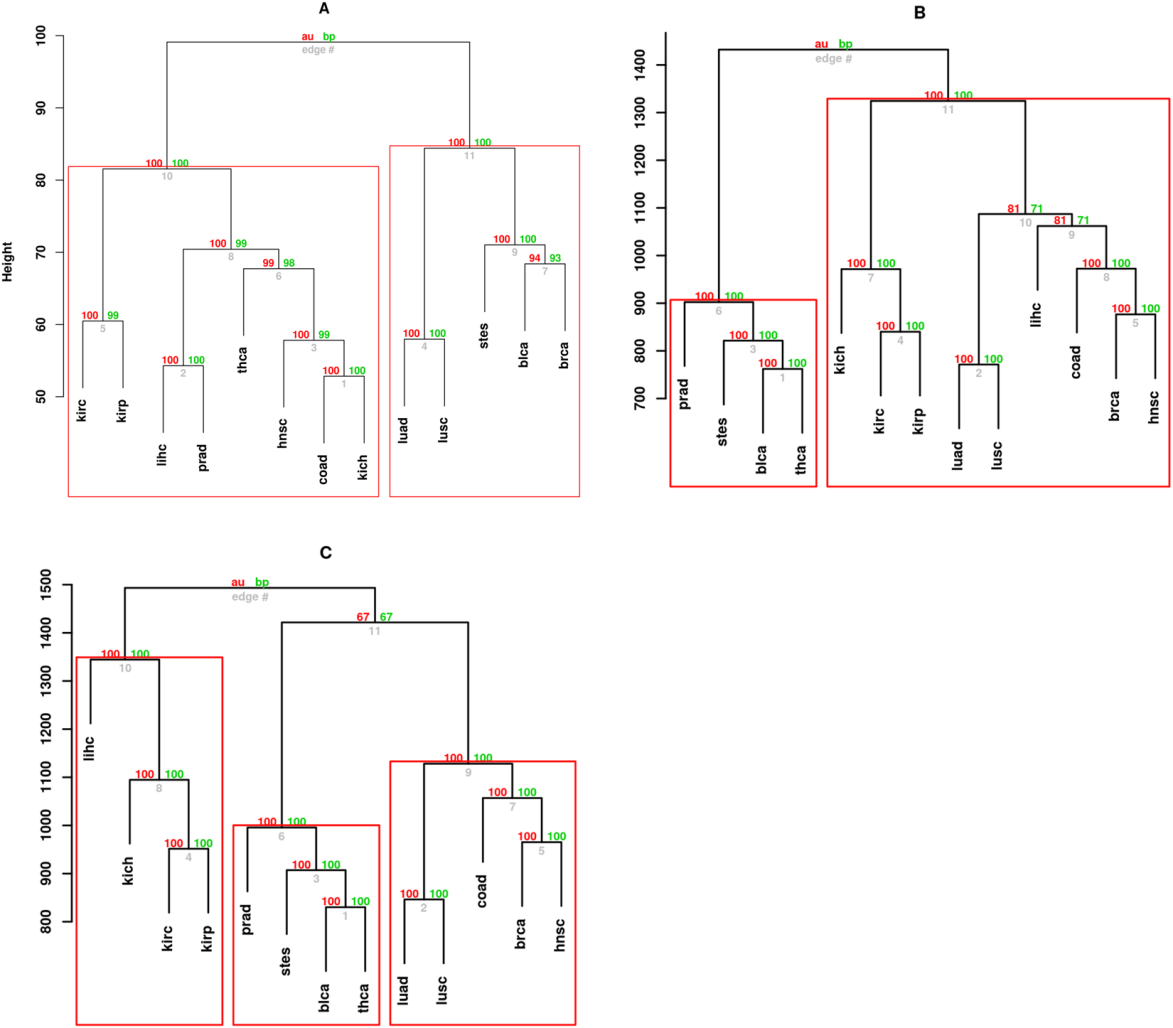
Cancer types share multiple perturbation patterns: Dendrograms based on edgetic gains (**A**), edgetic losses (**B**) and both edgetic gains and losses (**C**) across cancer types. Gained edges revealed 2 main clusters (**A**) with sub-clusters consisting of (i) BRCA, BLCA and STES, (ii) LUAD and LUSC, (iii) COAD and KICH, (iii) LIHC and PRAD, and (iv) KIRC and KIRP. Lost edges identified 2 main clusters (**B**) with additional sub-clusters consisting of (i) KICH, KIRP, and KIRP, (ii) LUAD and LUSC, (iii) COAD, HNSC and BRCA, (iv) STES, BLCA and THCA. Clustering of both edgetic gain and loss patterns revealed 3 main clusters (**C**) consisting of (i) LIHC, KICH, KIRC, KIRP, (ii) PRAD, STES, BLCA, THCA and (iii) LUAD, LUSC, COAD, BRCA and HNSC. The Approximately unbiased AU (green) and Bootstrap probability BP (red) scores indicate the likelihood of observing the obtained clusters. The clusters within the red rectangles with AU scores of >99% were observed after multiscale bootstrap (n = 10000). The edge # below the AU and BP values gives the edge count within the tree. The height indicates the similarity or dissimilarity between any two observations: the lower the height of the fusion between two observations, the more similar they are.

Even though intra-tumor heterogeneity offers crucial data during therapeutic decision making^51,52^, biomarkers cutting across multiple cancer types are invaluable in the clinical research set up as they shed light on pathways shared across cancer patients and inform on inter-tumor heterogeneity^53^. Because the edgetic gains and losses are responsible for affecting different molecular pathways, we considered them separately in clustering cancer types based on the perturbations observed. We performed this analysis three times (for edgetic gains/losses separately and considering all data together) under the assumption that the majority of gains or losses may have some common underlying cause (for example, molecular pathways), which may persist across multiple cancer types. Hierarchical clustering of the perturbation patterns using the R package Pvclust identified high confidence (p-value < 0.05) cancer clusters based on shared perturbation signatures (Fig. 7A–C). Using the random forest algorithm (see Methods), we found sets of edgetic perturbation patterns important in grouping cancer types into the identified clusters (Fig. 7). A comprehensive summary of these results can be found in the Text S1 and Fig. S5. In brief, our findings are in agreement with Yuan *et al*.^54^, who indicated that pan-cancer analyses reveal additional biomarkers that may be masked when searching for biomarkers in single tumor type studies.

### Cancer subtypes exhibit unique edgetic perturbation patterns

Among the cancer subtypes, we also searched for subtype-specific disruptions (Table S4b). We found that subtypes differed in their edgetic perturbations and that most of the proteins involved in these network disruptions might be responsible for the observed subtype phenotypes. For example, network edgetic disturbances involving the ribonucleoprotein IMP3 (*IGF2BP3)*, which frequently occurred in ER+, PR+, HER− subtypes, and those involving the m-phase inducer phosphatase 3 protein (*CDC25C*), often observed in ER−, PR−, HER+ subtypes, revealed the mutual exclusivity nature of BRCA subtypes. Also, some of the proteins whose edges were specifically perturbed within patients grouped in a particular cancer subtype may be novel subtype-specific biomarkers. For example, the protein cytochrome P450 1A1 (*CYP1A1)* is a probable biomarker in Classical LUSC subtypes, neuron navigator 2 protein (*NAV2)* in MSS STES subtypes and the apin protein (*ODAM)* in THCA BRAF-like subtypes. Furthermore, we found interacting proteins whose connections were differentially perturbed across cancer subtypes. For instance, while PR−/ER− BRCA and KIRP Type1 subtypes shared nearly all (71/73) edgetic gain perturbations involving the gene *IGF2BP3*, KIRP Type 1 also had two other edgetic perturbations affecting the *IGF2BP3* gene (*IGF2BP3 -KRT17* and *IGF2BP3 -SYT17*) suggesting that these proteins may have a probable role in the differential mechanisms between BRCA and KIRP tumorigenesis. Besides, we discovered biomarkers shared by several subtypes, for example, the core components of the nucleosome (*HIST1H2AB, HIST2H3A* and *HIST1H3A*) in Secretory and Classical LUSC, PRAD SPOP and BRCA HER+ subtypes. Somatic mutations in these histone proteins have previously been linked to cancer, thus suggesting the relevance of these molecules as candidate cancer subtype-specific biomarkers^55–57^.

### Proteins participating in significant edgetic perturbations are implicated across all cancer stages

For a mutated gene to be tumorigenic (*i.e*. to be a driver gene), it must accumulate mutations throughout the life of the cancer cell^58,59^. Consequently, cancer driver genes are implicated from the onset of cancer and progressively increase the survival of the cancer cell as the disease progresses. We hypothesised that edgetic perturbations that harbour essential biomarkers may play an important role in tumorigenesis and cut across all cancer stages. To determine if this phenomenon applied to edgetic perturbations, we searched for the stage distribution of the patients that had significantly perturbed edges in their PPINs. In all cancer types, the significantly perturbed edges were observed across all stages albeit in varying proportions, indicating their probable role from cancer onset and in progression (Table S8). Our results mirror those from Li^60^ who pointed out that essential cancer biomarkers are active in the entire life of a cancer cell.

### The EdgeExplorer website

The EdgeExplorer portal (http://webclu.bio.wzw.tum.de/EdgeExplorer) provides annotations for all the cancer-type specific proteins involved in edgetic perturbations (a total of 539 proteins). We annotated each protein by performing an exhaustive literature search for relevant experimental evidence linking it to the specific cancer type; if hits related to that cancer type were not found, we broadened the search to include other cancer types – for more details on how the search was performed, please refer to the Text S1. The main advantage of the web portal is that it allows for easy browsing of the results and searching for information on specific proteins. Moreover, it provides the functionality to download all of the annotated data.

## Discussion

Identification of cellular interconnections perturbed by diseases has long been recognized as a promising avenue towards elucidating reliable biomarkers^61^. Over the recent years this general idea was being actively put into practice by using molecular networks to study the differences between healthy and diseased states in cancer^12,62,63^. Here, we derived 642 patient-specific PPINs from patient-specific paired healthy and cancer mRNA expression profiles and identified candidate biomarkers significantly involved in distorting PPINs during tumorigenesis. In doing so we considered shared patient edgetic perturbation profiles across tumors, within a cancer type and further distinguished edgetic perturbation signatures between cancer subtypes. Our approach utilizes the publicly available data of paired cancer and healthy gene expression profiles from 13 cancer types and combines them with previously reported cancer-specific significantly mutated genes, and binary protein interaction data to identify proteins driving significant edgetic perturbations in cancer networks. For the first time, we show that using multiple patient-specific PPINs derived from the corresponding mRNA expression profiles of healthy and cancer patient samples is a novel way of identifying patient-, cancer-type and subtype as well as multi-cancer edges susceptible to perturbation during tumorigenesis. Furthermore, we were able to reproduce similar perturbed edges for each cancer type when using a smaller protein abundance-filtered PPIN (Dataset 3), validating our approach. We demonstrate that perturbed edges harbor known and novel cancer biomarkers and that they also capture previously reported cancer hallmarks^44,64^. While the differential expression of genes between the cancer and healthy state dictates the availability of proteins that interact with each other, we also show that alternative splicing events causing protein domain composition changes in the cancer state have effects on the protein-protein interaction network. A gain of an interacting domain may result in the gain of a new interaction while the loss of an interacting domain may bring about edgetic losses in the cancer PPIN - Fig. 3.

We found that the majority of perturbations were not attributed to SMGs either directly as first neighbors or indirectly as second neighbors within the PPIN - there were only several cancer types that did not follow this trend (Table S4b). One such exception was LIHC, and indeed the interactions disrupting mutations of SMGs in this cancer type have been recently reported to strongly affect survival, which indicates that our results are in agreement with previous findings from Cui *et al*.^11^. While our study cannot model the effects of mutations on the PPIN as undertaken by Cui *et al*., our results suggest that any mutations that are prevalent in the domains do promote edgetic perturbations and consequently tumorigenesis.

We also found that cancers exhibit either a high proportion of edgetic losses or a high proportion of edgetic gains. We speculate that this may be a downstream effect of a deregulation of the components of the spliceosome resulting in a systematic truncation or elongation of transcripts during pre-mRNA processing. However, most cancer types (9) showed more edgetic losses than edgetic gains, resulting in a reduction in the size of the cancer PPIN when compared to the corresponding healthy PPIN (Fig. 1). We also found multiple biomarkers already validated at multi-cancer, cancer type and subtype levels. When considering proteins driving significantly perturbed edges and using SurvExpress, our study confirmed most of the proteins as being biomarkers predictive of survival while those that did not show any perturbation were not prognostic in cancer. Moreover, at the multi-cancer level, known cancer drivers such as *CDC45* and *NUF2* were identified to be involved in edgetic gains while *NTRK1*, *PRPH* and *MYOC* were determined to be involved in edgetic losses and may serve as targets for widely applicable therapeutic interventions. For instance, *Liu et al*. showed that knockdown of *NUF2* may inhibit proliferation of carcinomas and may be a potential target for therapy in cancer^46^. Furthermore, our clustering analysis of the cancer perturbation profiles revealed novel relationships between cancer types. We found that KICH, KIRP, and KIRP, LUAD and LUSC, COAD, HNSC and BRCA, as well as THCA, BLCA, and STES shared a more significant proportion of lost edges. Also, BRCA, BLCA and STES, LUAD and LUSC shared a higher portion of gained edges. Targeting of the proteins shared and perturbed in these cancer types for clinical use could benefit patients diagnosed with these cancer types. For example, developing a therapy to target *UCHL1*, the protein most rewired across kidney cancers, would be an economical way of treating all kidney cancers by targeting the same molecule^65^.

At the cancer type level, some of the perturbations we identified, such as *IGF2BP3* and *DKK1-MDFI*, have already been suggested to be KIRP biomarkers. Our analysis supports the roles of these molecules as KIRP biomarkers, as they were among proteins significantly perturbed in KIRP and showed prognostic value when their expression changes were analyzed for predicting overall patient survival. We also uncovered known biomarkers for specific cancer types not yet directly linked to other cancer types. For example, *TRIM15* is a tumor suppressor in colon cancers^66^, however, to our knowledge no study has linked *TRIM15* to KICH tumorigenesis. We found *TRIM15* perturbations among the proteins involved in edgetic losses in KICH. Our study, therefore, suggests that *TRIM15* could also be an informative KICH biomarker. We also found multiple cancer-specific edgetic perturbation biomarkers such as the *SLC25A21* distortion in LUAD. Most importantly in KICH, our study is also able find perturbations of Bcl2 family proteins which are targeted by the only clinically approved drug (Venetoclax) targeting a protein-protein interaction^67^.

While previous studies such as Li *et al*.^12^ found biomarkers at the cancer network level (lung cancer), our study expands on this work to obtain cancer subtype-specific markers at the network level. Using our methodology, we identified probable subtype-specific biomarkers, including *PARVG* and *XPO4* in PR+ BRCA, *MYL1* in PRAD, *KHDRBS1-DLG2* edgetic perturbation in HNSC, and *KLF8* in STES. We also observed several cancer subtypes sharing perturbed proteins pointing to probable shared oncogenic patterns. Our findings, therefore, suggest that these subtypes could be targeted by similar therapies. Functional and pathway enrichment analysis further revealed that proteins driving edgetic perturbations are consistent with the observed cancer phenotype, that is, we obtained known canonical oncogenic KEGG pathways involved in viral carcinogenesis, chemical carcinogenesis, *EGFR* tyrosine kinase inhibitor resistance, FoxO signalling, proteoglycans in cancer and transcriptional deregulation in cancer.

Our analyses show that the diverse proteins participating in edgetic perturbations in cancer are essential biomolecules in tumorigenesis, that could be used for monitoring disease progression and developing new therapies. This integrated analysis is the first to utilize patient-specific PPIN derived from corresponding paired cancer and healthy mRNA expression profiles to decipher essential interactions distorted at the multi-cancer, cancer type, and subtype levels. Our findings present an integrated multi-omics approach for the computational identification of multi-cancer, cancer type and subtype-specific biomarkers with potential clinical prognostic relevance. As OMICS data become more complete, our methodology will be of increasing help in determining the full extent of protein network distortion across cancer types.

## Conclusion

In summary, our study presents a novel and robust scheme capable of identifying known and novel cancer-specific and multi-cancer biomarkers using patient-specific PPIN derived from mRNA expression data. Furthermore, the ability to determine uniquely distorted interactions whose participants are predictive of patient survival opens up the possibility to computationally obtain potential protein biomarkers for specific cancer types and subtypes. We also established that SMGs do not bring about the majority of perturbations in cancer PPINs. Additionally, we found probable novel biomarkers such as the THCA BRAF-like specific 4-gene signature biomarker (*ODAM, APP, IKBKG*, and *TOLLIP*). The THCA biomarkers may be essential for disease monitoring of THCA subtypes whereas the 14-gene signature (with *HRK* node perturbation) explicitly observed in KICH samples is a candidate for therapeutic targeting. Survival and functional enrichment analysis revealed that our candidate biomarkers are indeed involved in tumorigenesis. Our user-friendly portal will not only facilitate experimental research in the continued quest for druggable proteins at the protein-protein interaction network level but will also be essential for researchers to quickly mine and access the proteins involved in edgetic perturbations of cancer PPINs. We envisage that subsequent experimental validation will demonstrate the applicability of the novel biomarkers generated in this study for making informed clinical decisions as well as in developing cancer therapies. In the future, we will investigate patient-specific edgetic perturbations and determine proteins and corresponding isoforms (and protein domains) responsible for such disruptions.

## Materials and Methods

### Cancer datasets

We obtained RSEM^68^ quantified count data for healthy (non-cancer) as well as the corresponding cancer patient-specific mRNA expression profiles from the Broad Institute Web site (http://gdac.broadinstitute.org/). We further selected datasets with at least 10 paired healthy and cancer samples, covering 13 cancer types (Table S9). The corresponding cancer stage-specific annotated clinical data and subtype annotations were downloaded using the TCGABiolinks R package^69^. Stomach and esophageal carcinoma subtypes were downloaded from the supplementary materials of the TCGA consortium paper for STES^70^ because the Broad Institute Web site did not include all the paired samples. The clinical dataset consisted of patient samples grouped according to stages I, II, III and IV (Table S9).

### Global protein-protein interaction network (PPIN)

We obtained information on 330,557 binary interactions between human proteins from BioGRID^71^ and selected only those interactions whose individual interacting partners have a “reviewed” status in UniProt^72^. The resulting global network consisted of 224,223 human binary protein interactions between the total of 15,689 proteins. Based on the assumption that two proteins can only interact if proven to be translated, we further filtered the human interactome using protein abundance data. Whole-proteome high-confidence abundance data were obtained by combining information from PaxDb^73^ and The Human Proteome map^74^. Upon retaining only the proteins reported as translated (having non-zero abundance values) in both proteomics datasets our final PPIN consisted of 216,134 binary interactions involving 15,125 proteins. Hereafter, the total number of binary interactions in a PPIN is referred to as PPIN size.

### Patient- and cancer-specific protein interaction networks

Patient and cancer-specific PPIN were derived from gene expression data by PPIXpress^18^ using both PPINs described above. PPIXpress adapts PPINs to specific cellular conditions at the isoform level, thus enabling identification of tumor-related alterations missed by gene-level analysis. For each tissue type, we filtered the RNA-seq data to only include the genes that were consistently expressed across most samples using the EstimateExpression function of the xseq R package^75^. The function fits a mixture-of-Gaussian distributions model on the gene expression count data to distinguish between lowly expressed genes (presumed to be transcriptional noise) and biologically relevant gene expression (Fig. S6). Furthermore, for an isoform of a selected gene to be considered as expressed, its RSEM value was required to be 0.1 or higher. If multiple isoforms of a gene are expressed, the mean expression value of all isoforms is selected (running PPIXpress with ‘–g’ option).

### Patient-, cancer-, subtype-specific and multi-cancer perturbed edges

**Figure 8:**
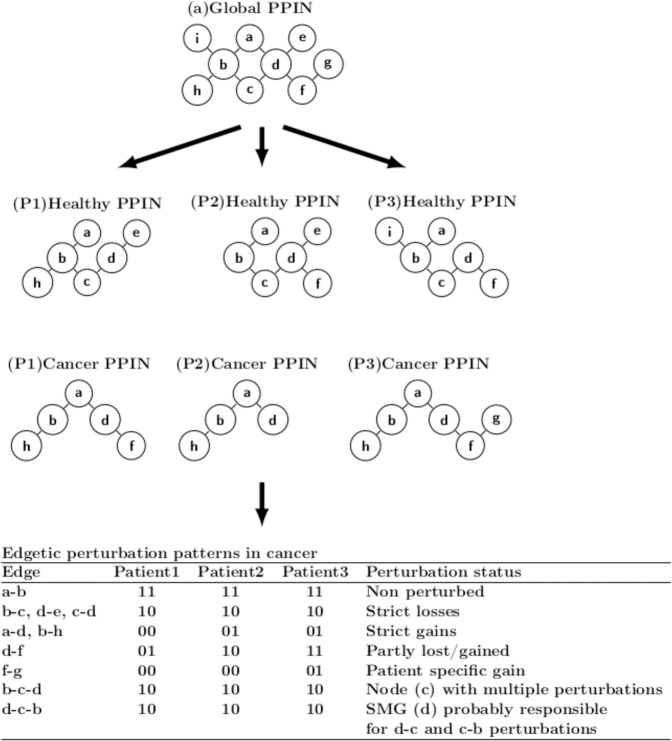
Edgetic perturbations in cancer. Assuming a global PPIN with 9 edges interconnecting 9 nodes and using cancer and healthy patient-specific mRNA expression profiles, for each patient (P1, P2 and P3) perturbed edges in cancer can be identified by comparing the healthy and the corresponding cancer PPIN. Significantly Mutated Genes (SMGs) may be involved in perturbation of edges directly interacting with them, or those interacting with their perturbed neighbors (secondary neighbors).

For each cancer PPIN we retrieved interactions that were not present in the paired healthy PPIN (gained edges). Likewise, in each healthy PPIN we identified interactions that were absent in the corresponding cancer PPIN (lost edges). Edges occurring in both healthy and cancer PPINs were considered non-perturbed. For brevity, lost, gained, and non-perturbed edges were assigned the codes 10, 01, and 11, respectively. For each patient, the set of all perturbed and non-perturbed edges represents their individual network perturbation profile. To obtain cancer type perturbation profiles we merged perturbation profiles of patients diagnosed with a specific type of cancer (Fig. 8). Edges that were not observed in one sample but observed in other samples were assigned the code 00 in the samples where they were absent, and either 01 or 10 when they were gained or lost, respectively; 11 represents unperturbed edges. On a cancer PPIN (Fig. 8), an edge can be gained across all patients (strict gains, edges a-d and b-h), lost across all patients (strict losses, edges b-c and d-e), partly gained or partly lost across patients (d-f), non-perturbed in all patients (a-b), or not observed in one patient but observed in others (f-g). The list of all the perturbed and non-perturbed edges in a single patient constitutes their perturbation profile. The union of all patient profiles diagnosed with a particular cancer type is referred to as a cancer perturbation profile. A cancer type perturbation profile is a list of lost, gained, and non-perturbed edges in all patients with a particular cancer type with their associated codes, as described above. For each cancer type *i*, each edge *j* was ranked depending on the percentage of samples it was gained (PercGained_i,j_) and lost (PercLost_i,j_) in (Table S7). A similar approach was undertaken for the cancer type, cancer subtype and multi cancer perturbations (see Fig. 8, Text S1, Tables S7 and S9 for details).

Finally, we sought to find prominent proteins frequently involved in edgetic perturbations as well as frequently perturbed edges at the multi-cancer, cancer type, and cancer subtype levels. At the cancer type and cancer subtype levels, proteins were ranked according to the number of perturbations they and their first network neighbors are involved in. Note that the perturbations associated with the second neighbors of a protein were counted if (i) the protein had at least two interacting partners and that the (ii) protein itself was associated with a perturbation. For instance, perturbation of edges b-c, c-d and d-e (Fig. 8) would give a rank of 3 for node c, and a rank of 1 each for nodes b, d and e.

### Identification of PPIN nodes associated with perturbations

We next searched for network nodes involved in edgetic perturbations. For each cancer type we merged all observed edges in the cancer and the corresponding healthy PPIN and then used DyNet^76^, a Cytoscape^77^ plugin, to identify the nodes associated with gained or lost edges. DyNet compares the nodes and edges present in two networks and then computes a rewiring metric score to determine which nodes have been rewired. To consider a node as rewired, we used a DyNet rewiring score of ≥0.5 and an edge count of ≥2, which corresponds to selecting the nodes with at least a degree (number of interaction partners) of 2 and showing perturbation of at least one edge. A single edge perturbation on a 2-degree node (2 interacting partners) means 50% of the edges are perturbed and thus have a rewiring score of 0.5.

### Clustering of cancers based on edgetic perturbation signatures

To understand the relationship between cancers in terms of their perturbation patterns, we used unsupervised clustering as implemented in the Pvclust^78^ R package to group cancers based on shared perturbations. Pvclust allows assessing the uncertainty of hierarchical clustering by performing multiscale bootstrap resampling and assigning p-values (as percentages) to clusters depending on how strong the cluster is supported by data. Pvclust provides two p-values: an approximately unbiased p-value (AU) computed from multiscale bootstrap resampling and a bootstrap probability (BP) computed by regular bootstrap resampling. High percentage values indicate a strong relationship of the cluster and the data, which may be biologically relevant. In our study, a cluster with an AU p-value > 0.95 (95%) or the significance level <0.05 was selected and kept for further analysis. In order to identify the edges defining the cluster, we searched for the edges perturbed across all cancer types within each cluster.

### Ranking of perturbed edges in terms of their importance in classifying cancer types

For the multi-cancer perturbations, after performing hierarchical clustering of the cancer types using the perturbed edges as features, we identified the edges appearing in only one cluster. Next, we used the random forest algorithm^79^ to identify the features (perturbed edges), which were most informative for attributing cancer types to the detected clusters based on shared edgetic perturbations. The Pvclust algorithm is essential in accurately identifying high confidence groups in data, and thus we did not face the problem of retraining our random forest algorithm to accurately classify cancer types sharing the majority of perturbed edges together. Our interest here was only to use the random forest algorithm (using the VarImp function from the R package caret^80^) to rank the features based on their importance in classifying cancer types into the groups detected during clustering. The VarImp function outputs feature ranking based on their mean squared error (MSE). Features with high MSE scores were then chosen to be the perturbed edges (features) having the highest weight in grouping the cancer types into the categories identified during clustering.

### Identification of Gene Ontology, KEGG pathways and disease-gene relations significantly enriched by proteins driving edgetic perturbations

To understand the biological relevance of the perturbed edges we identified statistically enriched Gene ontology (GO) terms and KEGG pathways associated with the proteins involved in edgetic perturbations. GO analysis was carried out using the R package topGO^81^ with statistical significance calculated using Fisher’s exact test. GO terms having a p-value of < 0.05 were chosen to be considerably enhanced. Additionally, significant GO terms were clustered using REVIGO^82^ to remove redundancy. Furthermore, a dispensability value (representing both the degree of redundancy and enrichment of a GO term) of <0.05 was considered significant after the REVIGO pruning step. To avoid statistical bias^83^ in the enrichment analysis of the proteins involved in edgetic losses we used all the genes expressed in cancer as the background for comparison. On the other hand, to analyze the GO terms and KEGG pathways enriched among the proteins involved in edgetic gains, we used all the genes expressed in the healthy (non-tumor) condition as the background for comparison. Disease-gene relation analysis was performed using DisGeNET^84^ implemented within the R package clusterProfiler^85^. KEGG pathway analysis was carried out using DAVID^86^.

### Predicting overall patient survival in cancer

Disease genes often work in concert and several studies have discovered network modules and hubs under attack in cancer^3,87,88^. To understand the importance of the proteins driving perturbations in cancer, we used SurvExpress^89^ to determine multi-gene cancer signatures and to assess their prognostic value for cancer. SurvExpress is a multi-gene cancer biomarker validation and discovery tool based on a wide collection of cancer datasets, including TCGA. From the ranked lists of perturbed edges in each cancer type, we selected each edge or a group of edges lost or gained across the largest number of patients as candidate biomarkers and analyzed them in SurvExpress.

### Implementation of the EdgeExplorer website

The EdgeExplorer website resides on a Linux server that provides Apache 2 for web services, SQLite for relational database management, and the PHP for server-side scripting services on the backend. The portal application further utilizes additional web technologies, among them: JavaScript, CSS, and jQuery.

### Randomisation of the PPIN

To check whether our results were brought about by changes in differential gene expression or were due to domain changes between the healthy and cancer state, we used the R package BiRewire first to generate a randomized network and then analyzed the resulting perturbations. We did this by building condition-specific PPINs in three randomly selected cancer types (BRCA, THCA and BLCA). BiRewire has the advantage of rewiring PPINs while preserving their functional connectivity and keeping the node degrees intact^90^.

### Statistical analyses

All statistical analyses were carried out in the in Python or the R environment. The Wilcoxon singed-rank test^91^ was used to determine if the mean of a healthy and the corresponding cancer PPIN sizes differed. For each cancer type, the chi-squared test^92^ was used to estimate the statistical significance of the extent of edgetic perturbations associated with cancer-specific significantly mutated genes (SMGs) compared to the extent of edgetic perturbations associated with genes having a similar network topology to the SMGs. Unsupervised hierarchical clustering using the Ward.D2 method and Euclidian distance^93^ was used to group cancer types. Patient stratification using Kaplan-Meier curves and log-rank test p-values for survival analysis were calculated using SurvExpress. For all analyses, p-values < 0.05 were considered significant.

## Supplementary information

Supplementary information.

Table S1.

Table S2.

Table S3.

Table S4.

Table S5.

Table S6.

Table S7.

Table S8.

Table S9.

## References

[1] Kandoth C (2013). Mutational landscape and significance across 12 major cancer types. Nat..

[2] Sharma S, Kelly TK, Jones PA (2010). Epigenetics in cancer. Carcinogenesis.

[3] Jonsson PF, Bates PA (2006). Global topological features of cancer proteins in the human interactome. Bioinforma..

[4] Sun J, Zhao Z (2010). A comparative study of cancer proteins in the human protein-protein interaction network. BMC Genomics.

[5] Nishi H (2013). Cancer Missense Mutations Alter Binding Properties of Proteins and Their Interaction Networks. PLoS One.

[6] Buljan M, Blattmann P, Aebersold R, Boutros M (2018). Systematic characterization of pan‐cancer mutation clusters. Mol. Syst. Biol..

[7] Zhao J, Cheng F, Zhao Z (2017). Tissue-Specific Signaling Networks Rewired by Major Somatic Mutations in Human Cancer Revealed by Proteome-Wide Discovery. Cancer Res..

[8] Bowler EH, Wang Z, Ewing RM (2015). How do oncoprotein mutations rewire protein-protein interaction networks?. Expert. Rev. Proteom..

[9] Cheng F (2014). Studying tumorigenesis through network evolution and somatic mutational perturbations in the cancer interactome. Mol. Biol. Evol..

[10] Yan W, Xue W, Chen J, Hu G (2016). Biological Networks for Cancer Candidate Biomarkers Discovery. Cancer Inf..

[11] Cui, H., Zhao, N. & Korkin, D. Multilayer View of Pathogenic SNVs in Human Interactome through *in-silico* Edgetic Profiling. *Journal of Molecular Biology*, 10.1016/j.jmb.2018.07.012 (2018). 10.1016/j.jmb.2018.07.012 30017919

[12] Li Z (2017). The OncoPPi network of cancer-focused protein-protein interactions to inform biological insights and therapeutic strategies. Nat. Commun..

[13] Patil A, Kinoshita K, Nakamura H (2010). Hub promiscuity in protein-protein interaction networks. Int. J. Mol. Sci..

[14] Chuang H-Y, Lee E, Liu Y-T, Lee D, Ideker T (2007). Network-based classification of breast cancer metastasis. Mol. Syst. Biol..

[15] Alcaraz N (2017). De novo pathway-based biomarker identification. Nucleic Acids Res..

[16] Vitting-Seerup K, Sandelin A (2017). The Landscape of Isoform Switches in Human Cancers. Mol. Cancer Res..

[17] Sebestyén E, Zawisza M, Eyras E (2015). Detection of recurrent alternative splicing switches in tumor samples reveals novel signatures of cancer. Nucleic Acids Res..

[18] Will T, Helms V (2016). PPIXpress: construction of condition-specific protein interaction networks based on transcript expression. Bioinforma..

[19] Ghadie MA, Lambourne L, Vidal M, Xia Y (2017). Domain-based prediction of the human isoform interactome provides insights into the functional impact of alternative splicing. PLoS Comput. Biol..

[20] Danielsson F (2013). Majority of differentially expressed genes are down-regulated during malignant transformation in a four-stage model. Proc. Natl. Acad. Sci. USA.

[21] Duijf PHG, Schultz N, Benezra R (2013). Cancer cells preferentially lose small chromosomes. Int. J. Cancer.

[22] Flavahan, W. A., Gaskell, E. & Bernstein, B. E. Epigenetic plasticity and the hallmarks of cancer. *Science* **357** (2017). 10.1126/science.aal2380 PMC5940341 28729483

[23] Anglani R (2014). Loss of connectivity in cancer co-expression networks. PLoS One.

[24] Cordero D (2014). Large differences in global transcriptional regulatory programs of normal and tumor colon cells. BMC Cancer.

[25] Climente-González H, Porta-Pardo E, Godzik A, Eyras E (2017). The Functional Impact of Alternative Splicing in Cancer. Cell Rep..

[26] Edfors, F. *et al*. Gene‐specific correlation of RNA and protein levels in human cells and tissues. *Mol Syst Biol* **12** (2016). 10.15252/msb.20167144 PMC5081484 27951527

[27] Liu Y, Beyer A, Aebersold R (2016). On the Dependency of Cellular Protein Levels on mRNA Abundance. Cell.

[28] Latysheva NS (2016). Molecular Principles of Gene Fusion Mediated Rewiring of Protein Interaction Networks in Cancer. Mol. Cell.

[29] Zhang, X.-F. *et al*. Comparative analysis of housekeeping and tissue-specific driver nodes in human protein interaction networks. *BMC Bioinformatics* **17** (2016). 10.1186/s12859-016-1233-0 PMC5016887 27612563

[30] Kim J, Kim I, Han SK, Bowie JU, Kim S (2012). Network rewiring is an important mechanism of gene essentiality change. Sci. Rep..

[31] Poornima P, Kumar JD, Zhao Q, Blunder M, Efferth T (2016). Network pharmacology of cancer: From understanding of complex interactomes to the design of multi-target specific therapeutics from nature. Pharmacol. Res..

[32] Jurca G (2016). Integrating text mining, data mining, and network analysis for identifying genetic breast cancer trends. BMC Res. Notes.

[33] Ramsköld D, Wang ET, Burge CB, Sandberg R (2009). An abundance of ubiquitously expressed genes revealed by tissue transcriptome sequence data. PLoS Comput. Biol..

[34] Yu NY-L (2015). Complementing tissue characterization by integrating transcriptome profiling from the Human Protein Atlas and from the FANTOM5 consortium. Nucleic Acids Res..

[35] Uhlén M (2016). Transcriptomics resources of human tissues and organs. Mol. Syst. Biol..

[36] Davidson SM, Heiden MGV (2017). Critical Functions of the Lysosome in Cancer Biology. Annu. Rev. Pharmacology Toxicol..

[37] Mirzaei, H. & Faghihloo, E. Viruses as key modulators of the TGF-β pathway; a double-edged sword involved in cancer. *Rev. Med. Virol*. **28** (2018). 10.1002/rmv.1967 PMC7169117 29345394

[38] Mosesson Y, Mills GB, Yarden Y (2008). Derailed endocytosis: an emerging feature of cancer. Nat. Rev. Cancer.

[39] Kingston D (2011). Inhibition of retromer activity by herpesvirus saimiri tip leads to CD4 downregulation and efficient T cell transformation. J. Virol..

[40] Rajagopalan D, Jha S (2018). An epi(c)genetic war: Pathogens, cancer and human genome. Biochim. Biophys. Acta.

[41] Kim J, Yao F, Xiao Z, Sun Y, Ma L (2018). MicroRNAs and metastasis: small RNAs play big roles. Cancer Metastasis Rev..

[42] Dhillon AS, Hagan S, Rath O, Kolch W (2007). MAP kinase signalling pathways in cancer. Oncogene.

[43] Muthuswamy SK, Xue B (2012). Cell Polarity As A Regulator of Cancer Cell Behavior Plasticity. Annu. Rev. Cell Dev. Biol..

[44] Hanahan D, Weinberg RA (2011). Hallmarks of cancer: the next generation. Cell.

[45] Jørgensen, J. T. A paradigm shift in biomarker guided oncology drug development. *Annals of Translational Medicine* **7** (2019). 10.21037/atm.2019.03.36 PMC6511563 31157269

[46] Liu Q, Dai S-J, Li H, Dong L, Peng Y-P (2014). Silencing of NUF2 inhibits tumor growth and induces apoptosis in human hepatocellular carcinomas. Asian Pac. J. Cancer Prev..

[47] Hu P, Shangguan J, Zhang L (2015). Downregulation of NUF2 inhibits tumor growth and induces apoptosis by regulating lncRNA AF339813. Int. J. Clin. Exp. Pathol..

[48] Ardini E (2016). Entrectinib, a Pan-TRK, ROS1, and ALK Inhibitor with Activity in Multiple Molecularly Defined Cancer Indications. Mol. Cancer Ther..

[49] Jin Z, Kotera M, Goto S (2014). Virus proteins similar to human proteins as possible disturbance on human pathways. Syst. Synth. Biol..

[50] Zhao M, Kim P, Mitra R, Zhao J, Zhao Z (2016). TSGene 2.0: an updated literature-based knowledgebase for tumor suppressor genes. Nucleic Acids Res..

[51] Andor N (2016). Pan-cancer analysis of the extent and consequences of intra-tumor heterogeneity. Nat. Med..

[52] Reiter JG (2018). Minimal functional driver gene heterogeneity among untreated metastases. Sci..

[53] Vandin, F. Computational Methods for Characterizing Cancer Mutational Heterogeneity. *Front Genet* **8** (2017). 10.3389/fgene.2017.00083 PMC5469877 28659971

[54] Yuan Y (2014). Assessing the clinical utility of cancer genomic and proteomic data across tumor types. Nat. Biotechnol..

[55] Hsia DA (2010). KDM8, a H3K36me2 histone demethylase that acts in the cyclin A1 coding region to regulate cancer cell proliferation. PNAS.

[56] Lu C (2016). Histone H3K36 mutations promote sarcomagenesis through altered histone methylation landscape. Sci..

[57] Lee J-C, Liang C-W, Fletcher CD (2017). Giant cell tumor of soft tissue is genetically distinct from its bone counterpart. Mod. Pathol..

[58] Greenman C (2007). Patterns of somatic mutation in human cancer genomes. Nat..

[59] Bozic I (2010). Accumulation of driver and passenger mutations during tumor progression. Proc. Natl. Acad. Sci. USA.

[60] Li X (2016). Dynamic changes of driver genes’ mutations across clinical stages in nine cancer types. Cancer Med..

[61] Barabási A-L, Gulbahce N, Loscalzo J (2011). Network medicine: a network-based approach to human disease. Nat. Rev. Genet..

[62] Leiserson MDM (2015). Pan-cancer network analysis identifies combinations of rare somatic mutations across pathways and protein complexes. Nat. Genet..

[63] Shi X (2017). CyNetSVM: A Cytoscape App for Cancer Biomarker Identification Using Network Constrained Support Vector Machines. PLoS One.

[64] Fouad YA, Aanei C (2017). Revisiting the hallmarks of cancer. Am. J. Cancer Res..

[65] Pfoh R, Lacdao IK, Saridakis V (2015). Deubiquitinases and the new therapeutic opportunities offered to cancer. Endocr. Relat. Cancer.

[66] Lee O-H (2015). Role of the focal adhesion protein TRIM15 in colon cancer development. Biochim. Biophys. Acta.

[67] Green DR (2016). A BH3 Mimetic for Killing Cancer Cells. Cell.

[68] Li B, Dewey CN (2011). RSEM: accurate transcript quantification from RNA-Seq data with or without a reference genome. BMC Bioinforma..

[69] Colaprico, A. *et al*. TCGAbiolinks: an R/Bioconductor package for integrative analysis of TCGA data. *Nucleic Acids Res*., 10.1093/nar/gkv1507 (2015). 10.1093/nar/gkv1507 PMC4856967 26704973

[70] The Cancer Genome Atlas Research Network. (2017). Integrated genomic characterization of oesophageal carcinoma. Nat..

[71] Chatr-Aryamontri A (2015). The BioGRID interaction database: 2015 update. Nucleic Acids Res..

[72] Bateman A (2017). UniProt: the universal protein knowledgebase. Nucleic Acids Res..

[73] Wang M (2012). PaxDb, a database of protein abundance averages across all three domains of life. Mol. Cell Proteom..

[74] Kim M-S (2014). A draft map of the human proteome. Nat..

[75] Ding J (2015). Systematic analysis of somatic mutations impacting gene expression in 12 tumour types. Nat. Commun..

[76] Goenawan IH, Bryan K, Lynn DJ (2016). DyNet: visualization and analysis of dynamic molecular interaction networks. Bioinforma..

[77] Shannon P (2003). Cytoscape: A Software Environment for Integrated Models of Biomolecular Interaction Networks. Genome Res..

[78] Suzuki R, Shimodaira H (2006). Pvclust: an R package for assessing the uncertainty in hierarchical clustering. Bioinforma..

[79] Breiman L (2001). Random Forests. Mach. Learn..

[80] Kuhn M (2008). Building Predictive Models in R Using the caret Package. J. Stat. Softw..

[81] Alexa, A. & Rahnenfuhrer, J. Gene set enrichment analysis with topGO. 26.

[82] Supek F, Bošnjak M, Škunca N, Šmuc T (2011). REVIGO summarizes and visualizes long lists of gene ontology terms. PLoS One.

[83] Timmons JA, Szkop KJ, Gallagher IJ (2015). Multiple sources of bias confound functional enrichment analysis of global -omics data. Genome Biol..

[84] Piñero J (2017). DisGeNET: a comprehensive platform integrating information on human disease-associated genes and variants. Nucleic Acids Res..

[85] Yu G, Wang L-G, Han Y, He Q-Y (2012). clusterProfiler: an R Package for Comparing Biological Themes Among Gene Clusters. OMICS.

[86] Huang DW (2007). DAVID Bioinformatics Resources: expanded annotation database and novel algorithms to better extract biology from large gene lists. Nucleic Acids Res..

[87] Patel VN (2013). Network Signatures of Survival in Glioblastoma Multiforme. PLoS Computational Biol..

[88] Cao, Z. & Zhang, S. An integrative and comparative study of pan-cancer transcriptomes reveals distinct cancer common and specific signatures. *Sci Rep* **6** (2016). 10.1038/srep33398 PMC5025752 27633916

[89] Aguirre-Gamboa, R. *et al*. SurvExpress: An Online Biomarker Validation Tool and Database for Cancer Gene Expression Data Using Survival Analysis. *Plos**One* **8** (2013). 10.1371/journal.pone.0074250 PMC3774754 24066126

[90] Gobbi A (2014). Fast randomization of large genomic datasets while preserving alteration counts. Bioinforma..

[91] Dexter F (2013). Wilcoxon-Mann-Whitney test used for data that are not normally distributed. Anesth. Analg..

[92] Yates F (1934). Contingency Tables Involving Small Numbers and the χ2 Test. Suppl. J. R. Stat. Soc..

[93] Nagahashi, M. *et al*. Genomic landscape of colorectal cancer in Japan: clinical implications of comprehensive genomic sequencing for precision medicine. *Genome Med* **8** (2016). 10.1186/s13073-016-0387-8 PMC5180401 28007036

