# Author Correction: Edgetic perturbation signatures represent known and novel cancer biomarkers

**DOI:** 10.1038/s41598-021-82646-x

**Published:** 2021-02-05

**Authors:** Evans Kataka, Jan Zaucha, Goar Frishman, Andreas Ruepp, Dmitrij Frishman

**Affiliations:** 1 Department of Bioinformatics, Wissenschaftszentrum Weihenstephan, Technische Universität München, Maximusvon-Imhof-Forum 3, 85354 Freising, Germany; 2 Laboratory of Bioinformatics, RASA Research Center, St Petersburg State Polytechnic University, St Petersburg, Russia 195251; 3 Institute of Experimental Genetics (IEG), Helmholtz Zentrum München-German Research Center for Environmental Health (GmbH), Ingolstädter Landstrasse 1, 85764 Neuherberg, Germany

Correction to: *Scientific Reports* https://doi.org/10.1038/s41598-020-61422-3, published online 09 March 2020

This Article contains errors in Figure 2, Figure 3 and Table S9.

In Figure 2, the colours and the positions of the labels are incorrect. The correct Figure 2 appears below as Figure 1. As a result, the legend of Figure 2,

**Figure 1:**
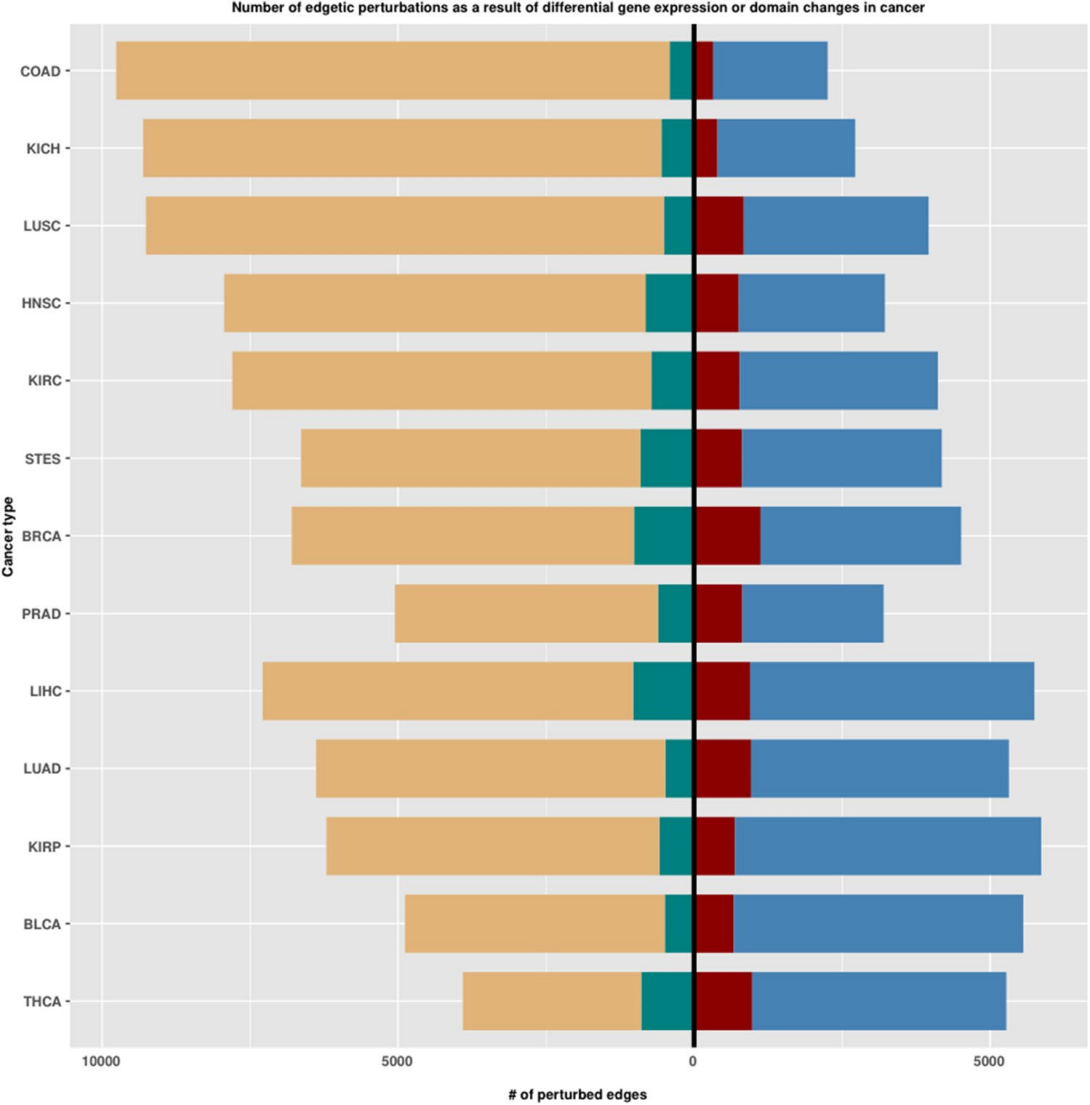
A correct version of the original Figure 2. Bar plots indicating the number of edgetic perturbations obtained as a result of gene expression changes or domain changes that come about after isoform switches between cancer and healthy states. Sky blue: edgetic gains as a result of more genes being expressed in the cancer state, dark brown (left of zero intercept): edgetic gains as a result of isoform/domain changes (left of zero intercept). Light brown: edgetic losses as a result of the depletion of genes in the cancer state (right of zero intercept), light green: edgetic losses as a result of isoform/domain changes (right of zero intercept).

“Sky blue: edgetic gains as a result of more genes being expressed in the cancer state, dark brown (left of zero intercept): edgetic gains as a result of isoform/domain changes (left of zero intercept). Light brown: edgetic losses as a result of the depletion of genes in the cancer state (right of zero intercept), light green: edgetic losses as a result of isoform/domain changes (right of zero intercept).”

should read:

“Sky blue: edgetic gains as a result of more genes being expressed in the cancer state, red: edgetic gains as a result of isoform/domain changes (right of zero intercept): Light brown: edgetic losses as a result of the depletion of genes in the cancer state (left of zero intercept), light green: edgetic losses as a result of isoform/domain changes (left of zero intercept).”

In Figure 3, the labels of the gene CALM1 are incorrectly given as CALM2. The correct Figure 3 appears below as Figure 2.

**Figure 2:**
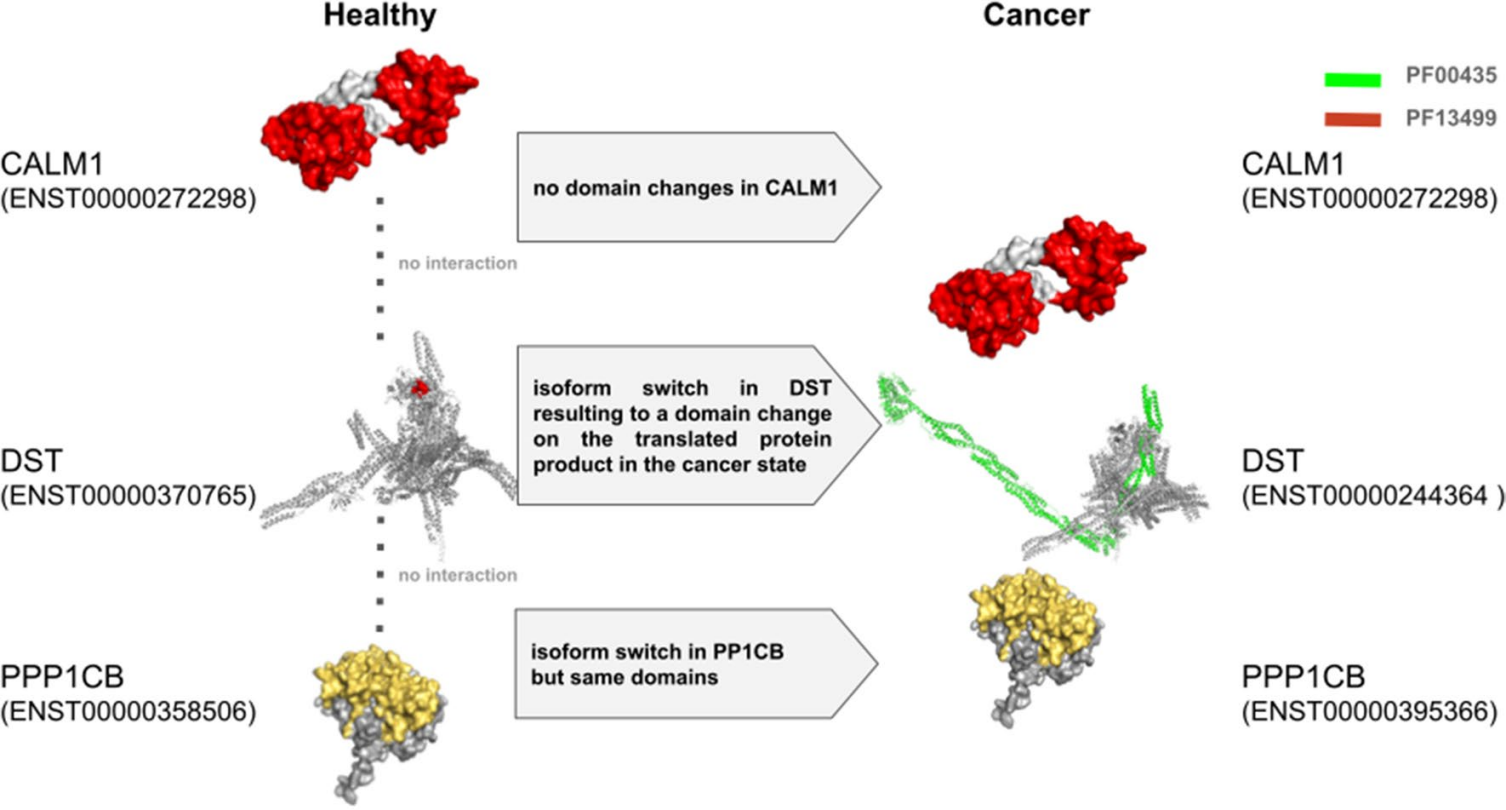
A correct version of the original Figure 3. An example showing the consequences of domain changes between the cancer state and healthy state in patients diagnosed with BRCA. The protein structures of both (P0DP23) *CALM1* and (P62140) *PP1CB* were obtained from PDB while those of *DST* were modelled using the ensemble transcript sequences in SWISS-MODEL and visualized in PyMol. Following an isoform switch from ENST00000370765 (in healthy) to ENST00000244364 (in cancer), the protein Q03001 (*DST*) gained the domain PF13499. The consequence is the gain of interactions with the genes *PPP1CB* and *CALM1*.

Table S9 contains errors as a result of auto conversion in Excel. The correct Table S9 is provided below.

## Supplementary Information

Supplementary Table.

