# Supplementary material for: Edgetic perturbation signatures represent known and novel cancer biomarkers
Source: Sci Rep. 2020 Mar 9;10:4350. doi: 10.1038/s41598-020-61422-3 (PMC7062722; doi:10.1038/s41598-020-61422-3)
Supplement: Supplementary file 1 — Supplementary information. [file 41598_2020_61422_MOESM1_ESM.pdf]

Edgetic perturbation signatures represent known and novel cancer biomarkers

Evans Kataka[1], Jan Zaucha[1], Goar Frishman[3], Andreas Ruepp[3], and Dmitrij Frishman[1,2,*]
[1] Department of Bioinformatics, Wissenschaftszentrum Weihenstephan, Technische Universität München, Maximus-von-Imhof-Forum 3, 85354 Freising, Germany
[2] Laboratory of Bioinformatics, RASA Research Center, St Petersburg State Polytechnic University, St Petersburg, 195251, Russia.
[3] Institute of Bioinformatics and Systems Biology (IBIS), Helmholtz Zentrum München-German Research Center for Environmental Health (GmbH), Ingolstädter Landstrasse 1, D-85764 Neuherberg, Germany.

* To whom correspondence should be addressed. Tel: [+498161712134]; Fax: [+498161712186], Email: [d.frishman@wzw.tum.de]

Department of Bioinformatics, Wissenschaftszentrum Weihenstephan, Technische Universität München, Maximus-von-Imhof-Forum 3, 85354 Freising, Germany

Identification of cancer type, cancer subtype-specific and multi-cancer perturbed edges

For each cancer type we merged all edges lost or gained in any of the samples and retrieved only those edges that are perturbed at least once in this specific cancer type and are observed in at least two samples (for example edges a-d and b-h (gained edges) and edges d-e and c-d (lost edges) in Fig. 8). Note that edges perturbed in a cancer type but observed only in a single patient sample are considered patient-specific.

For each cancer with a subtype *s* (Table S9), we also searched for perturbations unique only to a particular subtype and ranked each perturbed edge *j* depending on the percentage of samples it was gained ($SubtypePercGained_{s,j}$) and lost ($SubtypePercLost_{s,j}$) in, with $j \geq 2$. Note that a cancer subtype perturbation profile is a subset of the corresponding cancer type perturbation profiles involving the patients diagnosed with this particular subtype.

To identify perturbations occurring across multiple cancers, we first merged all edges perturbed in each cancer type and then identified only those perturbations observed in at least two cancer types. If, instead of the three patients P1, P2, and P3, the perturbation patterns in Fig. 8 corresponded to three different cancer types C1, C2, and C3, edges a-d and b-h would represent multi-cancer gained edges while edges d-e and c-d would represent multi-cancer lost edges since they are perturbed in more than two cancer types. Also, for each cancer type *i*, each perturbed edge *j* was ranked depending on the percentage of cancer types it was gained ($MultiCanGained_{ij}$) or lost ($MultiCanLost_{ij}$) in, with $i \geq 2$. We then ranked these multi-cancer perturbations based on the number of cancer types exhibiting them (Table S7).

EdgeExplorer web portal annotations

To identify gene-disease relations with experimental evidence, we did manual annotation by searching for publications implicating anomalies of each query gene in affecting cancer progression or treatment outcomes. The following annotation rules were followed:

1. Search for gene – cancer-type associations in recent experimental papers indexed by PubMed, PMC and Google Scholar while using all synonyms of a gene name. If no full text of the articles were found, we further searched in the Bavarian State Library database.

2. Priority was first given to journal papers with experimental evidence demonstrating the association of the exact gene with the particular cancer type in which edgetic perturbations were detected. The associations included: gene mutation or differential expression of a gene in tumor samples when compared to normal samples, or gene involvement in metastasis, patient survival, prognosis, therapy resistance or therapy success. Results reporting findings ased on TCGA data were excluded from the annotations unless no other hits were found for that gene (see 4).

3. If there was no such information, the second priority was given to papers with experimental evidence demonstrating the association between the specific gene and a cancer type occurring in the same somatic tissue. For example, if there was no information on KIRP but there was for KIRC, we report that association.

4. Finally, if there was no such information, as a third priority, we checked for gene-disease association in The Cancer Genome Atlas to show if indeed our study yields similar results to other studies that have previously used TCGA data.

Generation of genes having similar node degrees as SMGs and their associated perturbations

To comprehend whether SMGs were pivotal in edgetic perturbations, we compared the proportions of perturbations involving SMGs and those from randomly generated genes with a similar degree of interacting proteins. First, we downloaded lists of pan-cancer and cancer specific significantly mutated cancer genes from the COSMIC Cancer Gene Census (https://cancer.sanger.ac.uk/census)[5] and from the TCGA consortium (https://cancergenome.nih.gov/publications)[6]. The genes amounted to 719 and 299 cancer genes from COSMIC and Bailey *et. al*, respectively, and were classified according to their significance as cancer specific or as pan-cancer. We considered a gene to be significantly mutated in a certain cancer type if it was characterized as either cancer specific or pan-cancer, but affected that cancer type. Finally, in each cancer type, a union of the significantly mutated genes from both the above sources were considered as cancer specific significantly mutated genes (Table S3). To find the number of perturbations involving the SMGs, we searched for any perturbed interactions having an SMG as an interacting partner. Then, in each cancer type, we merged all the interactions observed in both cancer and healthy in all the patients to generate all possible interactions within a cancer type. Next, for each cancer type, we determined the degree of each of the proteins within all the possible interactions of a cancer type. We used the degree of each SMG involved in any perturbation to randomly query for other proteins having a similar number of interacting partners to them (Table S3). Finally, we determined the number of perturbations associated with the genes having a similar degree to the SMGs.
For each cancer type, the Z-test of proportions[7] was used to estimate the statistical significance of the extent of edgetic perturbations associated with cancer-specific SMGs compared to the extent of edgetic perturbations associated with genes having a similar network topology to the SMGs. To do this, we first determined if there were significant differences in the proportion of perturbations associated with SMGs and the proportion of perturbations associated with randomly generated genes. Then, for each significant difference, we sought to find only the cancer types where the proportion of perturbations associated with SMGs were significantly larger than those associated with the randomly generated genes.

Significantly mutated genes together with proteins having high degrees of connectivity in the PPIN are crucial players in edgetic perturbations of cancer PPINs

Elevated mutation rate is a hallmark of cancer driver genes[8–10]. We analysed the involvement of SMGs as well as their first and second network neighbours in edgetic perturbations. Leiserson *et al.* previously suggested that somatic mutations affect subnetworks within PPINs via a heat diffusion model where "hot" nodes/SMGs propagate their heat to neighbouring nodes[11]. First, we found that not all SMGs are involved in edgetic perturbations, but only a specific number in each cancer type (Table S3a). Also, there were significant differences in the proportion of perturbations associated with SMGs and those associated with the randomly generated genes having similar node degrees in the PPINs. A majority of the perturbations across the cancer types had more instances where the portion of the perturbations associated with random genes was more substantial than the proportion of perturbations associated with SMGs. This observation was prominent in BRCA, PRAD and STES where the portion of the perturbations associated with random genes at both the first and second neighbours was significant, while HNSC had no significant differences in the two proportions (Table S3b). However, a look into the proteins involved in the majority of the perturbations associated with the random genes (e.g., *SKIP, HIST1H3J* and *EZH2*) revealed that the proteins function in gene expression deregulation in cancer and are potential molecules for therapeutic intervention in cancer[12–18]. With a rise in the interest of therapeutic targeting of cancer enabling proteins at the PPIN level, our findings suggest that therapeutic targeting of only SMGs involved in edgetic perturbations particularly in BRCA, PRAD, STES and HNSC may not yet be a sound idea. However, additional incorporation of epigenetic markers engaged in tumourigenesis of these cancer types may be additionally beneficial as previously suggested [19].

Nevertheless, we found that in 9 out of 13 cancer types, edgetic perturbations were associated with the SMGs ($p < 0.05$, Table S3c) as compared to edgetic perturbations resulting from randomly generated genes with similar network topologies. Our findings correspond to those of [20–21] who pointed out that somatic mutations occurring at protein interaction interfaces may alter protein-protein interaction networks for example by resulting in loss of interactions or gain of new interactions. Besides, Cui *et al.* while analysing the effects of somatic mutations on the PPIN of liver cancer patients found that SMGs significantly rewire liver cancer PPINs when compared to random non mutated genes[22]. In these 9 cancer types listed above, the instances showing significant perturbations attributed to the SMGs provide opportunities for therapeutic targeting at the PPIN level as is in the case with BH3 like proteins[23] .

Proteins involved in edgetic perturbations affect the overall patient survival and can serve as cancer type biomarkers

BRCA. We found that proteins involved in both edgetic gains and losses (e.g., *CDC25C, NOS2*, and *FOXF1*) contribute to BRCA tumorigenesis as previously suggested[24–25]. We further observed that most patients showing significant edgetic perturbations were at a higher risk of BRCA than those who did not have such edgetic perturbations. For example, the edge between the regulator of nonsense transcripts 2 and heparan sulfate 3-O-sulfotransferase 3A1 (*UPF2-HS3ST3A1)* was specifically gained in BRCA patients, and all of them (110/110) were predicted to be at high risk of BRCA related death (shorter lifespan). Our findings also corroborate previous research indicating the cell and tumor specificity of *HS3ST3A1* in BRCA tumorigenesis[26].

LUAD. The primary protein involved in LUAD specific edgetic gains, mitochondrial 2-oxodicarboxylate carrier (*SLC25A21,* an ornithine decarboxylate carrier), was more significant in predicting a majority of LUAD patients as being at a higher risk of LUAD related death than

any other proteins involved in the other perturbations. Tian *et al.* have shown the existence of elevated levels of ornithine decarboxylate (ODC) and polyamines in lung cancer[27] while Kumar *et al.* have shown that targeting ornithine decarboxylase and related pathways by the agent DMFO/Eflornithine prevents tumor and adenocarcinoma formation in mice infected with lung cancer[28]. Since we identified *SLC25A21* perturbations as being specific to LUAD, our findings suggest that *SLC25A21* and three of its interacting partners (*PPIE, FBX06,* and *NOSIP*) may be essential biomarkers in LUAD and targets for LUAD chemoprevention.

LUSC. Proteins involved in both edgetic losses and gains may be important in LUSC tumorigenesis. For instance, our study indicates that the mediator of RNA polymerase II transcription subunit 12-like protein (*MED12L)*, a lung cancer marker previously associated with carboplatin-induced cytotoxicity in cancer patients of African descent[29] could be a multiracial lung cancer biomarker and specifically vital for LUSC subtype. While our study revealed that LUAD and LUSC shared a high proportion of edgetic losses, we also found perturbations harbouring proteins distinguishing the two non-small cell lung cancer types. For example, while previous research has linked significant mutation of the T-cell surface glycoprotein CD1b (*CD1B)* protein to non-small cell lung cancer types[30], our study further suggests that *CD1B* may be more relevant to LUSC.

PRAD. Even though there was no data for deceased patients in the PRAD cohort, our analysis revealed at least 13 out of 52 patients that showed a higher risk of PRAD related death as a consequence of the proteins involved in edgetic perturbations. For instance, we found eight patients carrying perturbations affecting the homeobox protein DLX-2 (*DLX2)* that was explicitly gained in PRAD cancer type, as being at a high risk of PRAD related death. *DLX2* is a novel epigenetic marker used in the identification of PRAD patients for active surveillance[31]. Also, we found an additional patient predicted to be at high risk of PRAD-related death following disruptions involving the galectin-9C (*LGALS9C)* protein. While a recent study identified *galectin-9* as an anti-cancer agent[32], the authors could not confirm if *LGALS9C* or *LGALS9B* (*galectin-9* like proteins) were also anti-cancer agents. Our research suggests otherwise, and implicates *LGALS9C* in tumorigenesis.

KIRC. Proteins involved in both edgetic gains and losses appear to be essential in KIRC tumorigenesis since a significant number of patients showed a high risk of KIRC-related death (Table S4 and Fig. S2). Additionally, we discovered KIRC specific edge losses involving the bcl-2-interacting killer protein (*BIK),* which was previously reported as a landmark in KIRC oncogenesis[33].

KIRP. Gene expression changes of the proteins involved in both edgetic gain and loss perturbations could predict overall patient survival, and these proteins have already been found to be critical in cancer progression. For instance, high levels of expression of the protein ribonucleoprotein IMP3 (*IGF2BP3)* is an indicator of kidney tumors more likely to undergo distant metastasis. Moreover, *IGF2BP3* is an independent prognostic marker in kidney cancers[34]. The role of *ASB14* in cancer is largely unknown; however, some ASB proteins have been shown to be involved in cancer progression (e.g., *ASB3*, *ASB8* and *ASB16* in Kidney cancer)[35]. Our study may be the first to link *ASB14* to kidney cancer: we found *ASB14* and its interactors to be prognostic in KIRP (p =1.52e-08, Table S4 and Fig. S2), making it a viable candidate for experimental validation given the recent knowledge of the role of *ASB* proteins in other types of cancer. Also, our findings agree with those of Prestin *et al.* who showed the deregulation of the nuclear receptor subfamily 0 group B member 1 (*NROB2)* protein to be an important step in renal cancer progression[36]. Additionally, edgetic loss between dickkopf-related protein 1 and MyoD family inhibitor (*DKK1-MDFI),* proteins involved in Wnt signalling[37,38], may suggest that deregulation of the Wnt Signalling pathway is a vital event in

KIRP. Since *DKK1* is a tumor suppressor[39], its perturbation in KIRP may be an indicator of why most patients showing this edgetic loss perturbation were at a higher risk of KIRP related death.

COAD. Proteins involved in both edgetic gains and losses may be essential in COAD tumorigenesis. Our results agree with previous works linking, for instance, overexpression of the melanocyte-specific protein 1 (*CITED1)* to reduced patient survival in intestinal tumors[40] and the voltage-gated calcium channel subunit alpha protein (*CACNA1A)* to patient survival as well as drug resistance in colorectal cancer[41].

THCA. Proteins involved in both edgetic gain and loss perturbations are engaged in THCA tumorigenesis. Also, our study revealed probable and, to the best of our knowledge, hitherto unknown THCA biomarkers (*RAB40A* and *CSAG1)*. However, the ras-related protein Rab-40A (*RAB40A)* has been shown to participate in ubiquitination and migration in high-grade breast cancer samples[42] while the expression changes of the chondrosarcoma-associated gene 1 protein (*CSAG1)*, a cancer-testis antigen, has been reported to be a signature in some human cancer cell lines[43]. Additionally, other cancer testis antigens are prevalent in thyroid malignancies, but their biological roles are still unclear[44].

HNSC. We found that proteins involved in both edgetic loss and gain perturbations participate in HNSC progression. For instance, we found an 11-gene (*WNK4, SGK1, KLHL2, HSP90AA1, YWAHQ, AKT1, BCL6, CUL3, NEDD4L, STK39, KLHL3*) HNSC-specific loss perturbation signature with the serine/threonine-protein kinase WNK4 *(WNK4)* losing interactions with all the other 10 genes. *WNK4* mutations result in hyperkalemia, cell permeability[45] and recruitment of claudin proteins which promote metastasis in cancer[46]. Additionally, cullin 3 (*CUL3)* has been linked to HNSC metastasis and drug resistance[47]. Our study, therefore, presents a multi-gene HNSC specific biomarker that may be of use in clinical monitoring and therapy decision making.

STES. Proteins involved in edgetic losses (e.g., *HSPA1L*) may be more oncogenic than those involved in edgetic gains: twice as many STES patients were predicted to be at a higher risk of STES related death by the proteins engaged in edgetic losses. The perturbation of the heat shock protein (*HSPA1L/HSP70-hom)* in our analysis supports the current knowledge of the deregulation of *HSP70* anti-apoptotic family members in gastric cancers. *HSP70* proteins are pivotal in the folding of proteins or refolding of denatured proteins and have been shown to be prognostic in gastric cancers[48]. Our study, therefore, additionally supports that *HSPA1L* may also be a therapeutic target for STES.

LIHC. Survival analysis revealed that proteins involved in both edgetic gains and losses may participate in tumor growth and are essential for patient stratification. Our findings corroborate previous research linking increased expression of the protein Wnt-3a (*WNT3A)* to tumor cell proliferation in LIHC[49]. We found a 14-gene edgetic gain perturbation biomarker consisting of *WNT3A, HSPA5, LRP6, CANX, TRAF2, FZD2, FZD1, KCTD1, PPP2R1B, PPP2R5D, PPP2R5A, PPP2R5B, PPP2R5E,* and *PPP2R2D*. This 14-gene signature presents a biomarker for probable therapy targeting via microRNA-195, as previously suggested[49].

BLCA. Our results suggest that proteins involved in both edgetic gains and losses are essential biomarkers in BLCA tumorigenesis and represent candidate BLCA biomarkers. For instance, perturbations involving the histone protein *HIST2H2AC* may be responsible for the epigenetic changes in BLCA tumorigenesis. Accumulation of mutations in *HIST2H2AC* has previously been linked to tumorigenesis in cancer[50]. Additionally, Monteirro *et al.* have recently confirmed that indeed *HIST2H2AC* may be a biomarker in BRCA[51]. Since we have already shown a close relationship between edgetic gain perturbation in BRCA and BLCA, we tend to

think that *HIST2H2AC* may also promote tumor proliferation in BLCA. To our knowledge, this study is the first to link *HIST2H2AC* to BLCA oncogenesis.

KICH. To determine if proteins involved in significant edgetic perturbations in KICH play a role in oncogenesis, we searched in PubMed for publications linking these proteins to cancer and specifically to KICH oncogenesis[52]. The top gained biomarker in KICH (gained in 25/25 samples) included a 14-gene signature (*HRK, BCL2, BCL2L1, MCL1, ELAVL1, BCL2A1, GRPR, CEP250, CDK5, DCLK3, DGUOK, SLC12A5* and *NUFIP1*) with the activator of apoptosis harakiri (*HRK)* protein gaining interactions with all the other 13 genes. While *HRK* together with other pro-apoptosis BH3-only members of the Bcl2 family have been extensively linked to apoptosis[53] and possible cancer therapy[54], to our knowledge, no study has linked them directly to KICH oncogenesis. Our study uncovered deregulation of several Bcl2 family members in KICH, and this information may be critical for therapeutic targeting for the only clinically approved drug venetoclax in treating leukaemia[23].

Hierarchical clustering of perturbed edges reveals cancer types sharing similar perturbation signatures

The first set consisted of edgetic perturbations affecting the melanoma-associated antigen 3 protein (*MAGEA3*) and the DNA repair and recombination protein RAD54-like (*RAD54L*) (Fig. S5A). These perturbations were observed only in BRCA, LUAD, STES and BLCA. *Yamada et al*. have shown that deregulation of *MAGEA3* and other cancer testis antigens in BRCA and LUAD maybe a promising route for therapeutic targeting[55]. Our results further suggest that *MAGEA3* is similarly deregulated in STES, LUSC and BLCA and therapeutic targeting of this protein can also be extended to patients diagnosed with these three cancer types. Moreover, the periodic upregulation of *RAD54L* (a DNA repair protein) during the G1/S phase of the cell cycle has been shown to positively correlate with cancer proliferation by setting up feedback loops important in rapid cell multiplication processes[56]. While the TCGA consortium ranks *RAD54L* as one of the genes involved in the DNA repair pathway in some TCGA cancer types[57], our study further suggests that *RAD54L* deregulation may be an indicator of S phase expression in BRCA, LUSC, LUAD, STES and BLCA, therefore implicating *RAD54L* in the proliferation of the above cancer types . The other set of the predicted informative perturbations affected the histone-H3 like centromeric protein A (*CENPA),* kinesin-like protein KIF14 *(KIF14),* RPGR-interacting protein 1 (*RPGRIP1)*, and deoxyribonuclease-2-beta (*DNASE2B)* protein and were observed in KICH, KIRP, KIRC, LIHC, PRAD, STES and THCA (Fig. S5B). While *CENPA* is an epigenetic marker in multiple cancer types indicating how aggressive the cancer type is [58,59], the role of *RPGRIP1* in cancer is not yet clear[60,61]. As it is a player in ciliopathy and proteasome deregulation, our results suggest that additional research should be undertaken to establish the oncogenic or tumorigenic role of *RPGRIP1* in cancer. Additionally, deregulation of *KIF14* and *DNASE2B* via p27 signaling has been observed in multiple cancer types[62,63] and may offer an opportunity for therapeutic targeting since p27 has been found to be prognostic of therapeutic response in cancer[64]. Our results, therefore, indicate that the proteins prone to gaining new interacting partners, while being relatively rare compared to proteins involved in edgetic losses, also have a role in cancer progression, may be important disease monitors and are possible candidates for therapeutic targeting.

Clustering of the edgetic loss patterns identified two main clusters: one cluster consisting of PRAD, STES, THCA, and BLCA and another cluster consisting of KIRP, KIRC, KICH, LUAD, LUSC, LIHC, COAD, BRCA and HNSC (Fig. 7B). Here, we also found two sets of edgetic perturbation patterns important in distinguishing these cancer types. One set contained

edgetic perturbations affecting the peripherin-2 protein (*PRPH*) together with the edges connecting the amyloid-beta precursor protein and the serine/threonine protein kinase NIM1 (*APP-NIM1K*), and the reelin protein with the very low-density lipoprotein receptor (*RELN-VLDLR*) (Fig. S5C). These perturbations were frequently observed in PRAD, STES, THCA, and BLCA, suggesting a shared disease mechanism amongst these cancer types. Depletion of *APP* and *NIM1K* has been found to control the G1 or G2 phase of mitotic cells resulting in abnormal cell sizes[65,66]. The inhibition of proteins involved in the deregulation of G1/G2 checkpoint (e.g., *WEE1*) has been suggested to be a viable option for therapy development (e.g., the drug AZD1775/MK1775) against advanced malignancies[67]. First, our results indicate that the patients having the above perturbations were at an advanced cancer stage, and secondly, AZD1775 may be viable in controlling the G1 or G2 phase of abnormal mitotic cells in patients diagnosed with advanced PRAD, STES, THCA, and BLCA. Wang *et al*. have previously shown that inactivation of alpha-internexins in gastroenteropancreatic neuroendocrine tumors (cancers affecting the pancreas, thyroid glands, gastrointestinal tract and partly the bladder) indicated poor prognosis of the patients[68]. In this study, we specifically found deregulation of *PRPH/peripherin* (an alpha-internexin) via edgetic loss perturbations, thus suggesting that it may be indicative of aggressive gastroenteropancreatic neuroendocrine tumors and consequently provide direction in therapy decision making as well as in disease monitoring. The second set contained high scoring edgetic perturbations affecting the neurotrophic tyrosine kinase receptor type 1 (*NTRK1*) and occurred in KIRP, KIRC, KICH, LUAD, LUSC, LIHC, COAD, BRCA and HNSC (Fig. S5D). As already mentioned above, *NTRK1* is a target for the drug Entrectinib. Our study, therefore, implies that the drug Entrectinib is not only beneficial to non-small cell cancer types but may also be clinically relevant to KIRP, KIRC, KICH, LIHC, COAD, BRCA and HNSC.

Finally, to account for all the molecular pathways affected by the edgetic perturbations, we performed clustering based on both edgetic gains and losses that yielded 3 main groups consisting of (i) LIHC, KICH, KIRC, KIRP, (ii) PRAD, STES, BLCA, THCA and (iii) LUAD, LUSC, COAD, BRCA and HNSC (Fig. 7C). Here, the random forest algorithm predicted 3 groups of perturbed edges as being highly discriminant of the cancer types. The first group of perturbed edges was observed in LIHC, KICH, KIRC, KIRP and affected the Wnt-7b protein (*WNT7B),* together with a number of edges – e.g., the edge between the homeobox protein Hox-B9 and the hepatocyte nuclear factor 3-alpha protein (*HOXB9-FOXA1)* (Fig. S5E). The perturbations affecting *WNT7B* involved edgetic gains in KICH, KIRP and LIHC and edgetic losses in BLCA, BRCA, COAD and STES. No perturbations involving *WNT7B* were found in HNSC, KIRC, LUAD, LUSC, PRAD and THCA. We suggest that *WNT* signaling may be enhanced in KICH, KIRP and LIHC while being depleted in BLCA, BRCA, COAD and STES tumor types. *WNT7B* participates in the deregulation of the beta catenin, c-Jun N-terminal and Ca^{2+} releasing pathways, and its increased expression is critical in cancer development[69]. For example, when up-regulated in BRCA, STES and some types of LIHC (cholangiocarcinoma), this abnormal expression correlates to poor prognosis and can be pharmacologically inhibited in mice[70–73]. Our results indicate that both up- and downregulation of *WNT7B* across cancer types may result in edgetic gains or losses at the protein-protein interaction network, and further suggest which human cancer types may be candidates for a *WNT7B*-based targeted therapy or disease monitoring (i.e, KICH, KIRP, LIHC, BLCA, BRCA, COAD and STES). We also found another grouping of multiple perturbed edges that were crucial in distinguishing the cancer types (Fig. S5F). Of these, the edge between the phosphatase and tensin homolog protein and sialyltransferase 8F protein (*PTEN-ST8SIA6*) was predicted to have the highest score. This edgetic perturbation involved edgetic losses in BRCA, COAD, HNSC, KIRC, KIRP, LIHC, LUAD and LUSC, with an edgetic gain in PRAD, but no perturbations in THCA,

BLCA and STES. Our study agrees with the current knowledge on the loss of the tumor suppressor *PTEN,* in multiple cancer types[74]. The loss of *PTEN* in cancer has been correlated to immunosuppression and reduced T cell trafficking in mice melanoma cells[75]. Our findings suggest that *PTEN* and P13-AKT pathway targeted immunotherapy may be beneficial in BRCA, COAD, HNSC, KIRC, KIRP, LIHC, LUAD and LUSC cancer types but probably not in THCA, BLCA and STES. Lastly, edgetic perturbations affecting the ciliary neurotrophic factor (*CNTF*), otoferlin (*OTOF*), and the inhibitor of CDK interacting with cyclin A1 (*INCA1*) proteins together with the edge between cytokeratin-75 and cullin-3 (*KRT75-CUL3*) proteins were also predicted to be crucial in distinguishing the cancer types (Fig. S5G). *CNTF* and *INCA1* perturbations were only observed in cluster 3 cancer types and involved edgetic losses in all the 5 cancer types in cluster 3. *OTOF* perturbations affected edgetic losses in COAD, HNSC, LUSC and BRCA, edgetic gains in BLCA, KIRP and THCA, but no perturbations in KIRC, LIHC, PRAD and STES. Our findings confirm that the above mentioned proteins are crucial in tumor progression as previously suggested[76–78] and pinpoint their important role in tumor progression in BRCA, LUAD, LUSC, COAD, BRCA and HNSC.

Isoform switches and resultant domain changes between cancer and healthy states result in edgetic perturbations.

To test whether our results were brought about by differential gene expression or were due to domain changes between the healthy and cancer state, we used the R package BiRewire first to generate a randomized network and then analyzed the resulting perturbations. We did this by building condition-specific PPINs in three randomly selected cancer types (BRCA, THCA and BLCA). We were able to (i) recover the prominent proteins involved in edgetic perturbations resulting from the loss or gain of genes in the cancer state (see Table S4a and Dataset 2), and (ii) found that both BRCA and THCA had similar perturbation patterns even after network randomization (see Fig. S1). For BLCA, the results were different: the cancer PPIN was significantly bigger than the healthy network when using the randomized network, suggesting that our approach may not be optimal when dealing with few paired data sets (19 paired samples in the case of BLCA). These results further indicate that the edgetic perturbations we obtained were indeed a property of the protein interactions and also the expression landscape of the genes. In brief, the cancer state expressed slightly fewer genes than the healthy state, resulting in a reduced number of protein products available to interact with each other. The consequence of this is manifested in the PPIN, where a reduced (or increased) number of interactions is observed. In THCA, for example, the mean number of interactions in the cancer state was 115673, while the mean in the healthy state was 108334 (Fig. S1). For the obtained perturbations, the proteins involved in these disruptions were still predictive of patient survival (Fig. S4).

Protein nodes rewired across cancer types are involved in tumorigenesis

We used DyNet algorithm in Cytoscape to find significantly rewired proteins, that is, nodes recurrently affected by edgetic perturbations. We selected a node as considerably rewired if it had a DyNet score of at least 0.5 (see methodology section)[1]. In cancer, significantly rewired nodes were either perturbed across cancer types or were specific to a cancer type, with some being known cancer biomarkers (Dataset 3). Nodes rewired across multiple cancers, for example, Q9HBJ0 (*PLAC1*), Q9BVV2 (*FNDC11*) and Q5T7N2 (*L1TD1*) have been suggested to influence the growth of tumor cells in various cancer types[2–3]. Furthermore, to better understand the association between the significantly rewired nodes and cancer, we used DisGeNET[4] in clusterProfiler to search for any gene-disease relationships associated with the rewired nodes. We found that most of these proteins were significantly ($p < 0.05$) associated

with diseases observed during the onset of cancer (e.g bronchial and lung dysplasia) as well as the development of multiple cancer types (Table S6).

References

1. Goenawan, I. H., Bryan, K. & Lynn, D. J. DyNet: visualization and analysis of dynamic molecular interaction networks. *Bioinformatics* **32**, 2713–2715 (2016).
2. Fant, M., Farina, A., Nagaraja, R. & Schlessinger, D. PLAC1 (Placenta-specific 1): A novel, X-linked gene with roles in reproductive and cancer biology. *Prenat Diagn* **30**, 497–502 (2010).
3. Närvä, E. *et al.* RNA-Binding Protein L1TD1 Interacts with LIN28 via RNA and is Required for Human Embryonic Stem Cell Self-Renewal and Cancer Cell Proliferation. *Stem Cells* **30**, 452–460 (2012).
4. Piñero, J. *et al.* DisGeNET: a comprehensive platform integrating information on human disease-associated genes and variants. *Nucleic Acids Res* **45**, D833–D839 (2017).
5. Futreal, P. A. *et al.* A census of human cancer genes. *Nature Reviews Cancer* **4**, 177–183 (2004).
6. Bailey, M. H. *et al.* Comprehensive Characterization of Cancer Driver Genes and Mutations. *Cell* **173**, 371-385.e18 (2018).
7. Zou, K. H., Fielding, J. R., Silverman, S. G. & Tempany, C. M. C. Hypothesis Testing I: Proportions. *Radiology* **226**, 609–613 (2003).
8. Merid, S. K., Goranskaya, D. & Alexeyenko, A. Distinguishing between driver and passenger mutations in individual cancer genomes by network enrichment analysis. *BMC Bioinformatics* **15**, 308 (2014).
9. Pon, J. R. & Marra, M. A. Driver and passenger mutations in cancer. *Annu Rev Pathol* **10**, 25–50 (2015).
10. Tokheim, C. J., Papadopoulos, N., Kinzler, K. W., Vogelstein, B. & Karchin, R. Evaluating the evaluation of cancer driver genes. *Proc. Natl. Acad. Sci. U.S.A.* **113**, 14330–14335 (2016).
11. Leiserson, M. D. M. *et al.* Pan-cancer network analysis identifies combinations of rare somatic mutations across pathways and protein complexes. *Nat. Genet.* **47**, 106–114 (2015).
12. Chen, Y., Zhang, L. & Jones, K. A. SKIP counteracts p53-mediated apoptosis via selective regulation of p21Cip1 mRNA splicing. *Genes Dev.* **25**, 701–716 (2011).
13. Chan, K.-M. *et al.* The histone H3.3K27M mutation in pediatric glioma reprograms H3K27 methylation and gene expression. *Genes Dev.* **27**, 985–990 (2013).
14. Liu, G. *et al.* High SKIP expression is correlated with poor prognosis and cell proliferation of hepatocellular carcinoma. *Med. Oncol.* **30**, 537 (2013).
15. Ramakrishnan, S., Ellis, L. & Pili, R. Histone modifications: implications in renal cell carcinoma. *Epigenomics* **5**, 453–462 (2013).
16. Wang, X. *et al.* Clinical and prognostic relevance of EZH2 in breast cancer: A meta-analysis. *Biomed. Pharmacother.* **75**, 218–225 (2015).
17. Wang, Y. *et al.* Overexpression of YB1 and EZH2 are associated with cancer metastasis and poor prognosis in renal cell carcinomas. *Tumour Biol.* **36**, 7159–7166 (2015).
18. Yan, K.-S. *et al.* EZH2 in Cancer Progression and Potential Application in Cancer Therapy: A Friend or Foe? *Int J Mol Sci* **18**, (2017).
19. Poornima, P., Kumar, J. D., Zhao, Q., Blunder, M. & Efferth, T. Network pharmacology of cancer: From understanding of complex interactomes to the design of multi-target specific therapeutics from nature. *Pharmacological Research* **111**, 290–302 (2016).

20. Engin, H. B., Kreisberg, J. F. & Carter, H. Structure-Based Analysis Reveals Cancer Missense Mutations Target Protein Interaction Interfaces. *PLoS ONE* **11**, e0152929 (2016).
21. Jubb, H. C. *et al.* Mutations at protein-protein interfaces: Small changes over big surfaces have large impacts on human health. *Prog. Biophys. Mol. Biol.* **128**, 3–13 (2017).
22. Cui, H., Zhao, N. & Korkin, D. Multilayer View of Pathogenic SNVs in Human Interactome through in-silico Edgetic Profiling. *Journal of Molecular Biology* (2018) doi:10.1016/j.jmb.2018.07.012.
23. Green, D. R. A BH3 Mimetic for Killing Cancer Cells. *Cell* **165**, 1560 (2016).
24. Nilsson, G. & Kannius-Janson, M. Forkhead Box F1 promotes breast cancer cell migration by upregulating lysyl oxidase and suppressing Smad2/3 signaling. *BMC Cancer* **16**, (2016).
25. Fahey, J. M. & Girotti, A. W. NITRIC OXIDE-MEDIATED RESISTANCE TO PHOTODYNAMIC THERAPY IN A HUMAN BREAST TUMOR XENOGRAFT MODEL: IMPROVED OUTCOME WITH NOS2 INHIBITORS. *Nitric Oxide* **62**, 52–61 (2017).
26. Mao, X. *et al.* The heparan sulfate sulfotransferase 3-OST3A (HS3ST3A) is a novel tumor regulator and a prognostic marker in breast cancer. *Oncogene* **35**, 5043–5055 (2016).
27. Tian, H., Li, L., Liu, X.-X. & Zhang, Y. Antitumor Effect of Antisense Ornithine Decarboxylase Adenovirus on Human Lung Cancer Cells. *Acta Biochimica et Biophysica Sinica* **38**, 410–416 (2006).
28. Kumar, G. *et al.* Simultaneous targeting of 5-LOX-COX and ODC block NNK-induced lung adenoma progression to adenocarcinoma in A/J mice. *Am J Cancer Res* **6**, 894–909 (2016).
29. Huang, R. S., Duan, S., Kistner, E. O., Hartford, C. M. & Dolan, M. E. Genetic variants associated with carboplatin-induced cytotoxicity in cell lines derived from Africans. *Mol Cancer Ther* **7**, 3038–3046 (2008).
30. Liu, P. *et al.* Identification of somatic mutations in non-small cell lung carcinomas using whole-exome sequencing. *Carcinogenesis* **33**, 1270–1276 (2012).
31. Green, W. J. *et al.* KI67 and DLX2 predict increased risk of metastasis formation in prostate cancer-a targeted molecular approach., KI67 and DLX2 predict increased risk of metastasis formation in prostate cancer–a targeted molecular approach. *Br J Cancer* **115, 115**, 236, 236–242 (2016).
32. Fujita, K. *et al.* Cancer Therapy Due to Apoptosis: Galectin-9., Cancer Therapy Due to Apoptosis: Galectin-9. *Int J Mol Sci* **18, 18**, (2017).
33. Sturm, I. *et al.* Loss of the tissue-specific proapoptotic BH3-only protein Nbk/Bik is a unifying feature of renal cell carcinoma. *Cell Death and Differentiation* **13**, 619–627 (2006).
34. Jiang, Z. *et al.* Oncofetal protein IMP3. *Cancer* **112**, 2676–2682 (2008).
35. Anasa, V. V., Ravanan, P. & Talwar, P. Multifaceted roles of ASB proteins and its pathological significance. *Front. Biol.* (2018) doi:10.1007/s11515-018-1506-2.
36. Prestin, K. *et al.* Modulation of expression of the nuclear receptor NR0B2 (small heterodimer partner 1) and its impact on proliferation of renal carcinoma cells. *Onco Targets Ther* **9**, 4867–4878 (2016).
37. Kim, C. H., Neiswender, H., Baik, E. J., Xiong, W. C. & Mei, L. Beta-catenin interacts with MyoD and regulates its transcription activity. *Mol Cell Biol* **28**, 2941–2951 (2008).
38. Kagey, M. H. & He, X. Rationale for targeting the Wnt signalling modulator Dickkopf-1 for oncology., Rationale for targeting the Wnt signalling modulator Dickkopf-1 for oncology. *Br J Pharmacol* **174, 174**, 4637, 4637–4650 (2017).
39. Hirata, H. *et al.* Wnt antagonist DKK1 acts as a tumor suppressor gene that induces apoptosis and inhibits proliferation in human renal cell carcinoma. *Int J Cancer* **128**, 1793–1803 (2011).

40. Méniel, V. *et al.* Cited1 deficiency suppresses intestinal tumorigenesis., Cited1 Deficiency Suppresses Intestinal Tumorigenesis. *PLoS Genet* **9, 9**, e1003638–e1003638 (2013).
41. Zheng, Y., Zhou, J. & Tong, Y. Gene signatures of drug resistance predict patient survival in colorectal cancer., Gene signatures of drug resistance predict patient survival in colorectal cancer. *Pharmacogenomics J* **15, 15**, 135, 135–143 (2015).
42. Dart, A. E. *et al.* PAK4 promotes kinase-independent stabilization of RhoU to modulate cell adhesion. *J Cell Biol* **211**, 863–879 (2015).
43. Liggins, A. P., Lim, S. H., Soilleux, E. J., Pulford, K. & Banham, A. H. A panel of cancer-testis genes exhibiting broad-spectrum expression in haematological malignancies., A panel of cancer-testis genes exhibiting broad-spectrum expression in haematological malignancies. *Cancer Immun* **10, 10**, 8–8 (2010).
44. Freitas, M. *et al.* Expression of cancer/testis antigens is correlated with improved survival in glioblastoma. *Oncotarget* **4**, 636–646 (2013).
45. Yamauchi, K. *et al.* Disease-causing mutant WNK4 increases paracellular chloride permeability and phosphorylates claudins. *Proc Natl Acad Sci U S A* **101**, 4690–4694 (2004).
46. Singh, A. B., Sharma, A. & Dhawan, P. Claudin family of proteins and cancer: an overview., Claudin Family of Proteins and Cancer: An Overview. *J Oncol* **2010, 2010**, 541957–541957 (2010).
47. Namani, A., Matiur, M. R., Chen, M. & Tang, X. Gene-expression signature regulated by the KEAP1-NRF2-CUL3 axis is associated with a poor prognosis in head and neck squamous cell cancer., Gene-expression signature regulated by the KEAP1-NRF2-CUL3 axis is associated with a poor prognosis in head and neck squamous cell cancer. *BMC Cancer* **18, 18**, 46–46 (2018).
48. Sharma, P. C. & Verma, R. Implication of HSP70 in the Pathogenesis of Gastric Cancer. in *HSP70 in Human Diseases and Disorders* (eds. Asea, A. A. A. & Kaur, P.) vol. 14 113–130 (Springer International Publishing, 2018).
49. Yang, Y. *et al.* MicroRNA-195 acts as a tumor suppressor by directly targeting Wnt3a in HepG2 hepatocellular carcinoma cells. *Molecular Medicine Reports* **10**, 2643–2648 (2014).
50. He, Q. *et al.* Genome-wide prediction of cancer driver genes based on SNP and cancer SNV data. *Am J Cancer Res* **4**, 394–410 (2014).
51. Monteiro, F. L. *et al.* The histone H2A isoform Hist2h2ac is a novel regulator of proliferation and epithelial-mesenchymal transition in mammary epithelial and in breast cancer cells. *Cancer Lett.* **396**, 42–52 (2017).
52. Jurca, G. *et al.* Integrating text mining, data mining, and network analysis for identifying genetic breast cancer trends. *BMC Res Notes* **9**, 236 (2016).
53. Chang, I. *et al.* Hrk mediates 2-methoxyestradiol-induced mitochondrial apoptotic signaling in prostate cancer cells. *Mol. Cancer Ther.* **12**, 1049–1059 (2013).
54. Adams, J. M. & Cory, S. The BCL-2 arbiters of apoptosis and their growing role as cancer targets. *Cell Death Differ.* **25**, 27–36 (2018).
55. Yamada, R. *et al.* Preferential expression of cancer/testis genes in cancer stem-like cells: proposal of a novel sub-category, cancer/testis/stem gene. *Tissue Antigens* **81**, 428–434 (2013).
56. Mjelle, R. *et al.* Cell cycle regulation of human DNA repair and chromatin remodeling genes. *DNA Repair* **30**, 53–67 (2015).
57. Knijnenburg, T. A. *et al.* Genomic and Molecular Landscape of DNA Damage Repair Deficiency across The Cancer Genome Atlas. *Cell Rep* **23**, 239-254.e6 (2018).
58. Arimura, Y. *et al.* Crystal structure and stable property of the cancer-associated heterotypic nucleosome containing CENP-A and H3.3. *Sci Rep* **4**, (2014).

59. Athwal, R. K. *et al.* CENP-A nucleosomes localize to transcription factor hotspots and subtelomeric sites in human cancer cells. *Epigenetics & Chromatin* **8**, 2 (2015).
60. Coene, K. L. M. *et al.* The ciliopathy-associated protein homologs RPGRIP1 and RPGRIP1L are linked to cilium integrity through interaction with Nek4 serine/threonine kinase. *Hum Mol Genet* **20**, 3592–3605 (2011).
61. Gerhardt, C., Leu, T., Lier, J. M. & Rüther, U. The cilia-regulated proteasome and its role in the development of ciliopathies and cancer. *Cilia* **5**, (2016).
62. Xu, H. *et al.* Silencing of KIF14 interferes with cell cycle progression and cytokinesis by blocking the p27(Kip1) ubiquitination pathway in hepatocellular carcinoma. *Exp. Mol. Med.* **46**, e97 (2014).
63. Siddam, A. D. *et al.* The RNA-binding protein Celf1 post-transcriptionally regulates p27Kip1 and Dnase2b to control fiber cell nuclear degradation in lens development. *PLOS Genetics* **14**, e1007278 (2018).
64. Chu, I. M., Hengst, L. & Slingerland, J. M. The Cdk inhibitor p27 in human cancer: prognostic potential and relevance to anticancer therapy. *Nature Reviews Cancer* **8**, 253–267 (2008).
65. Wu, L. & Russell, P. Nim1 kinase promotes mitosis by inactivating Wee1 tyrosine kinase. *Nature* **363**, 738–741 (1993).
66. Sobol, A., Galluzzo, P., Weber, M. J., Alani, S. & Bocchetta, M. Depletion of Amyloid Precursor Protein (APP) causes G0 arrest in non-small cell lung cancer (NSCLC) cells. *J. Cell. Physiol.* **230**, 1332–1341 (2015).
67. Yin, Y. *et al.* Wee1 inhibition can suppress tumor proliferation and sensitize p53 mutant colonic cancer cells to the anticancer effect of irinotecan. *Molecular Medicine Reports* **17**, 3344–3349 (2018).
68. Wang, Y. *et al.* Loss of expression and prognosis value of alpha-internexin in gastroenteropancreatic neuroendocrine neoplasm. *BMC Cancer* **18**, 691 (2018).
69. Kirikoshi, H. & Katoh, M. Expression of WNT7A in human normal tissues and cancer, and regulation of WNT7A and WNT7B in human cancer. *Int. J. Oncol.* **21**, 895–900 (2002).
70. Chen, J. *et al.* Up-regulation of Wnt7b rather than Wnt1, Wnt7a, and Wnt9a indicates poor prognosis in breast cancer. 11.
71. Souza-Santos, P. T. de *et al.* Mutations, Differential Gene Expression, and Chimeric Transcripts in Esophageal Squamous Cell Carcinoma Show High Heterogeneity. *Transl Oncol* **11**, 1283–1291 (2018).
72. Guest, R. V., Boulter, L., Dwyer, B. J. & Forbes, S. J. Understanding liver regeneration to bring new insights to the mechanisms driving cholangiocarcinoma. *npj Regenerative Medicine* **2**, 13 (2017).
73. Boulter, L. *et al.* WNT signaling drives cholangiocarcinoma growth and can be pharmacologically inhibited. *J Clin Invest* **125**, 1269–1285 (2015).
74. Dillon, L. M. & Miller, T. W. Therapeutic targeting of cancers with loss of PTEN function. *Curr Drug Targets* **15**, 65–79 (2014).
75. Peng, W. *et al.* Loss of PTEN promotes resistance to T cell-mediated immunotherapy. *Cancer Discov* **6**, 202–216 (2016).
76. Bäumer, N. *et al.* Inhibitor of Cyclin-dependent Kinase (CDK) Interacting with Cyclin A1 (INCA1) Regulates Proliferation and Is Repressed by Oncogenic Signaling. *J Biol Chem* **286**, 28210–28222 (2011).
77. Zenner, H. P., Pfister, M., Friese, N., Zrenner, E. & Röcken, M. Molekulare personalisierte Medizin. *HNO* **62**, 520–524 (2014).
78. Galoczova, M., Coates, P. & Vojtesek, B. STAT3, stem cells, cancer stem cells and p63. *Cell. Mol. Biol. Lett.* **23**, 12 (2018)

Supporting Information Legends

Text S1.docx: Supplementary text with additional data regarding the methodology and results sections.

Table S1.xlsx: An isoform switch coupled with domain changes in any of the interacting proteins results to edgetic perturbations.

Table S2.xlsx: The identified edgetic perturbations are retained in the protein-abundance filtered PPIN.

Table S3.xlsx: Specific cancer SMGs are involved in edgetic perturbations of cancer PPINs.

Table S4.xlsx: Importance of the proteins involved in multiple edgetic perturbations or edges frequently perturbed across patients of a cancer type and their significance in predicting overall patient survival.

Table S5.xlsx: Characteristics of healthy and cancer PPINs and associated perturbations in 13 cancer types.

Table S6.xlsx: Enriched gene ontologies, disease ontologies and KEGG pathways for proteins involved in egdetic perturbations.

Table S7.xlsx: Multi-cancer edgetic perturbations

Table S8.xlsx: Distribution of patient samples harboring the top gained/lost edges across cancer stages.

Table S9.xlsx: Clinical and phenotypic traits of 639 patients diagnosed with 13 cancer types as obtained from TCGA.

Fig. S1.pdf: Differences between the number of interactions in the healthy and cancer states for BLCA and BRCA. Healthy and cancer PPINs significantly differ in size in even in the condition specific networks obtained from the randomized PPIN (p-value <0.05). The density plots indicate the distribution of paired cancer and healthy PPIN sizes in BRCA and BLCA. For both cancer types, the healthy PPIN was larger than the corresponding cancer PPIN.

Fig. S2.pdf: Kaplan-Meier survival analysis plots of multigene cancer biomarkers involved in edgetic perturbations. The x axes indicate the number of days until patient death whereas the y axes indicate the probability of patient survival. In all the figures, the green lines indicate better survival (longer life-span) after cancer diagnosis while the red lines indicate poor survival (shorter life-span) after cancer diagnosis as a result of the proteins involved in edgetic gains or losses. In all the cases, the proteins involved in edgteic perturbations predicted poor survival of the patients (Logrank test p-value < 0.05), indicating their importance in cancer monitoring and prognosis. (i) Overall survival predicted from gene signatures involved in edgetic gains across most patients of a cancer type (except for LIHC), (ii) Overall survival predicted from gene signatures involved in edgetic losses across most patients of a cancer type, (iii) Overall survival predicted from gene signatures involved in edgetic gains across patients showing cancer-specific perturbations, (iv) Overall survival predicted from gene signatures involved in edgetic losses across patients showing cancer-specific perturbations. The names of the prominent proteins with multiple perturbations responsible for the above observations can be found in S4 Table.

Fig. S3.pdf: Kaplan-Meier survival analysis plots of multigene cancer biomarkers involved in edgetic perturbations as a result of Isoform/domain changes in the cancer state. The x axes indicate the number of days until patient death whereas the y axes indicate the probability of patient survival. In all the figures, the green lines indicate better survival (longer life-span) after cancer diagnosis while the red lines indicate poor survival (shorter life-span) after cancer diagnosis as a result of the proteins involved in edgetic gains or losses. In all the cases, the proteins involved in edgteic perturbations predicted poor survival of the patients (Logrank test

p-value < 0.05), indicating their importance in cancer monitoring and prognosis. (i) Overall survival predicted from gene signatures involved in edgetic gains across most patients of a cancer type, (ii) Overall survival predicted from gene signatures involved in edgetic losses across most patients of a cancer type. The names of the prominent proteins with multiple perturbations responsible for the above observations can be found in S4 Table (marked with ***).

Fig. S4.pdf: Kaplan-Meier survival analysis plots of multigene cancer biomarkers involved in edgetic perturbations from the randomized PPIN. The x axes indicate the number of days until patient death whereas the y axes indicate the probability of patient survival. In all the figures, the green lines indicate better survival (longer life-span) after cancer diagnosis while the red lines indicate poor survival (shorter life-span) after cancer diagnosis as a result of the proteins involved in edgetic gains or losses. In all the cases, the proteins involved in edgteic perturbations predicted poor survival of the patients (Logrank test p-value < 0.05), indicating their importance in cancer monitoring and prognosis. (A) Overall survival predicted from gene signatures involved in edgetic gains across most patients in BRCA, (B) Overall survival predicted from gene signatures involved in edgetic gains across most patients in BLCA. The names of the prominent proteins with multiple perturbations responsible for the above observations can be found in S4 Table.

Fig. S5.pdf: Top ranked features (edges) from the Random Forest algorithm that distinguish cancer types based on the identified groups from hierarchical clustering (Fig. 7 of main text). The x axes indicate the percentage (%) Mean Squared Error (MSE2). The higher the %MSE of the feature (perturbed edge), the more important the perturbed edge is in identifying a cluster.

Fig. S6.pdf: For each plot, the left blue curve represents the lowly-expressed genes while the grey curve represents the highly-expressed genes across patients of a cancer type for both healthy and cancer samples, respectively. We used these characteristic peaks as a threshold and only kept the genes with an all-samples probability score of greater than 0.8 for subsequent analysis.

Datasets available at EdgeExplorer: http://webclu.bio.wzw.tum.de/EdgeExplorer/AboutOurSite

Dataset 1.xls: Comparison of the expressed genes between cancer and healthy states across 13 cancer types.

Dataset 2.xlsx: Edgetic perturbations in BLCA and BRCA after network randomization.

Dataset 3.xlsx.zip: Edgetic perturbations for each cancer type.

Dataset 4.xlsx.zip: Reproducible edgetic perturbations for each cancer type.

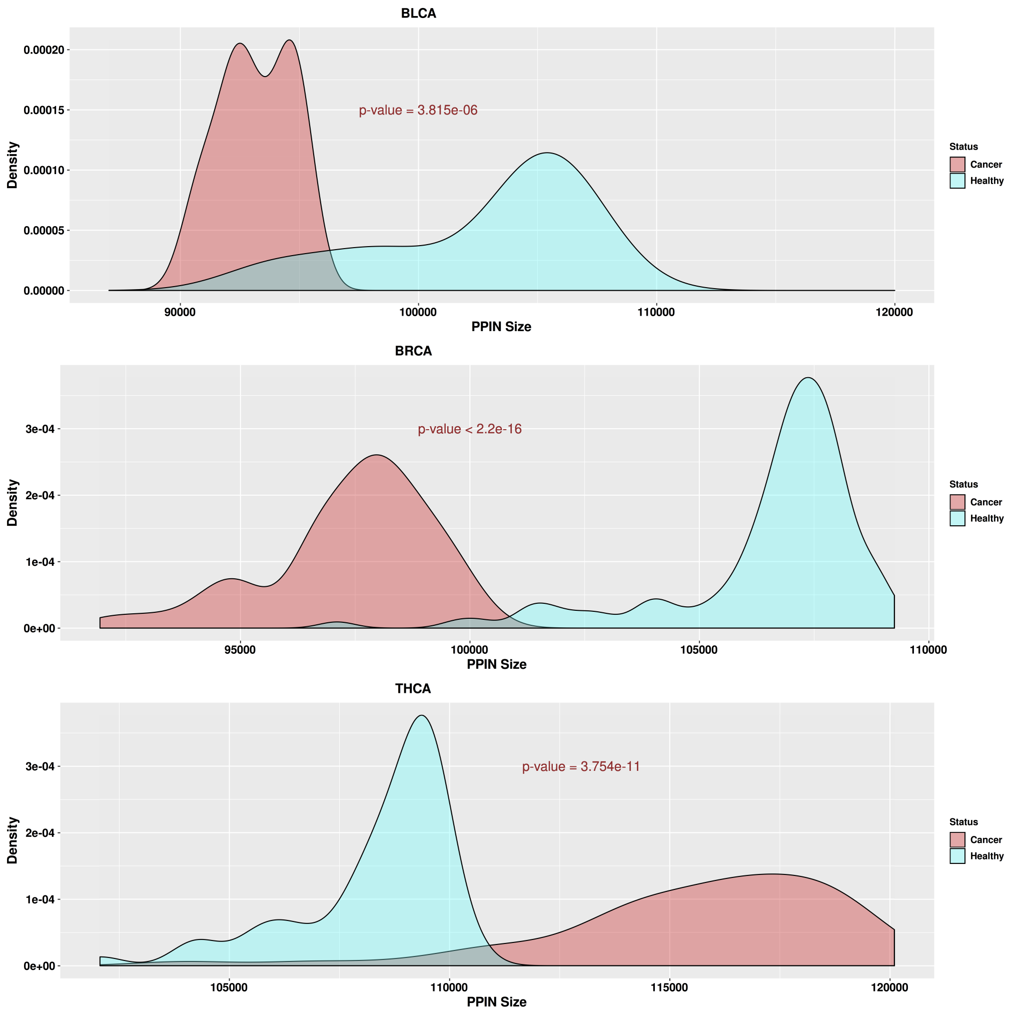

S1 Fig: Differences between the number of interactions in the healthy and cancer statesfor BLCA, BRCA and THCA. Healthy and cancer PPINs significantly differ in size even in the condition specific networks obtained from the randomised PPIN (p-value <0.05).The density plots indicate the distribution of paired cancer and healthy PPIN sizes in BLCA, BRCA and THCA. For BRCA and BLCA, the healthy PPIN was larger than the corresponding cancer PPIN while for THCA, the cancer PPIN was larger than the corresponding healthy PPIN.

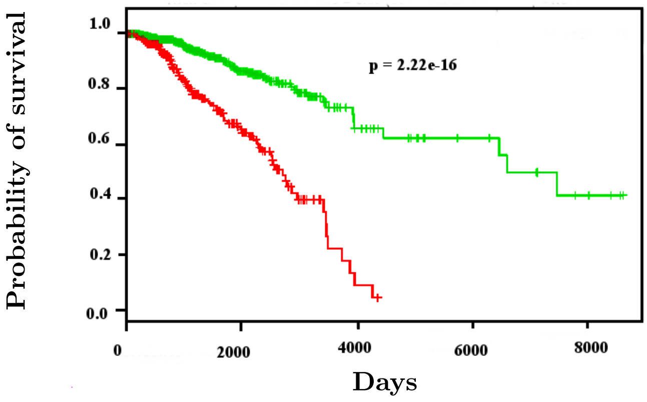

Figure Ai: BRCA Top Gain

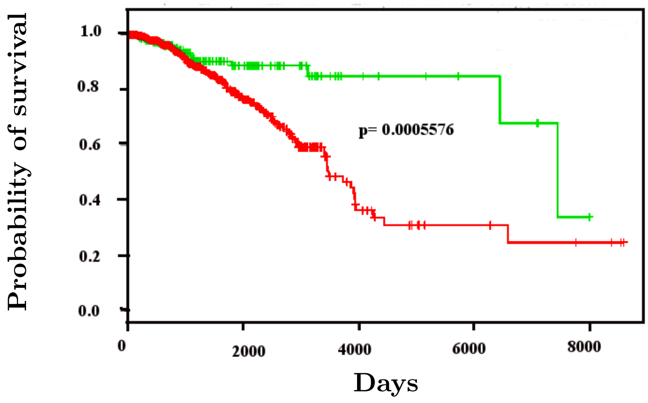

Figure Aii: BRCA Specific Top Gain

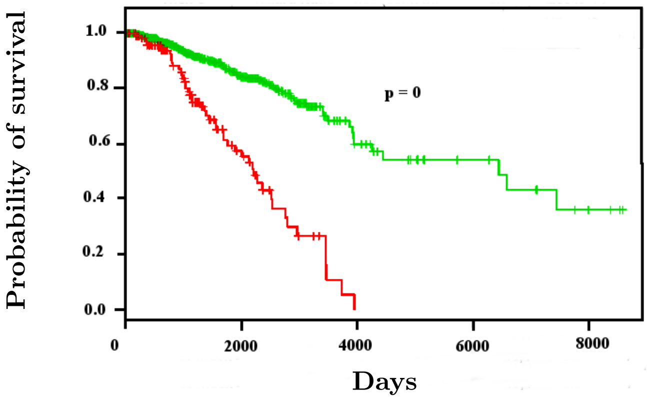

Figure Aiii: BRCA Top Lost

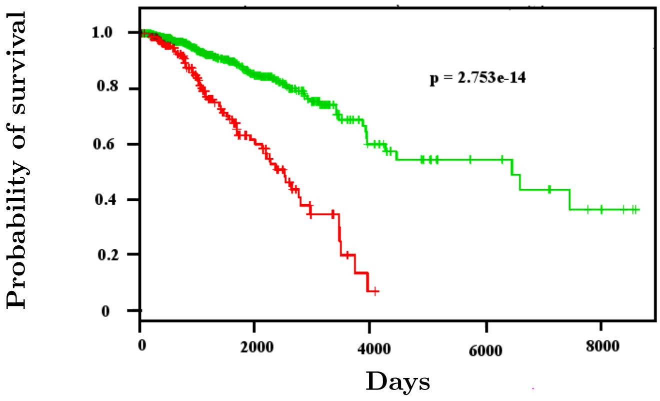

Figure Aiv: BRCA Specific Top Lost

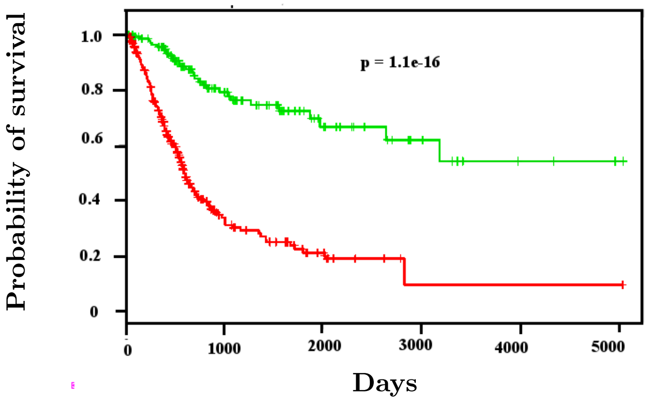

Figure Bi: BLCA Top Gain

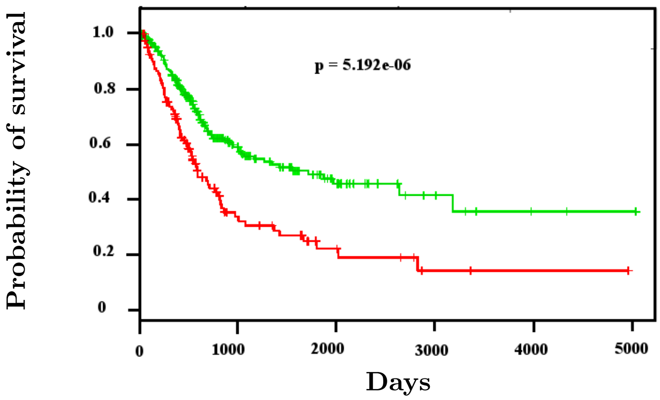

Figure Bii: BLCA Specific Top Gain

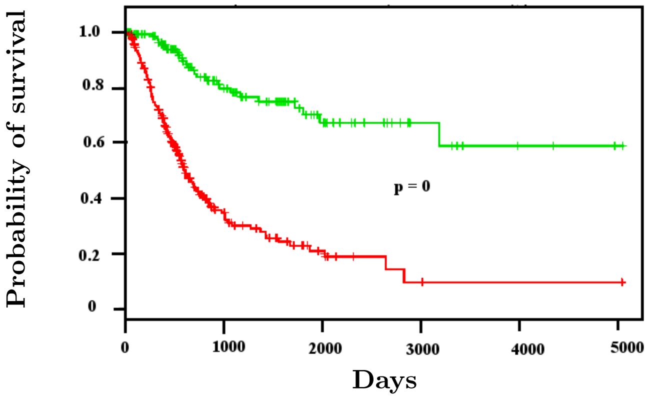

Figure Biii: BLCA Top Lost

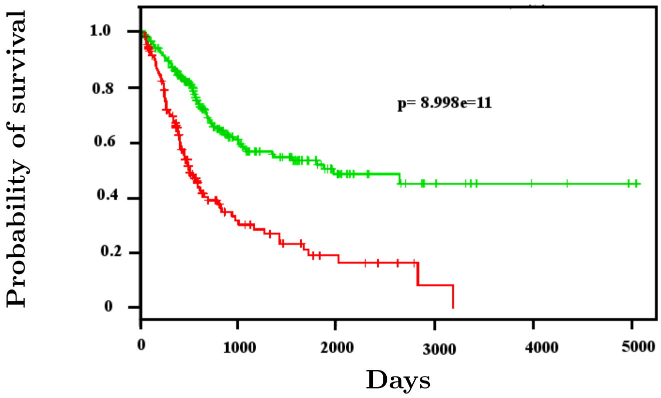

Figure Biv: BLCA Specific Top Lost

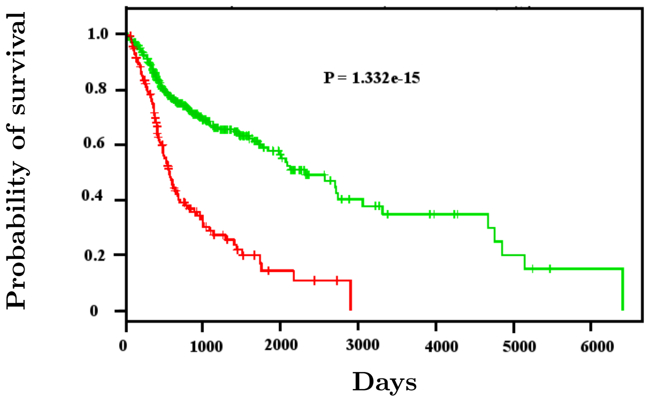

Figure Ci: HNSC Top Gain

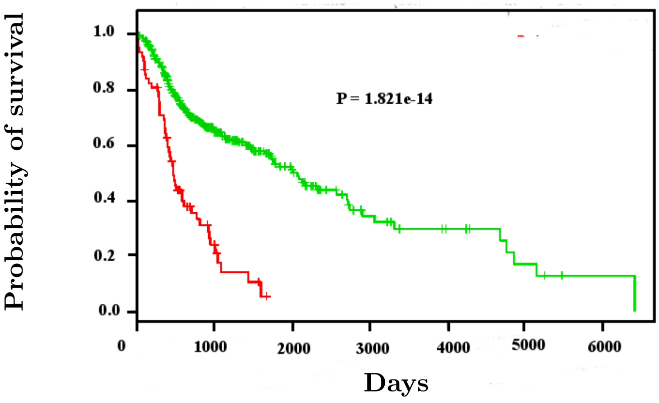

Figure Cii: HNSC Specific Top Gain

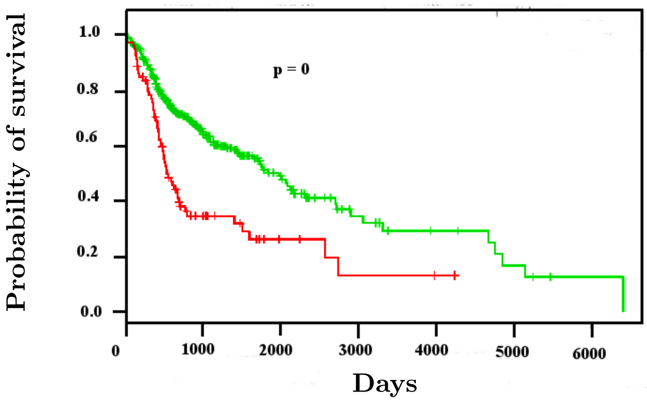

Figure Ciii: HNSC Top Lost

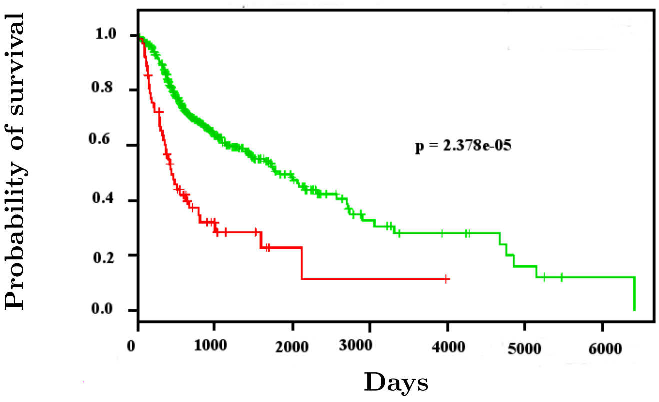

Figure Civ: HNSC Specific Top Lost

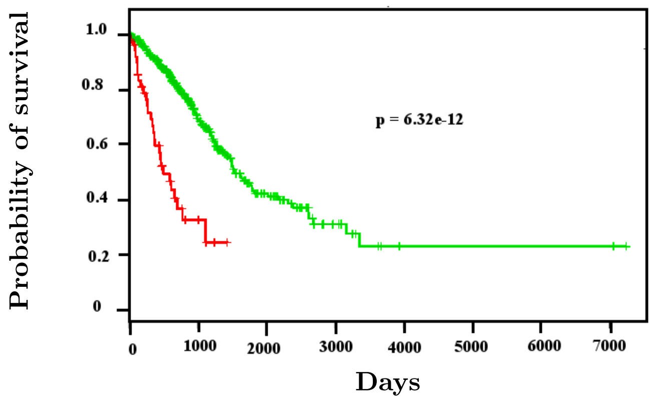

Figure Di: LUAD Top Gain

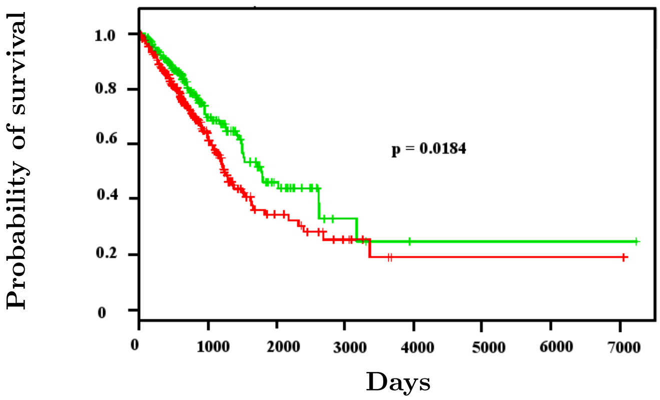

Figure Dii: LUAD Specific Top Gain

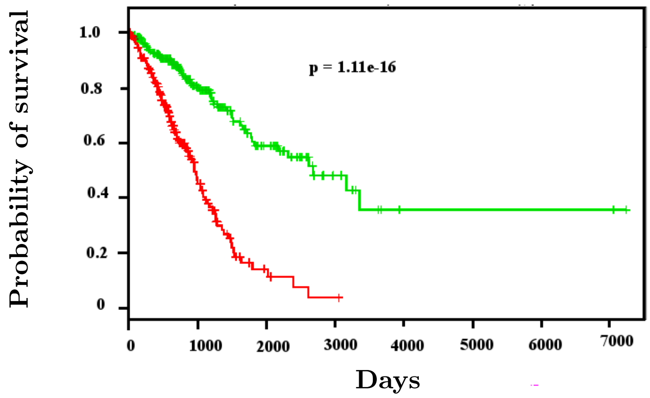

Figure Diii: LUAD Top Lost

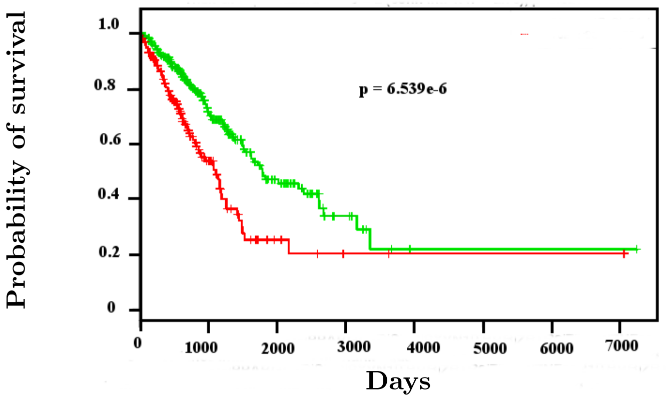

Figure Div: LUAD Specific Top Lost

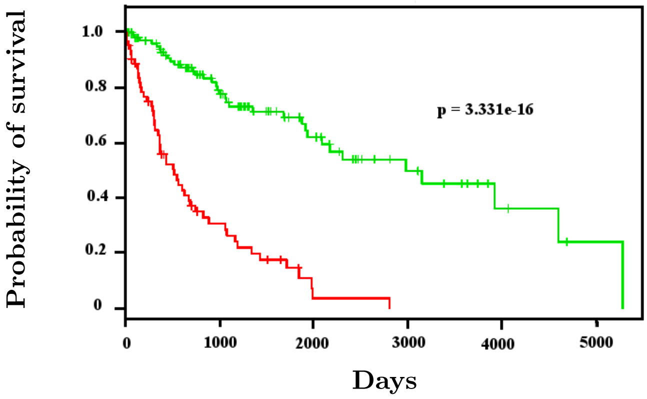

Figure Ei: LUSC Top Gain

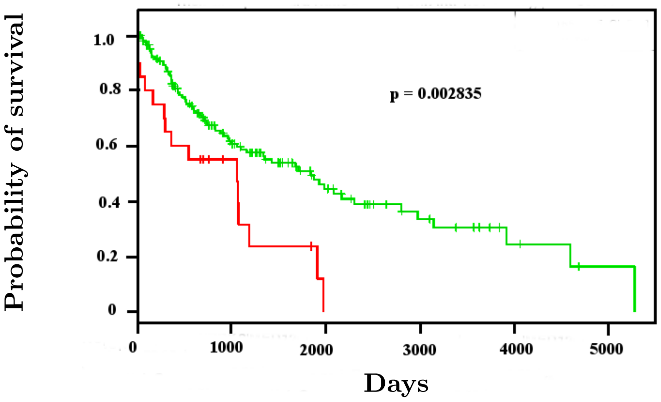

Figure Eii: LUSC Specific Top Gain

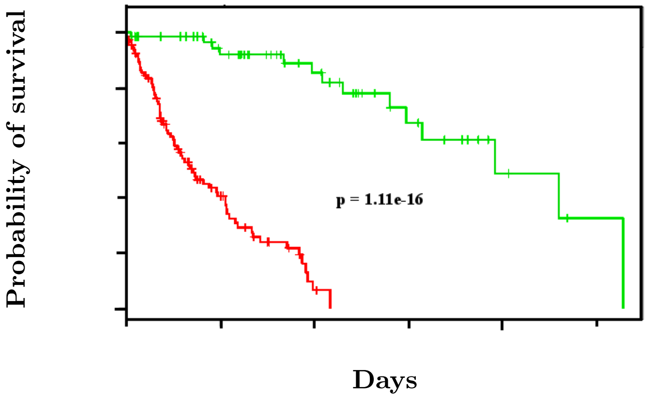

Figure Eiii: LUSC Top Lost

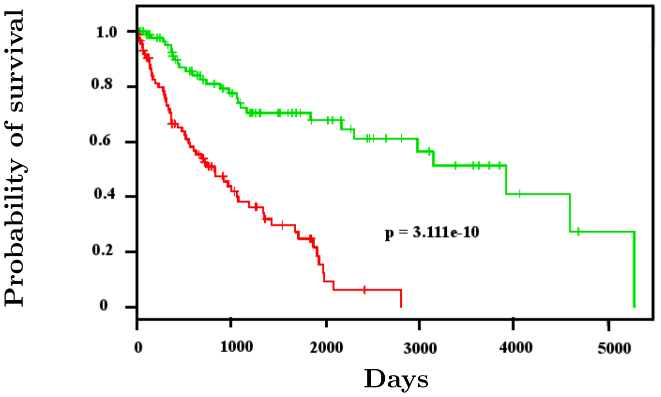

Figure Eiv: LUSC Specific Top Lost

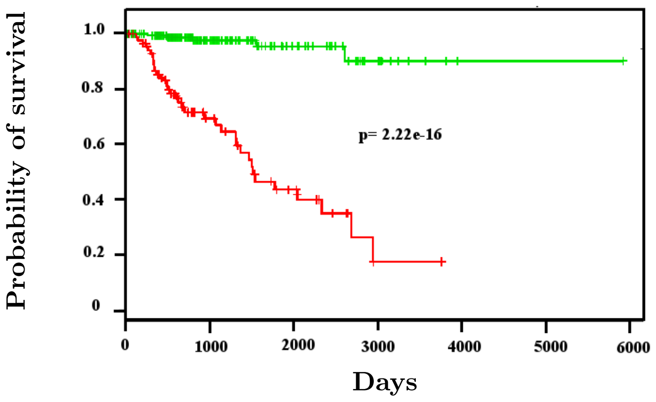

Figure Fi: KIRP Top Gain

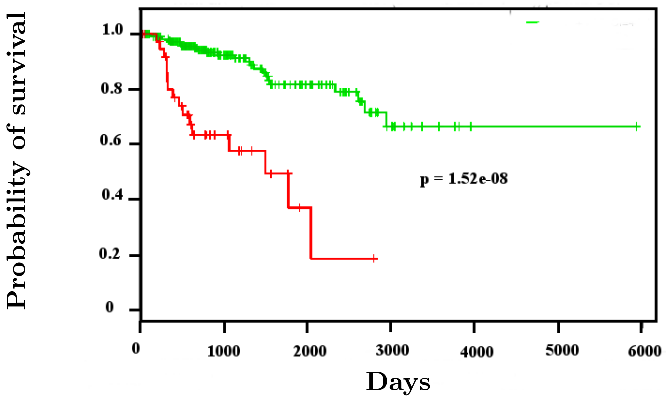

Figure Fii: KIRP Specific Top Gain

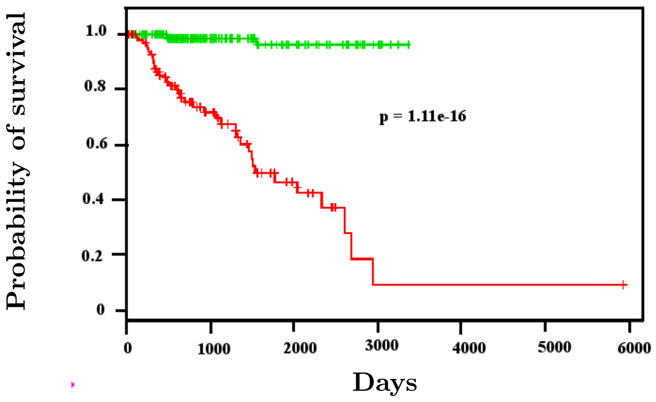

Figure Fiii: KIRP Top Lost

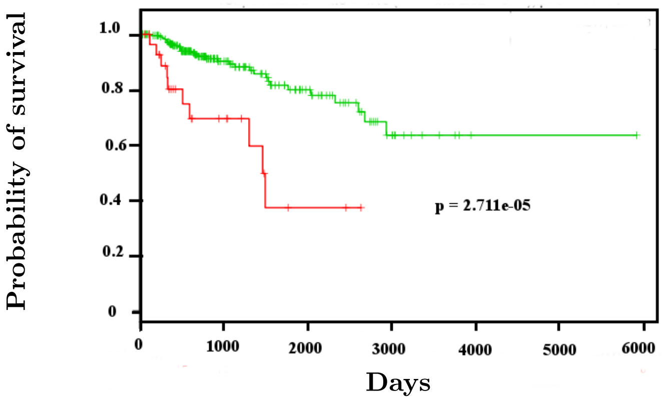

Figure Fiv: KIRP Specific Top Lost

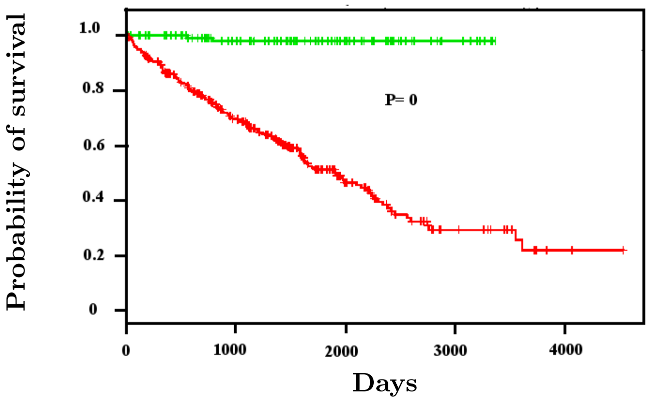

Figure Gi: KIRC Top Gain

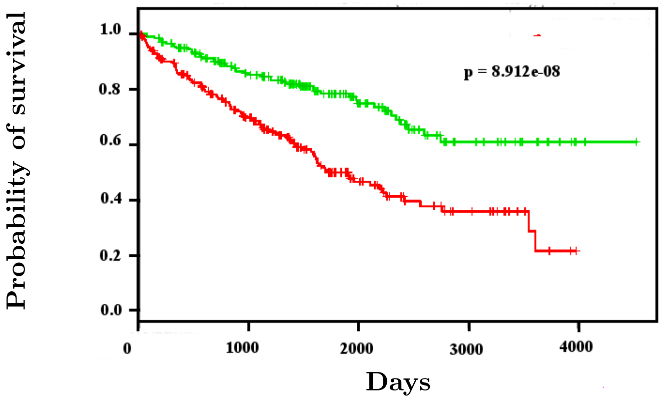

Figure Gii: KIRC Specific Top Gain

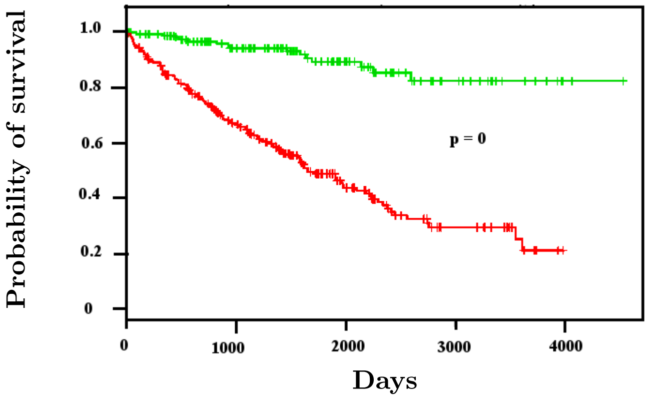

Figure Giii: KIRC Top Lost

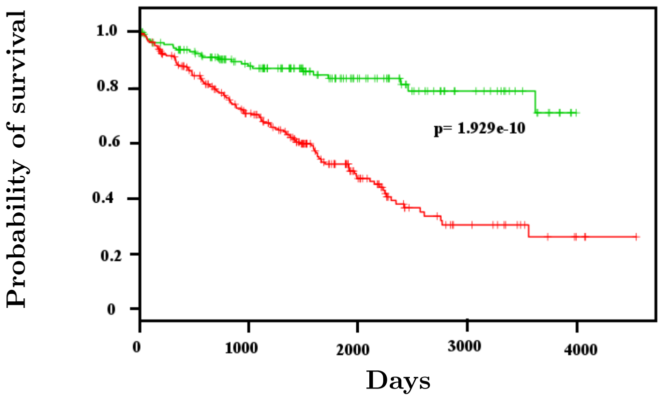

Figure Giv: KIRC Specific Top Lost

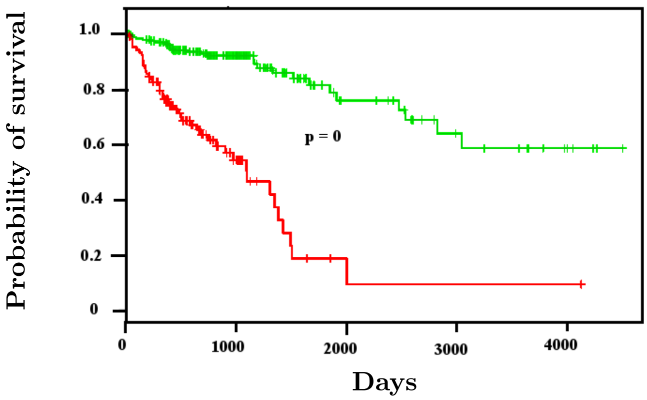

Figure Hi: COAD Top Gain

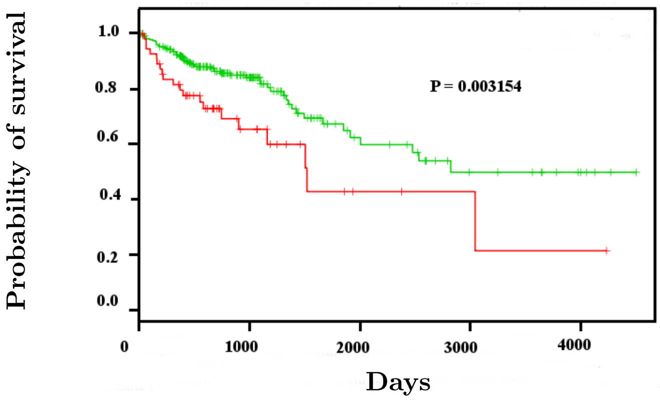

Figure Hii: COAD Specific Top Gain

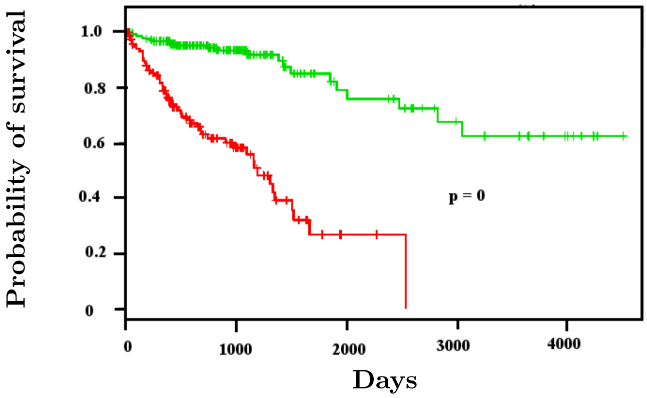

Figure Hiii: COAD Top Lost

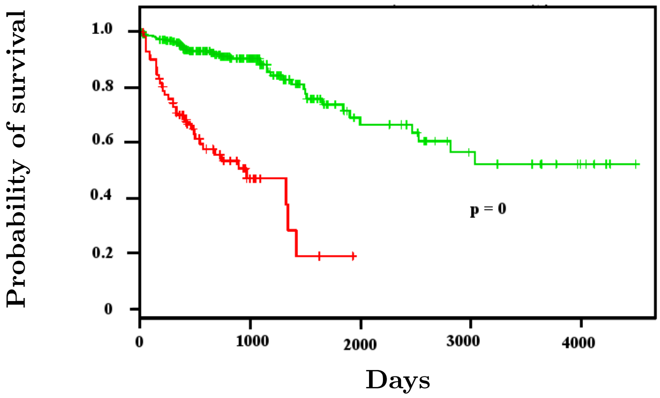

Figure Hiv: COAD Specific Top Lost

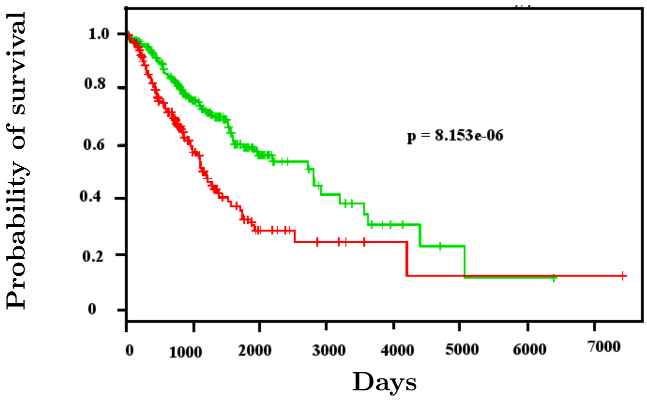

Figure Ii: STES Top Gain

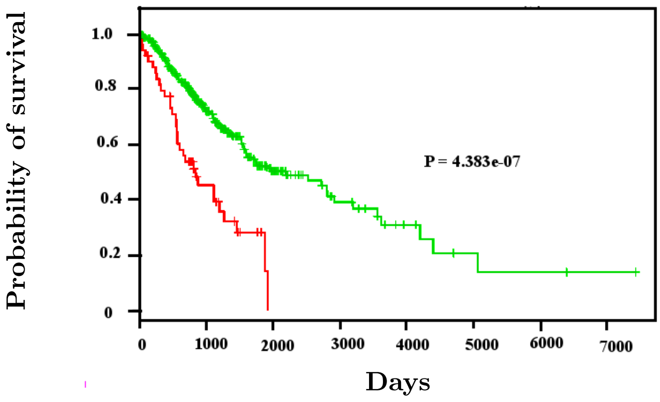

Figure Iii: STES Specific Top Gain

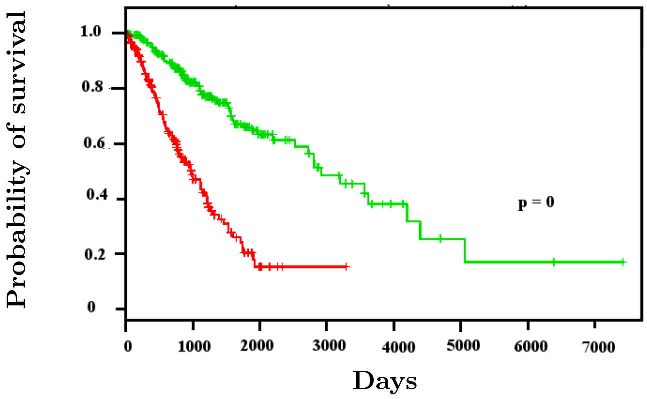

Figure Iiii: STES Top Lost

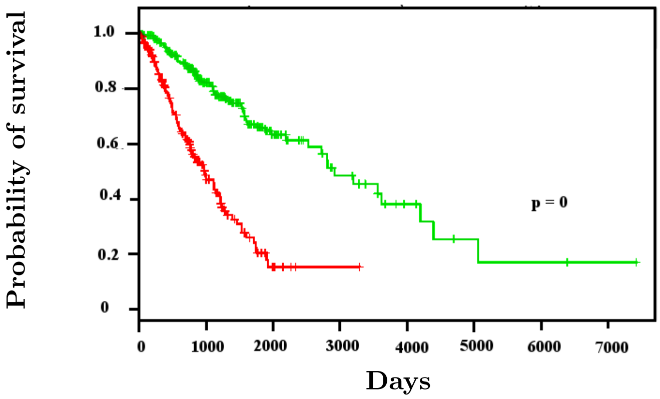

Figure Iiv: STES Specific Top Lost

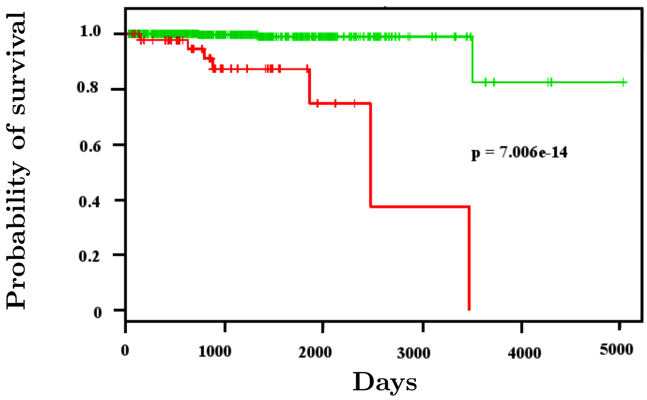

Figure Ji: PRAD Top Gain

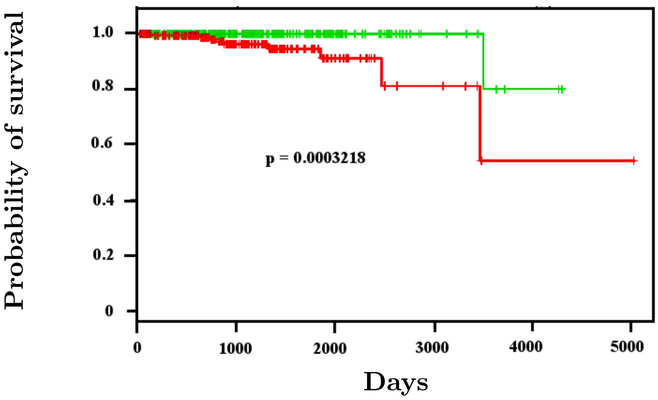

Figure Jii: PRAD Specific Top Gain

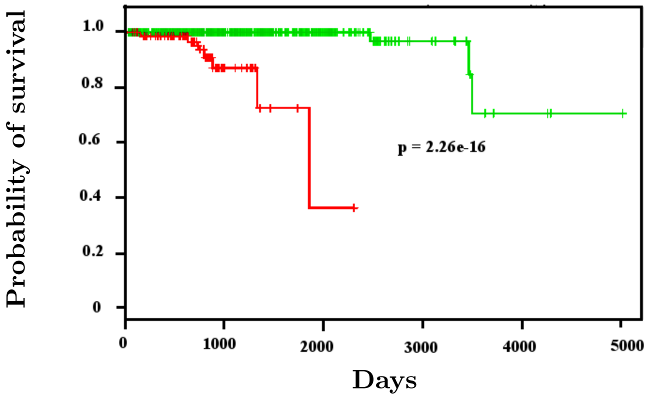

Figure Jiii: PRAD Top Lost

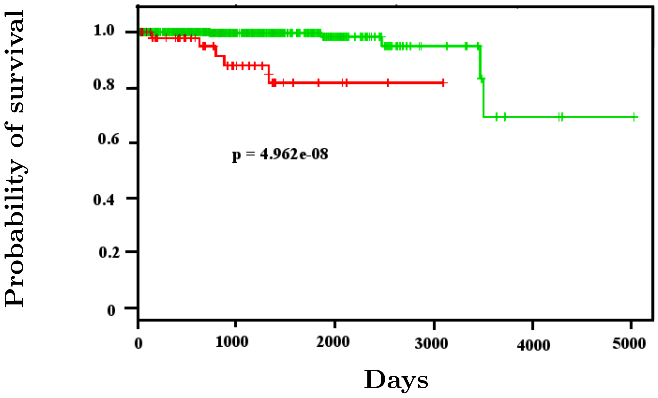

Figure Jiv: PRAD Specific Top Lost

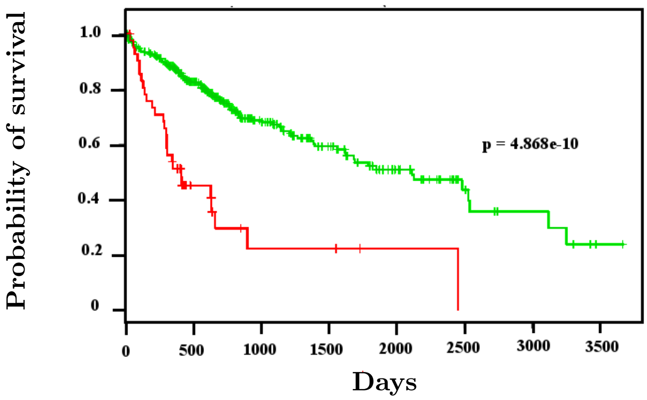

Figure Li: LIHC Specific Top Gain

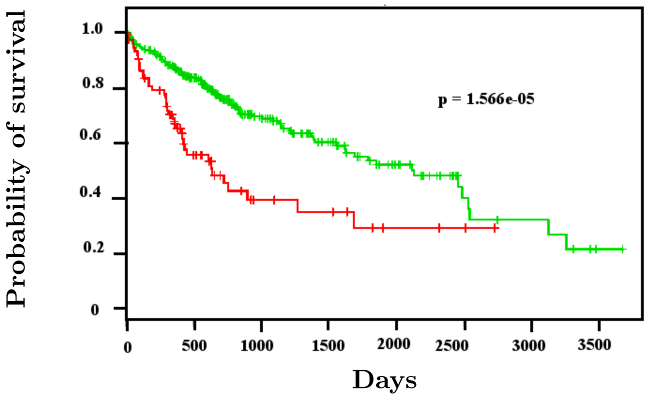

Figure Liii: LIHC Top Lost

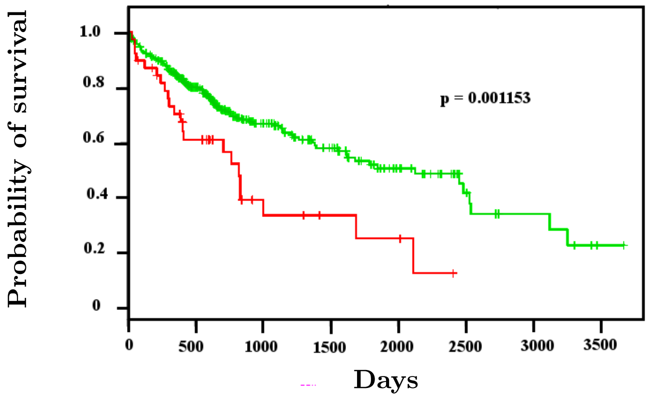

Figure Liv: LIHC Specific Top Lost

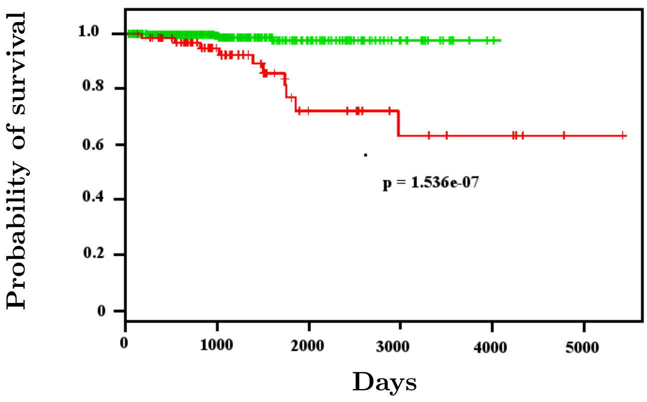

Figure Mi: THCA Top Gain

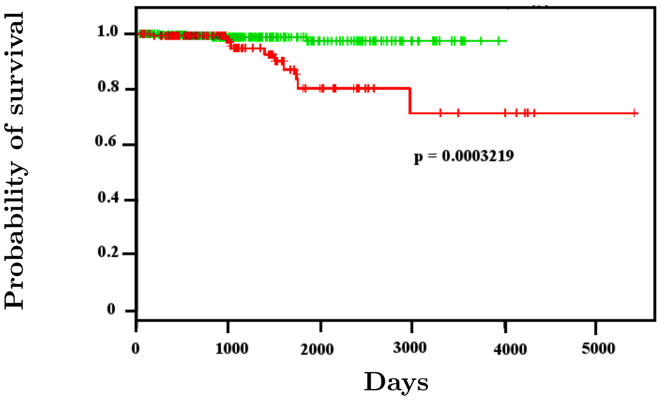

Figure Mii: THCA Specific Top Gain

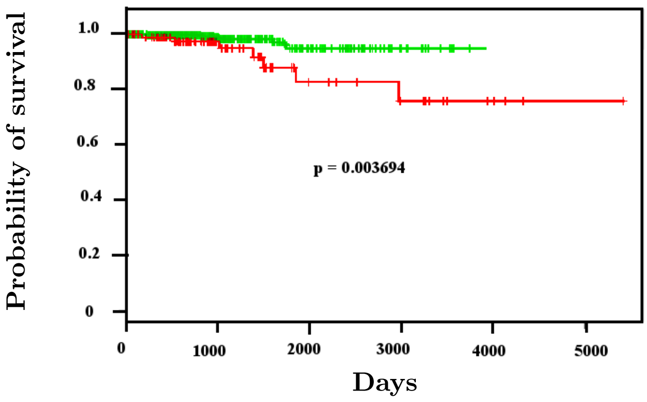

Figure Miii: THCA Top Lost

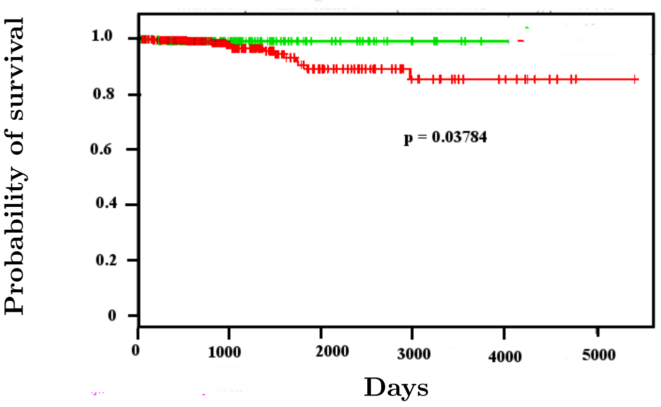

Figure Miv: THCA Specific Top Lost

S2 Fig (A - M): Kaplan-Meier survival analysis plots of multigene cancer biomarkers involved in edgetic perturbations. The x axes indicate the number of days until patient death whereas the y axes indicate the probability of patient survival. In all the figures, the green lines indicate better survival (longer life-span) after cancer diagnosis while the red lines indicate poor survival (shorter life-span) after cancer diagnosis as a result of the proteins involved in edgetic gains or losses. In all the cases, the proteins involved in edgteic perturbations predicted poor survival of the patients (Logrank test p-value < 0.05), indicating their importance in cancer monitoring and prognosis. (i) Overall survival predicted from gene signatures involved in edgetic gains across most patients of a cancer type (except for LIHC), (ii) Overall survival predicted from gene signatures involved in edgetic losses across most patients of a cancer type, (iii) Overall survival predicted from gene signatures involved in edgetic gains across patients showing cancer-specific perturbations, (iv) Overall survival predicted from gene signatures involved in edgetic losses across patients showing cancer-specific perturbations. The names of the prominent proteins with multiple perturbations responsible for the above observations can be found in S4 Table a and b.

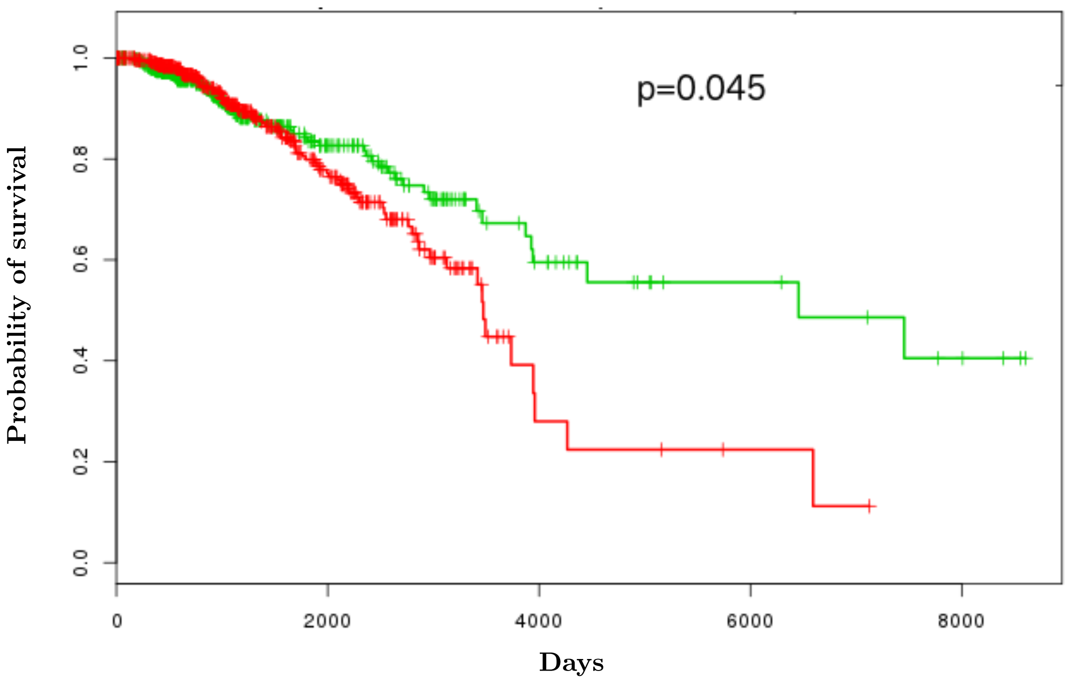

Figure Ai: BRCA gains

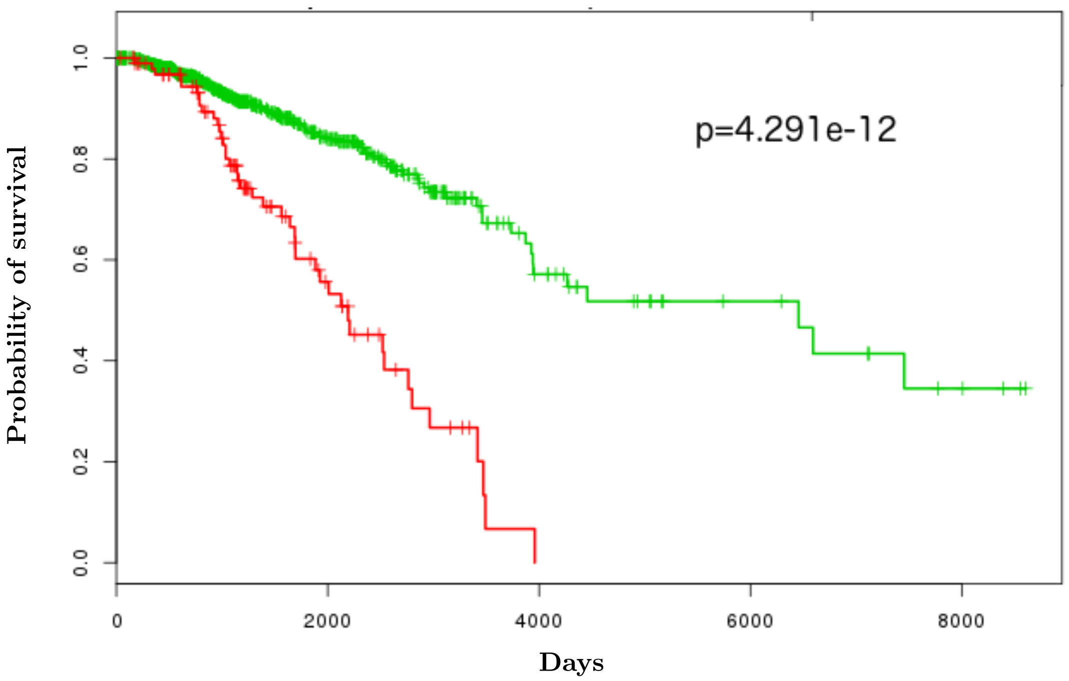

Figure Aii: BRCA losses

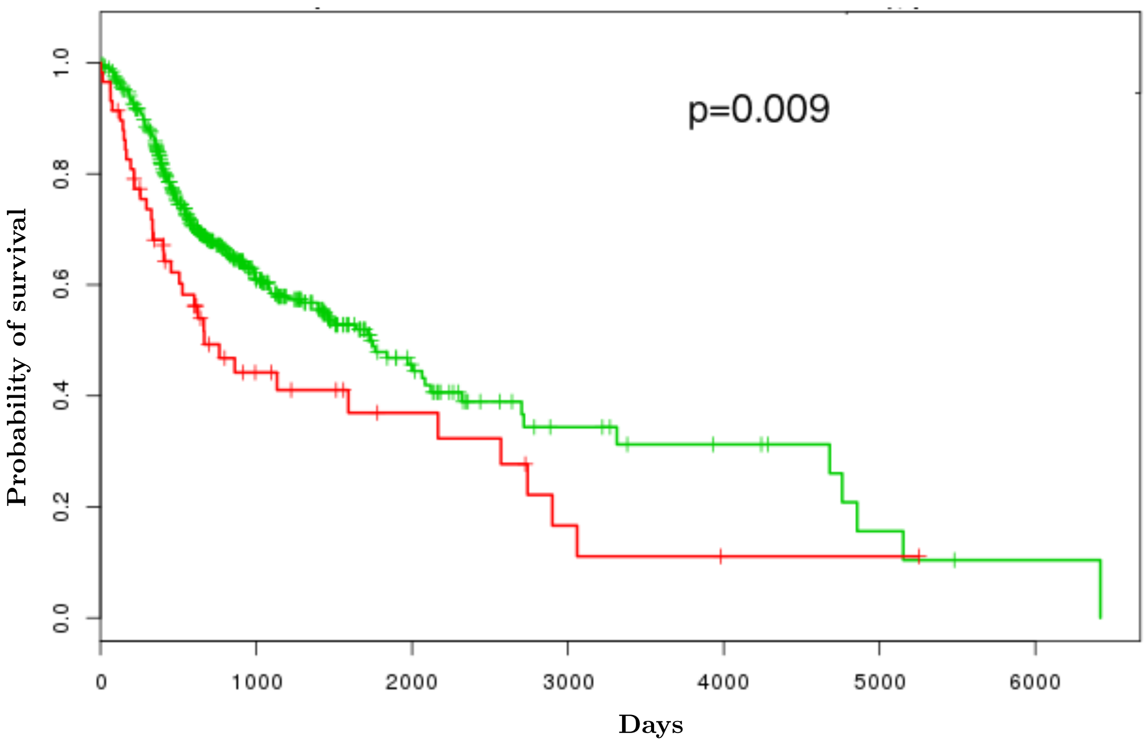

Figure Bi: HNSC gains

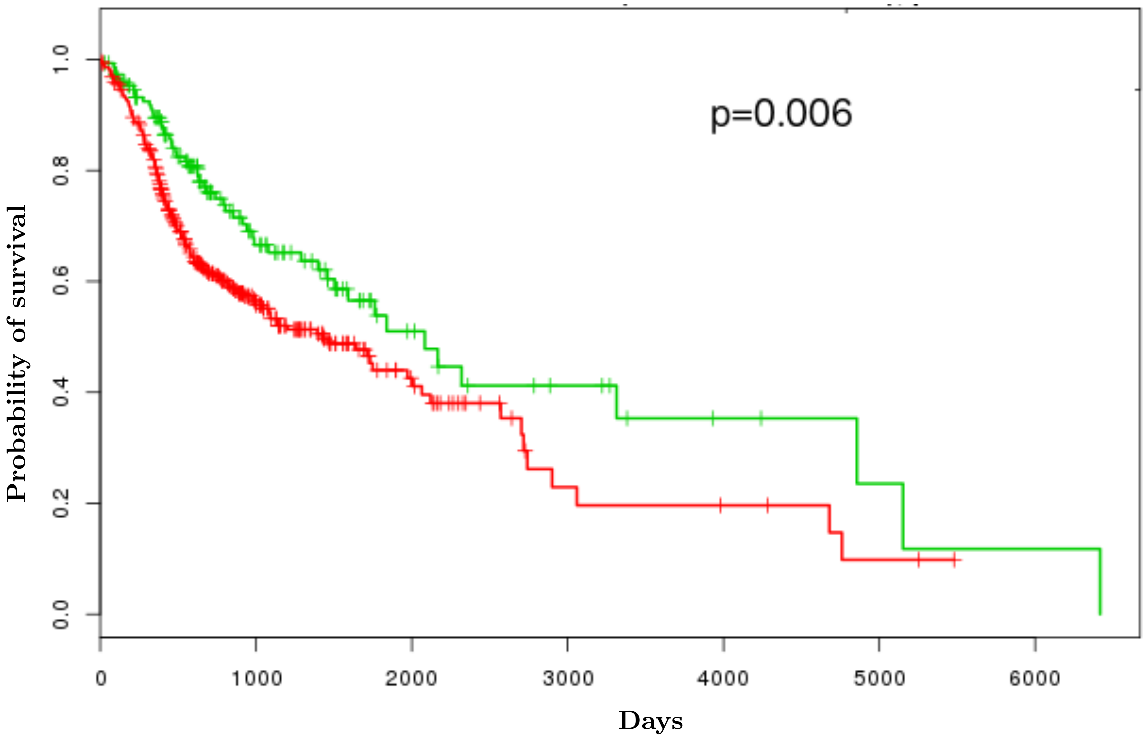

Figure Bii: HNSC losses

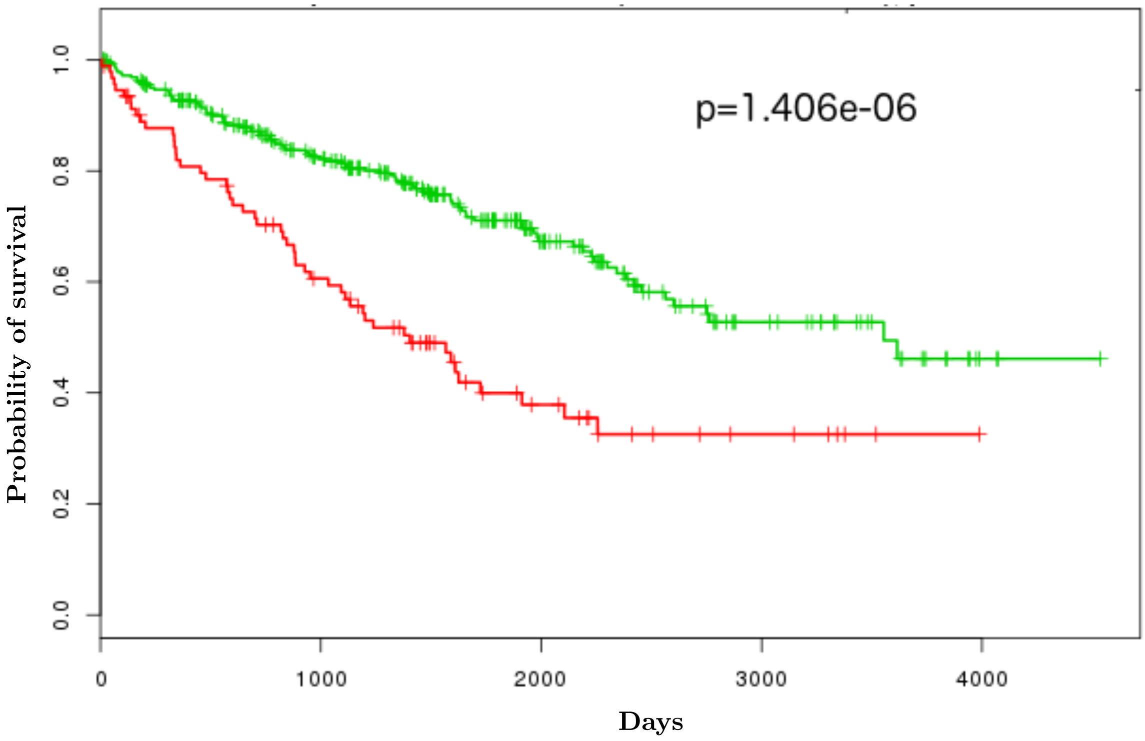

Figure Ci: KIRC gains

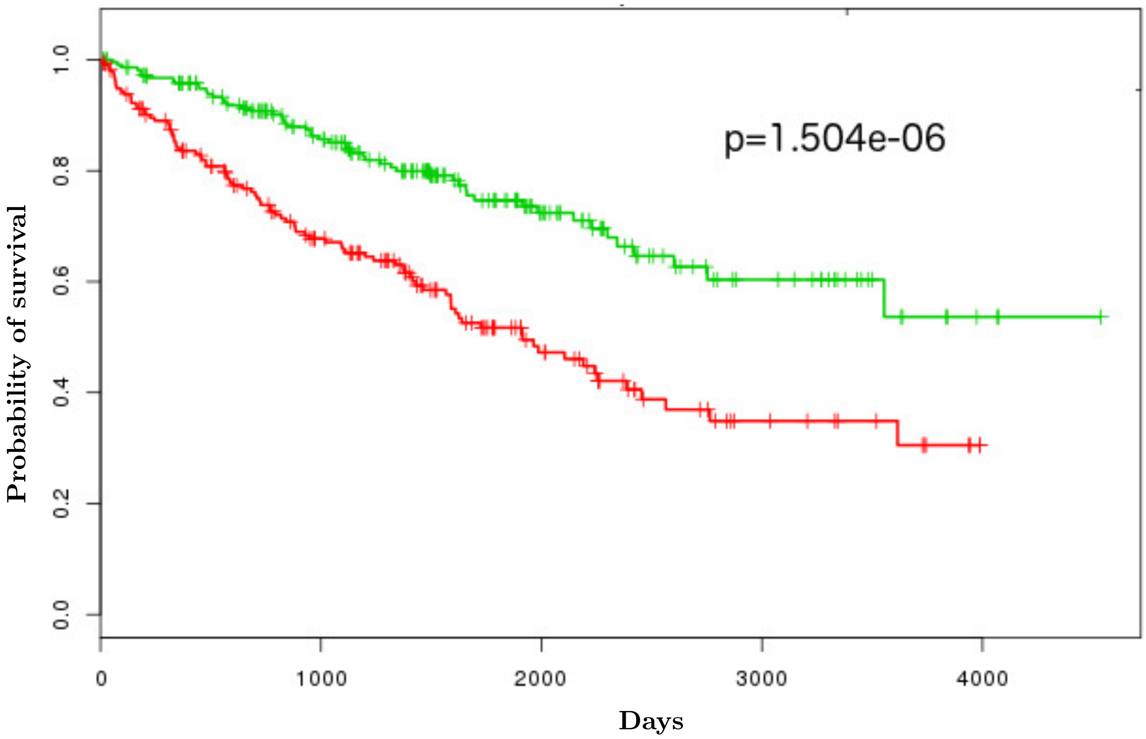

Figure Cii: KIRC losses

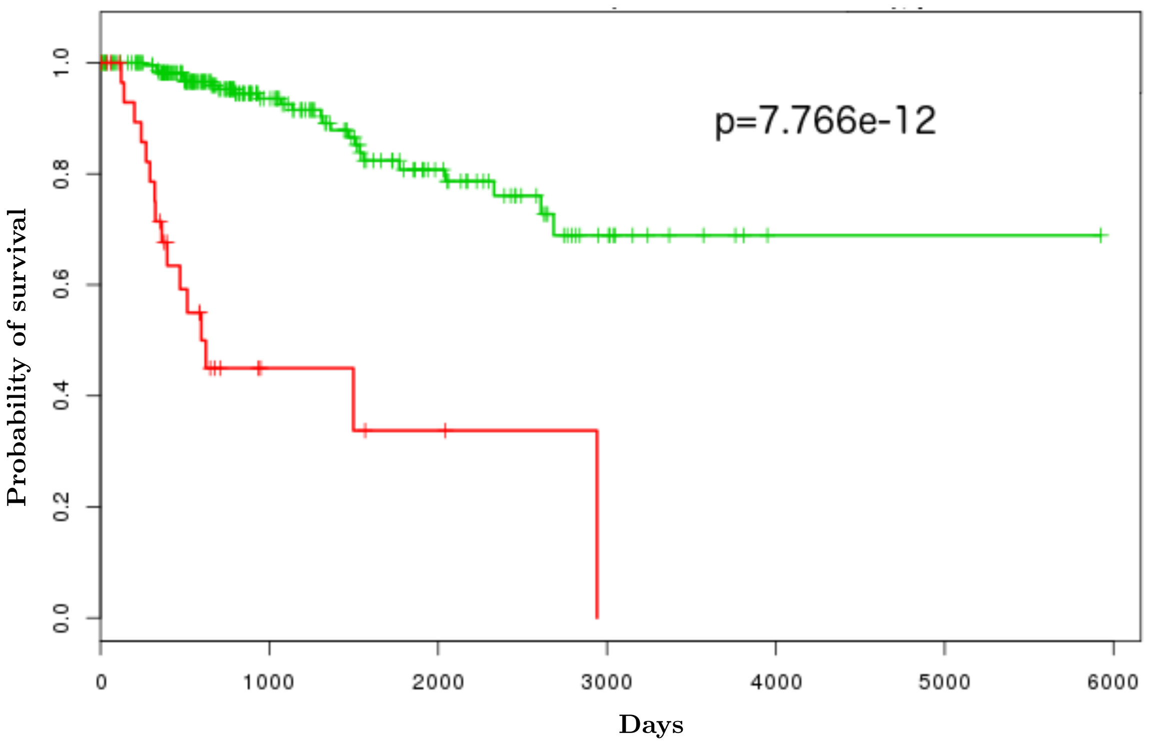

Figure Di: KIRP gains

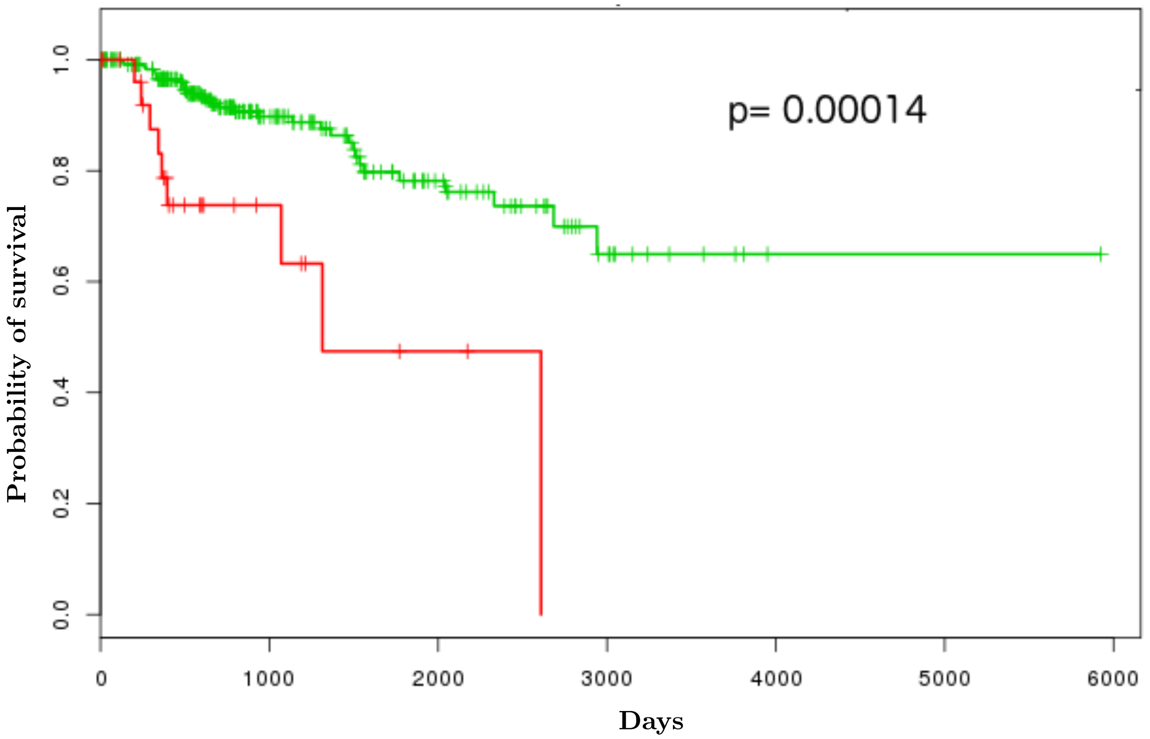

Figure Dii: KIRP losses

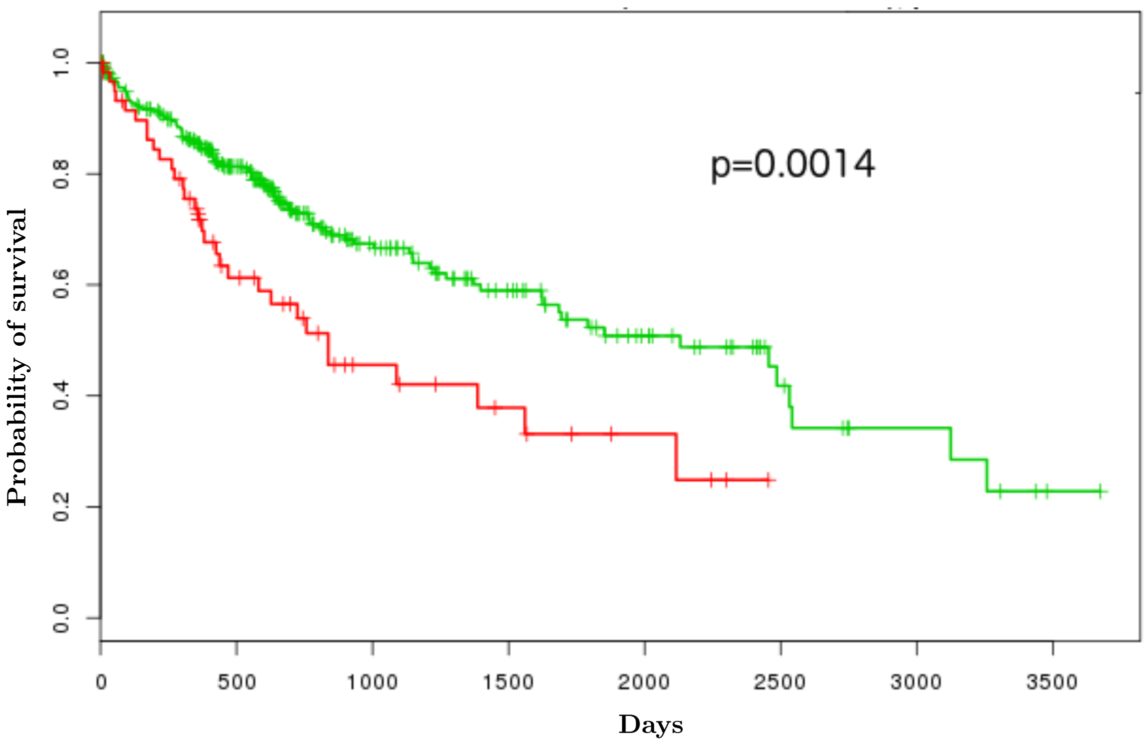

Figure Ei: LIHC gains

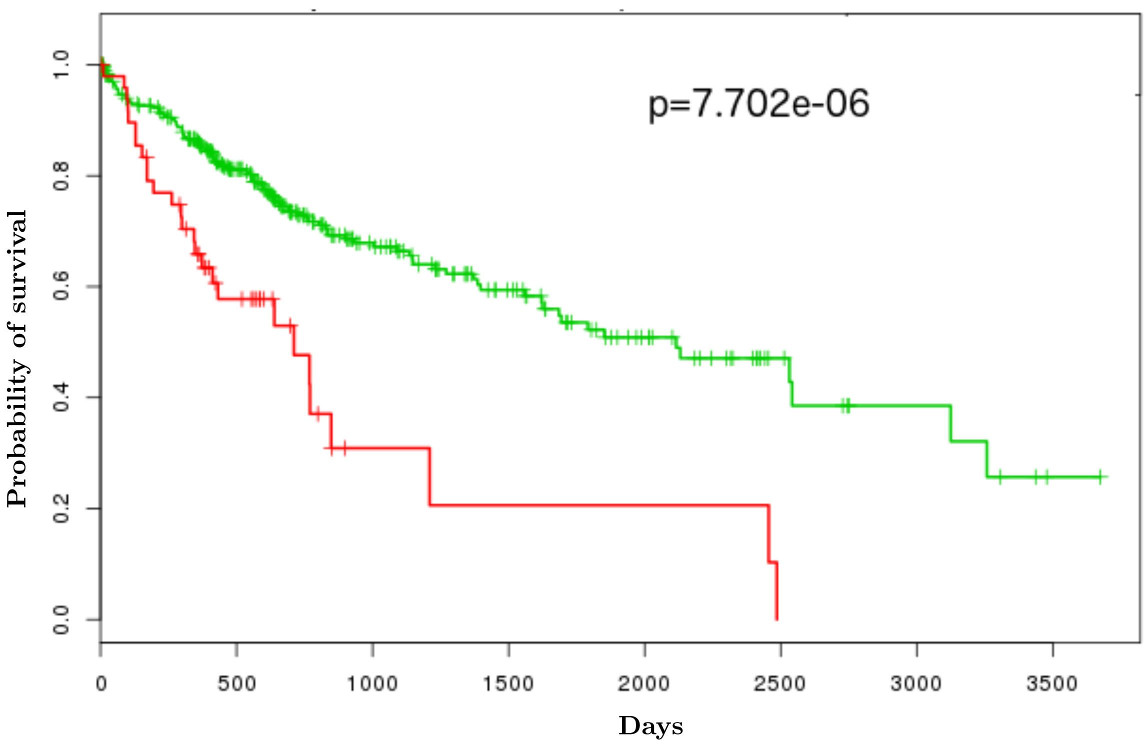

Figure Eii: LIHC losses

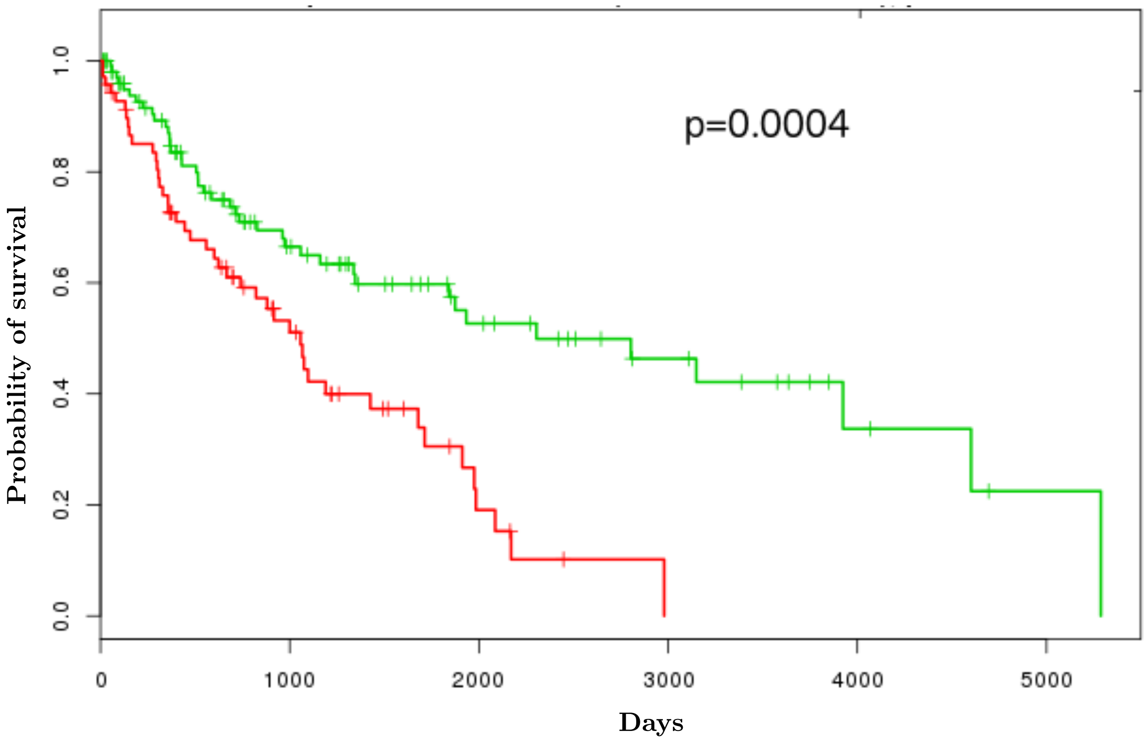

Figure Fi: LUSC gains

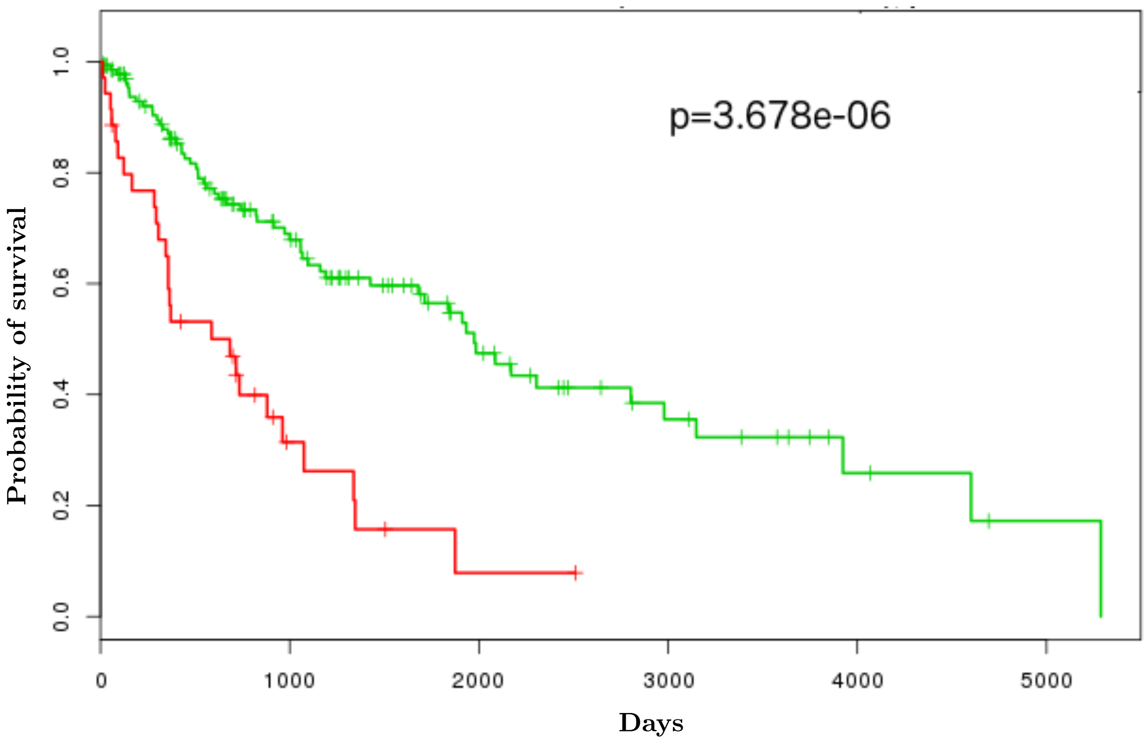

Figure Fii: LUSC losses

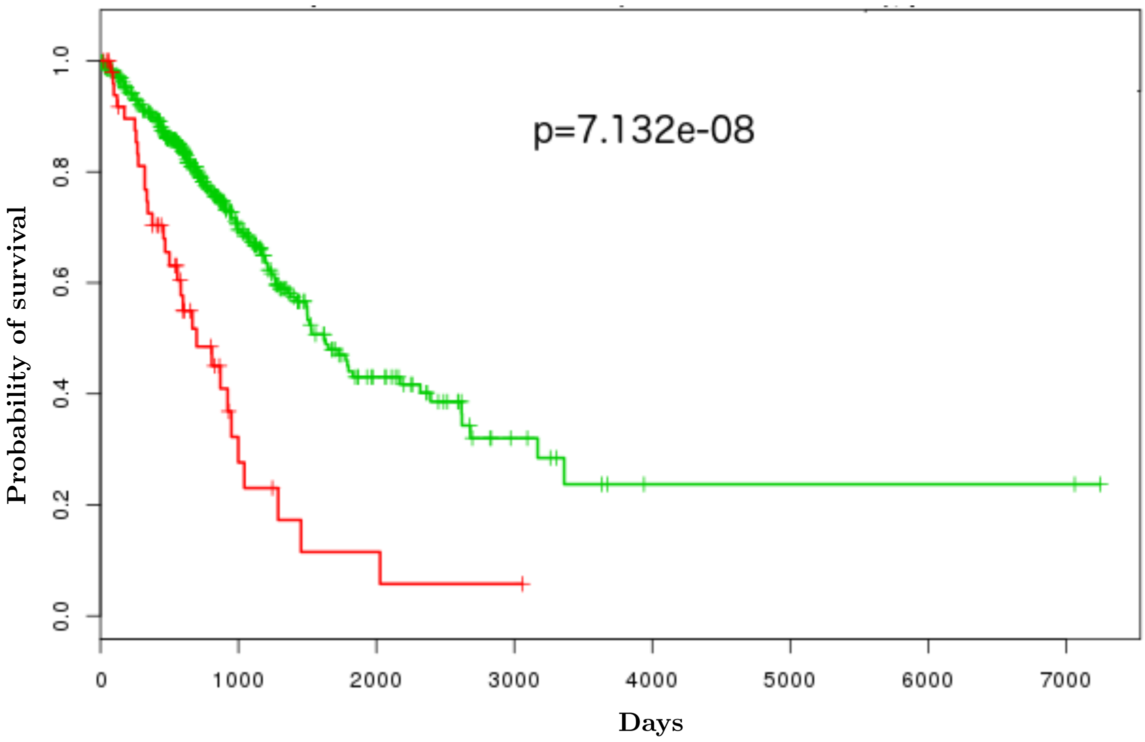

Figure Gi: LUAD gains

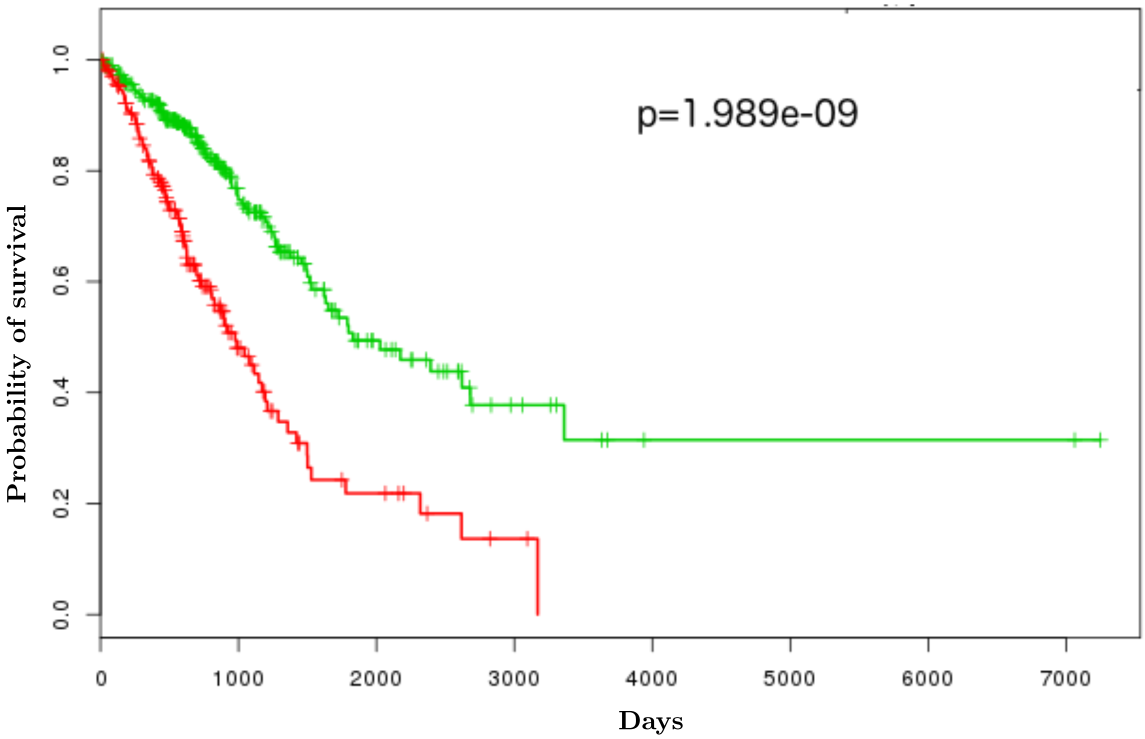

Figure Gii: LUAD losses

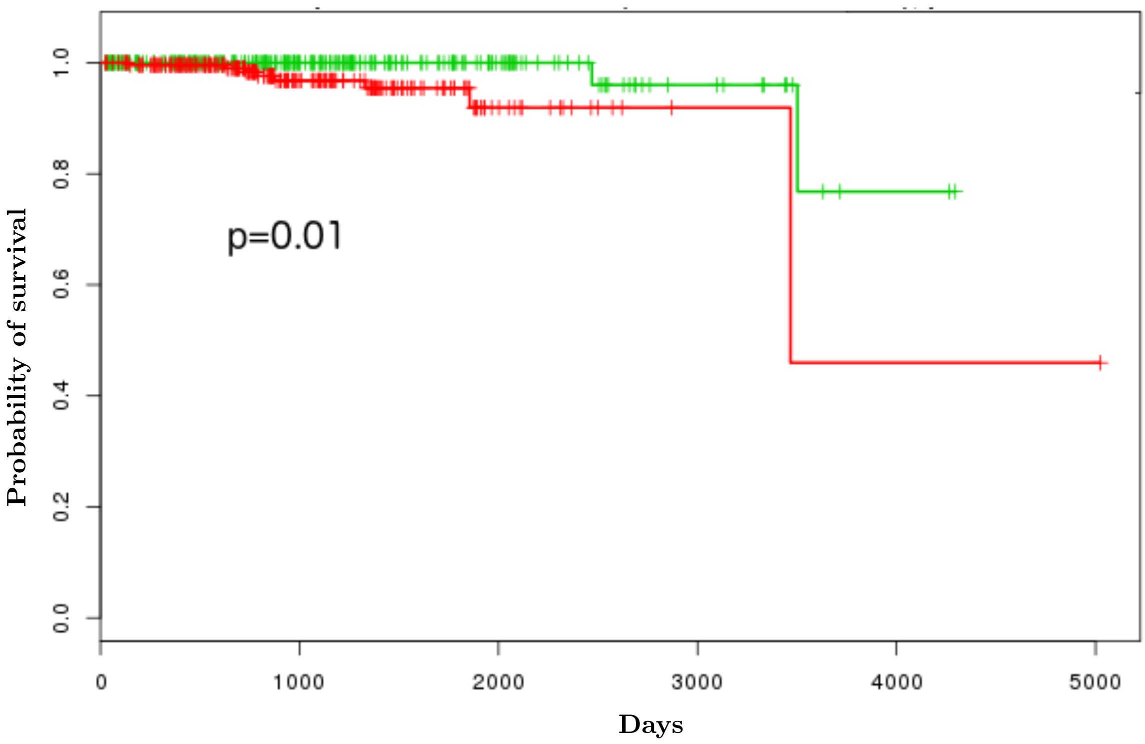

Figure Hi: PRAD gains

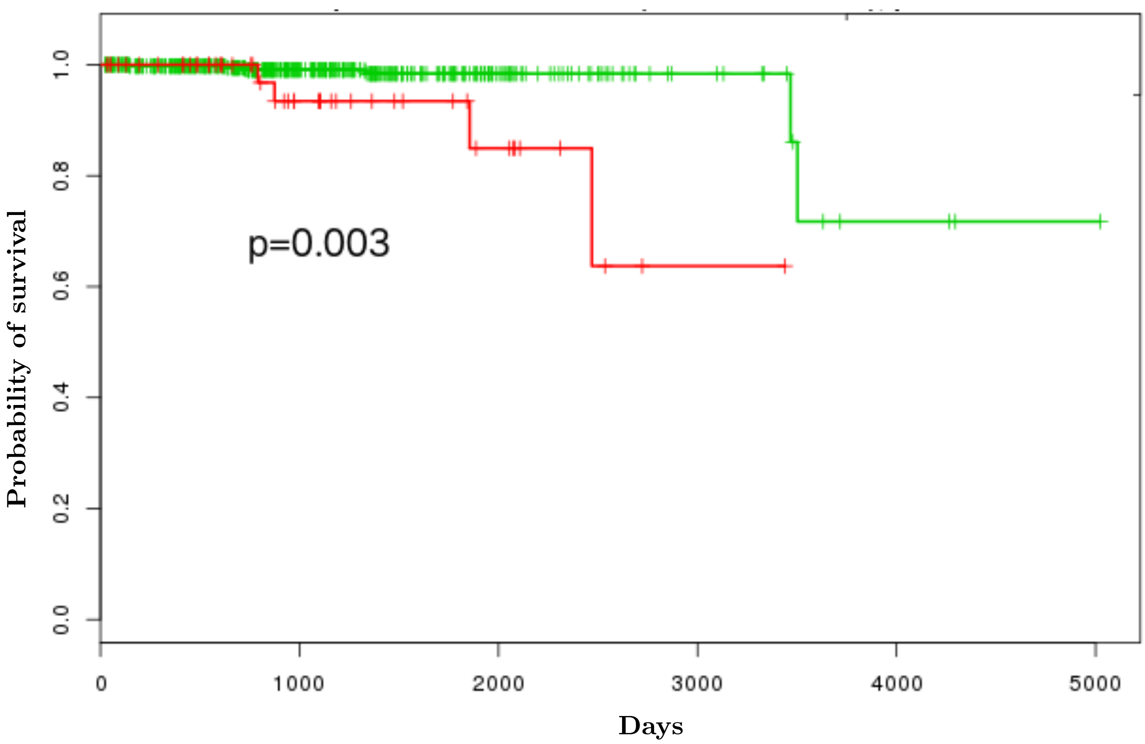

Figure Hii: PRAD losses

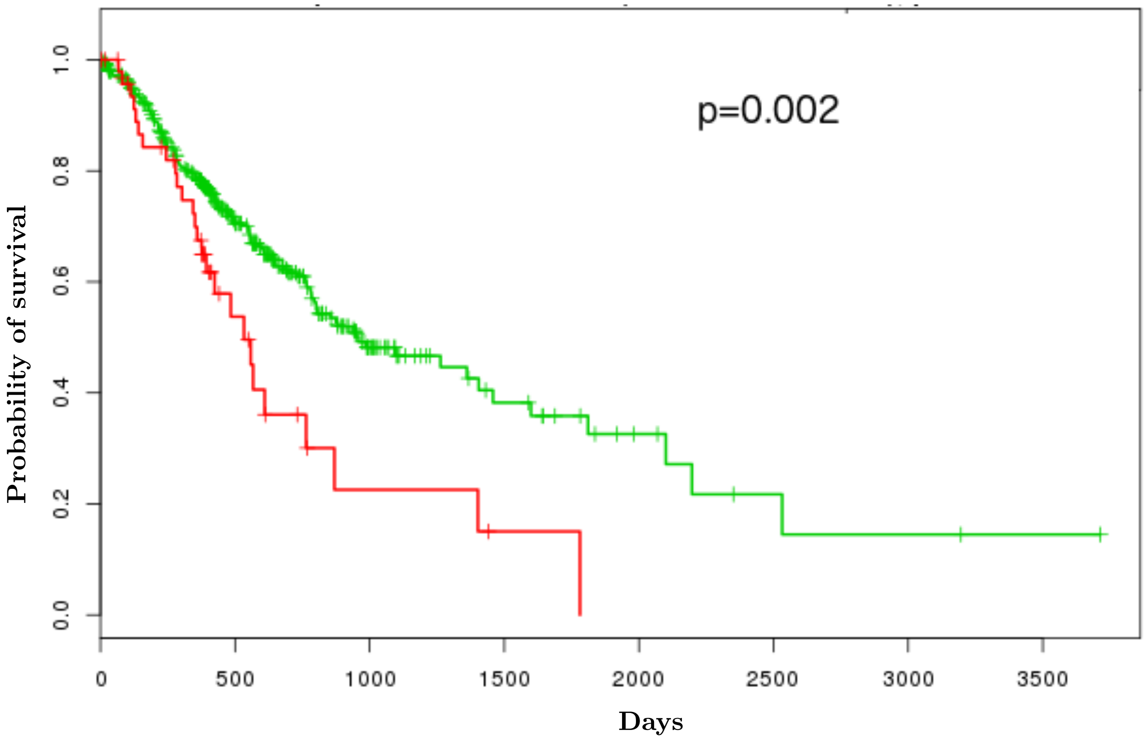

Figure Ii: STES gains

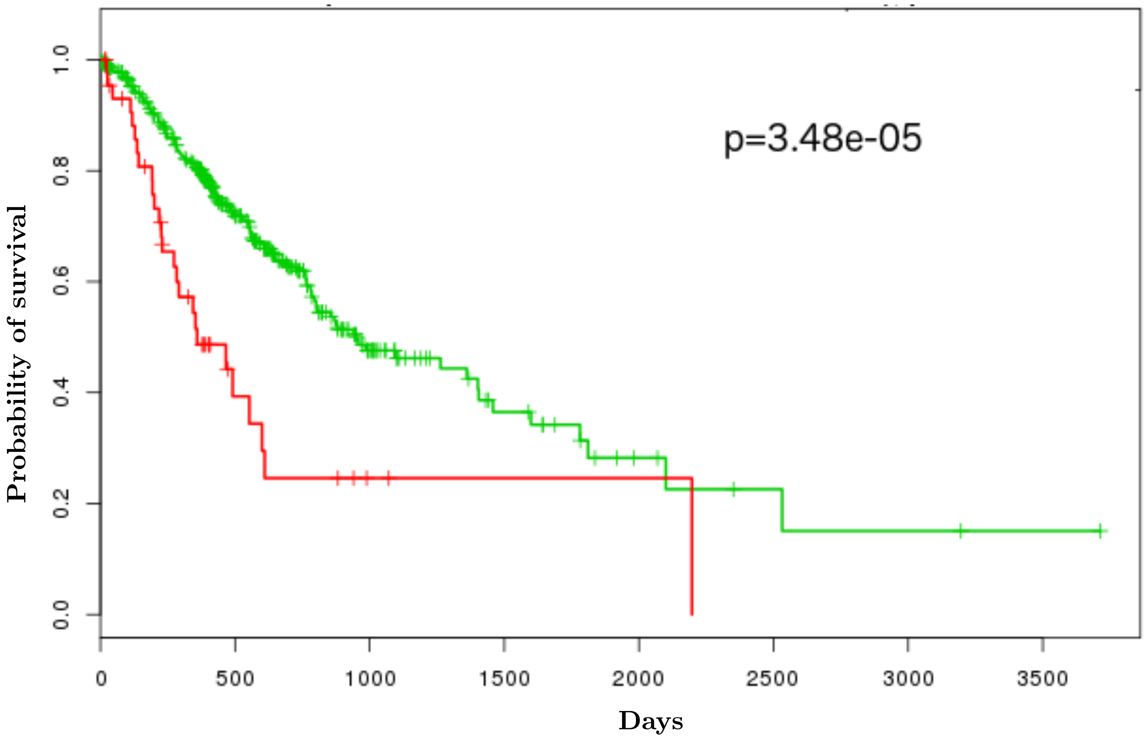

Figure Iii: STES losses

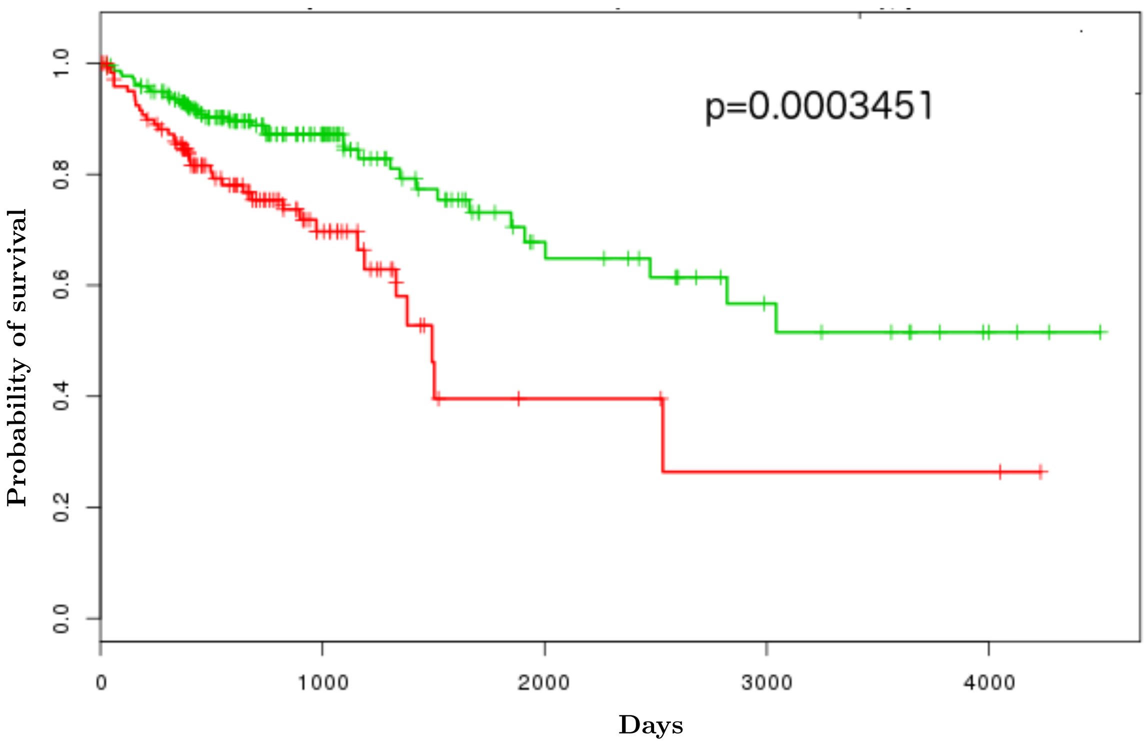

Figure Ji: COAD losses

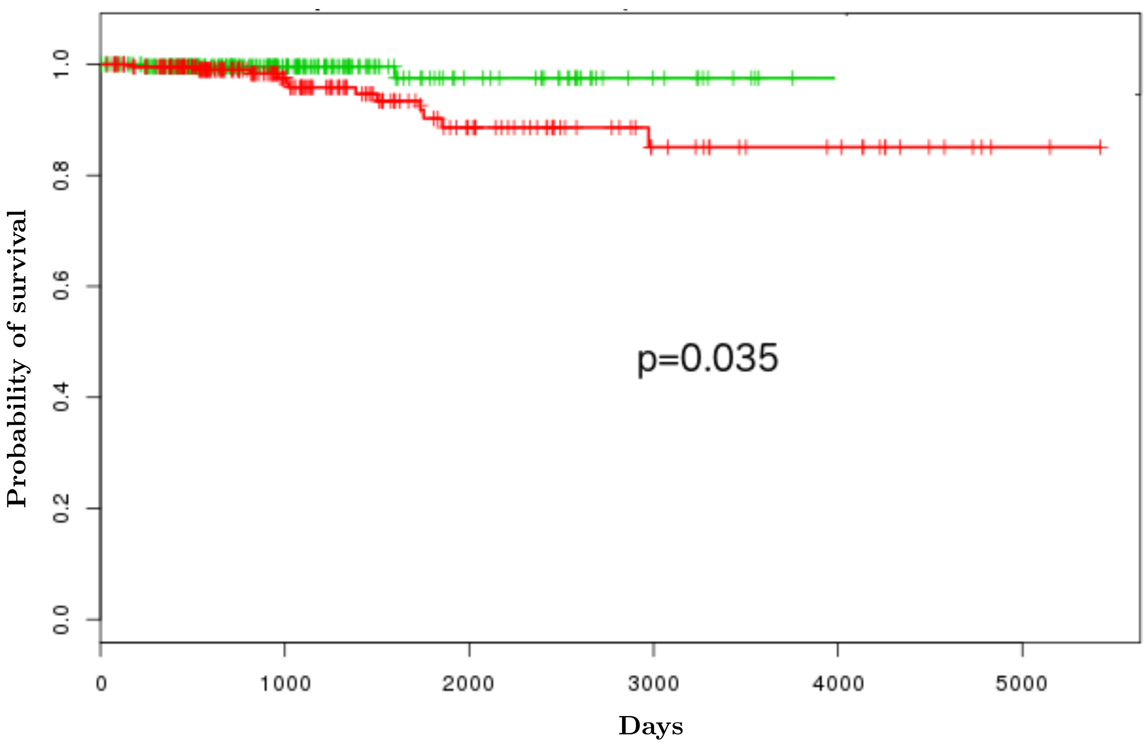

Figure Ki: THCA losses

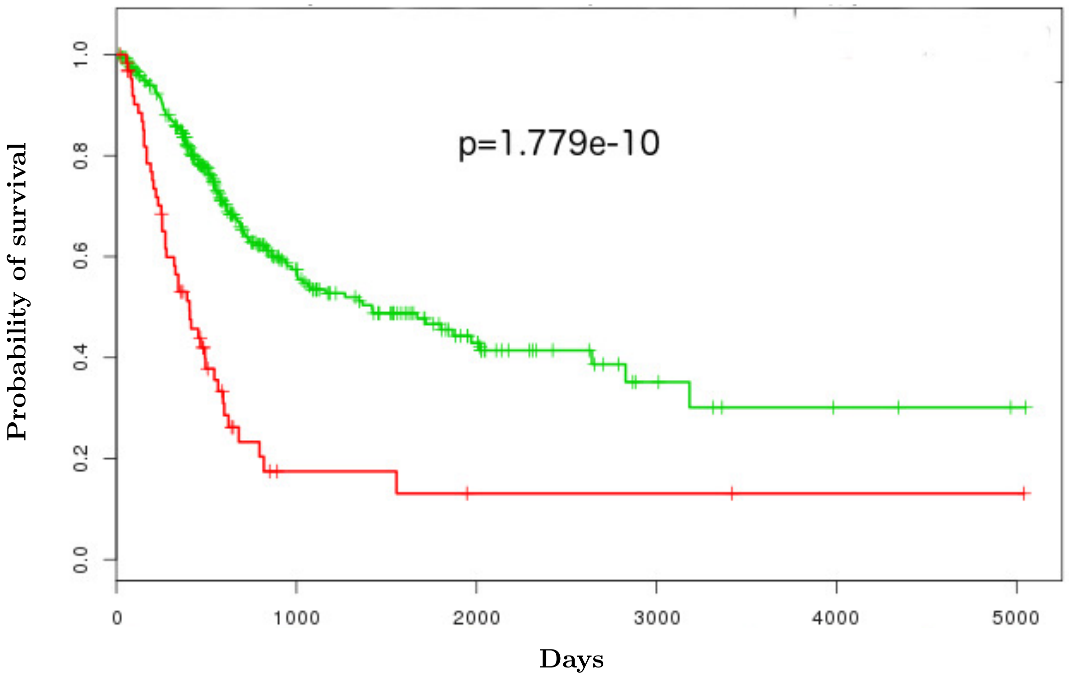

Figure Li: BLCA gains

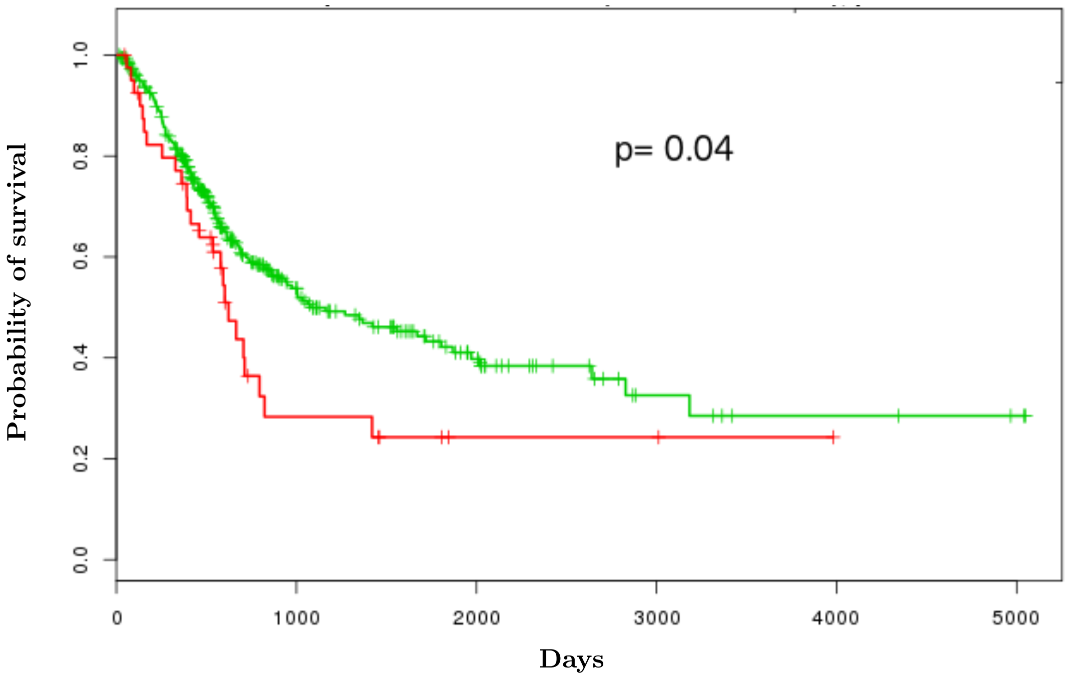

Figure Lii: BLCA losses

S3 Fig (A - L): Kaplan-Meier survival analysis plots of multigene cancer biomarkers involved in edgetic perturbations as a result of Isoform/domain chnages in the cancer state. The x axes indicate the number of days until patient death whereas the y axes indicate the probability of patient survival. In all the figures, the green lines indicate better survival (longer life-span) after cancer diagnosis while the red lines indicate poor survival (shorter life-span) after cancer diagnosis as a result of the proteins involved in edgetic gains or losses. In all the cases, the proteins involved in edgteic perturbations predicted poor survival of the patients (Logrank test p-value < 0.05), indicating their importance in cancer monitoring and prognosis. (i) Overall survival predicted from gene signatures involved in edgetic gains across most patients of a cancer type, (ii) Overall survival predicted from gene signatures involved in edgetic losses across most patients of a cancer type. The names of the prominent proteins with multiple perturbations responsible for the above observations can be found in S4 Table 1a (marked with *).**

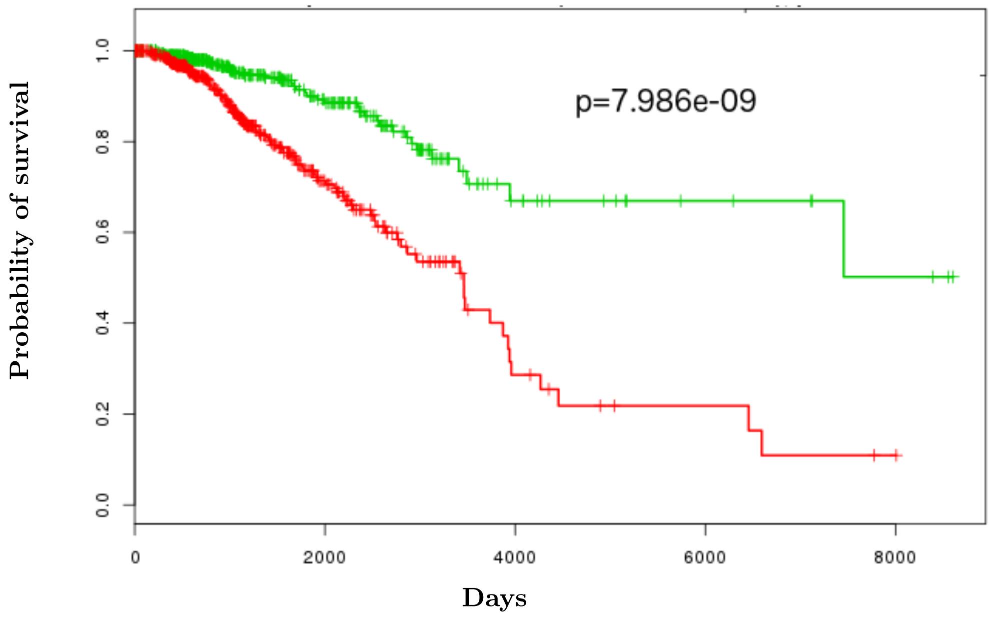

Figure a: BRCA

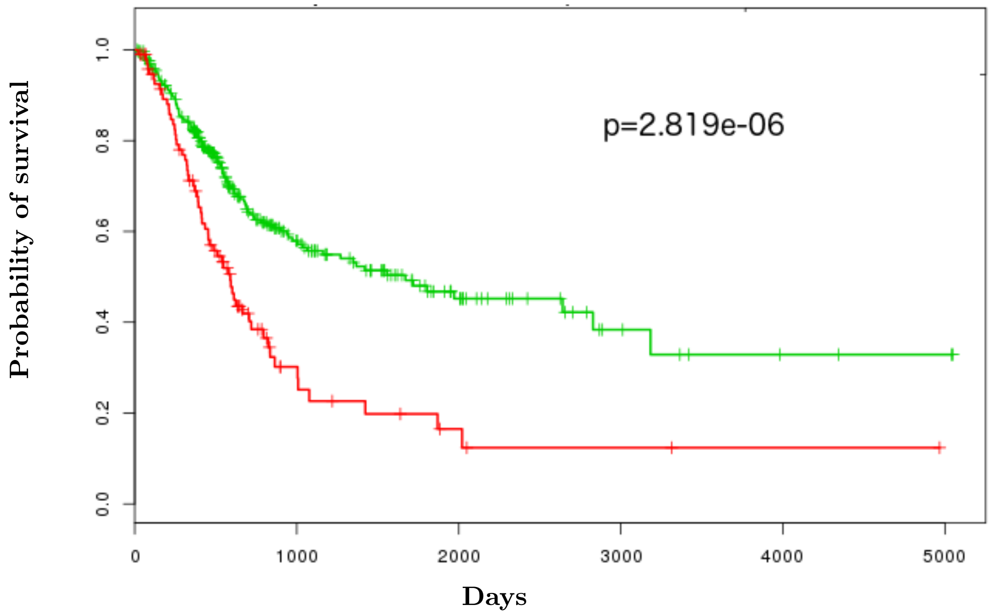

Figure b: BLCA

S4 Fig (a - b): Kaplan-Meier survival analysis plots of multigene cancer biomarkers involved in edgetic perturbations from the randomised PPIN. The x axes indicate the number of days until patient death whereas the y axes indicate the probability of patient survival. In both the figures, the green lines indicate better survival (longer life-span) after cancer diagnosis while the red lines indicate poor survival (shorter life-span) after cancer diagnosis as a result of the proteins involved in edgetic gains or losses. In all the cases, the proteins involved in edgteic perturbations predicted poor survival of the patients (Logrank test p-value $<$ 0.05), indicating their importance in cancer monitoring and prognosis. (A) Overall survival predicted from gene signatures involved in edgetic gains across most patients in BRCA, (B) Overall survival predicted from gene signatures involved in edgetic gains across most patients in BLCA. The names of the prominent proteins with multiple perturbations responsible for the above observations can be found in S4 Table a.

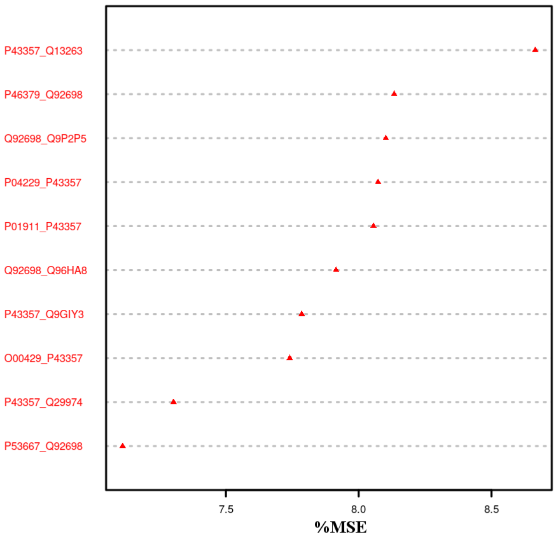

Figure a: Set one of important gained edges across cancer types

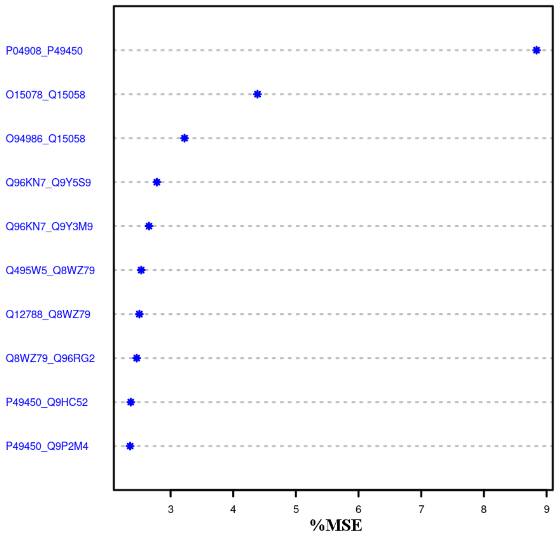

Figure b: Set two of important gained edges arosss cancer types

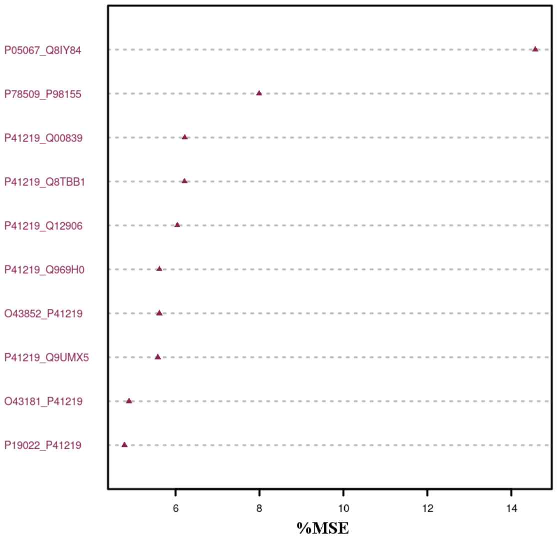

Figure c: Set one of important egdetic losses across cancer types

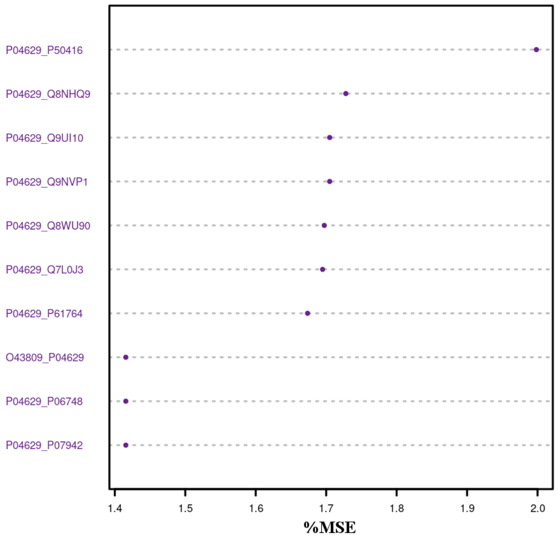

Figure d: Set two of important egdetic losses across cancer types

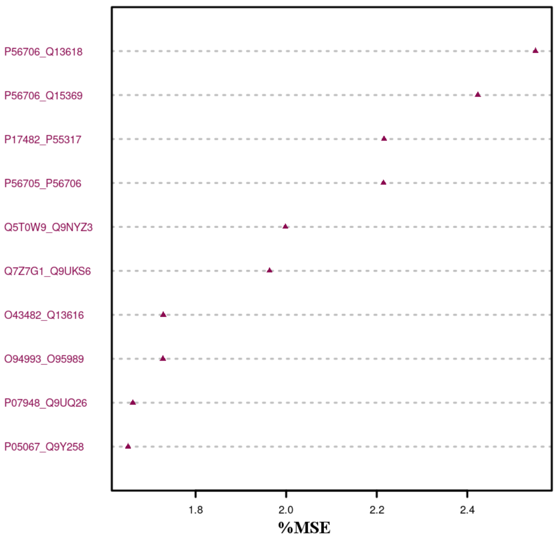

Figure e: Set one of important egdetic losses and gains across cancer types

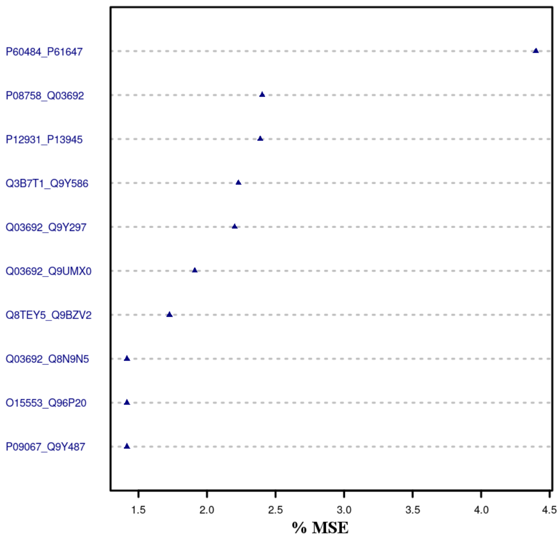

Figure f: Set two of important egdetic losses and g across cancer types

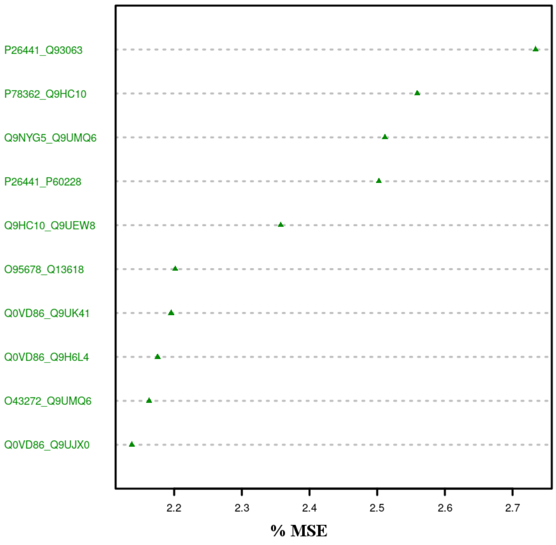

Figure g: Set three of important egdetic losses and gains across cancer types

S5 Fig (a-g): Top ranked features (edges) from the Random Forest algorithm that distinguish cancer types based on the identified groups from hierarchical clustering (Figure 5 of main text). The x axes indicate the percentage (%) Mean Squared Error (MSE2). The higher the %MSE of the feature (perturbed edge), the more important the perturbed edge is in identifyng a cluster.

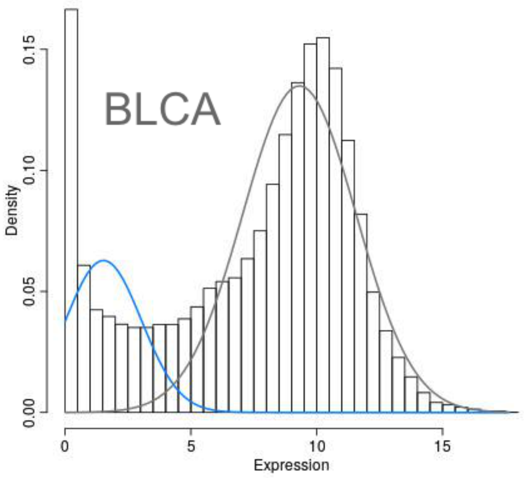

Figure A: Distribution of gene expression data in BLCA cancer samples

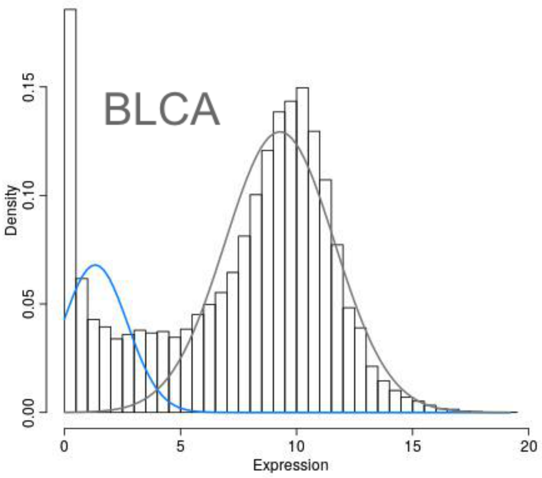

Figure A: Distribution of gene expression data in BLCA paired healthy samples

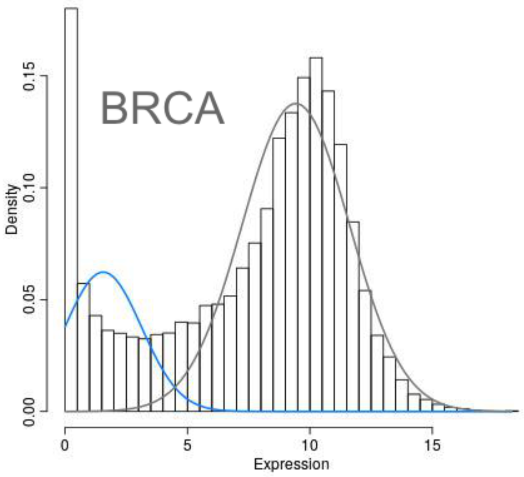

Figure B: Distribution of gene expression data in BRCA cancer samples

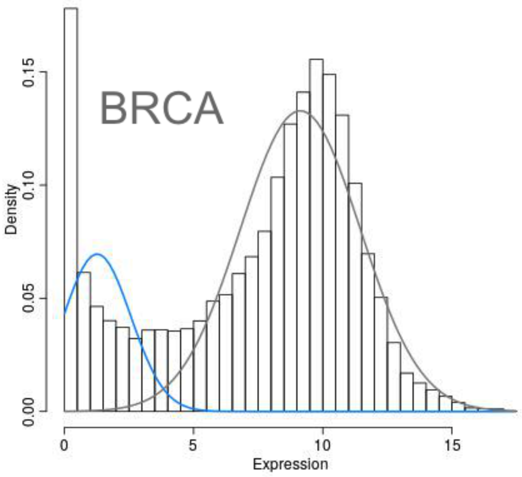

Figure B: Distribution of gene expression data in BRCA paired healthy samples

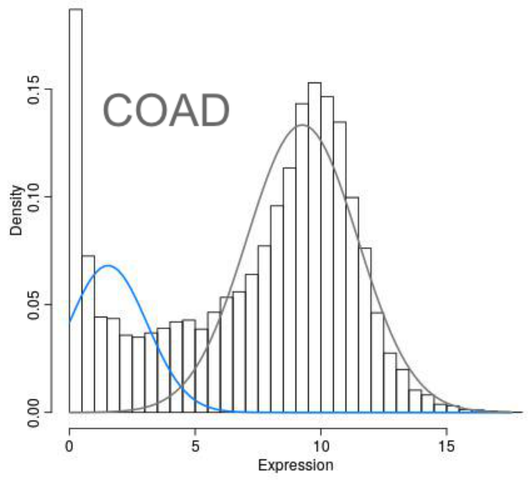

Figure C: Distribution of gene expression data in COAD cancer samples

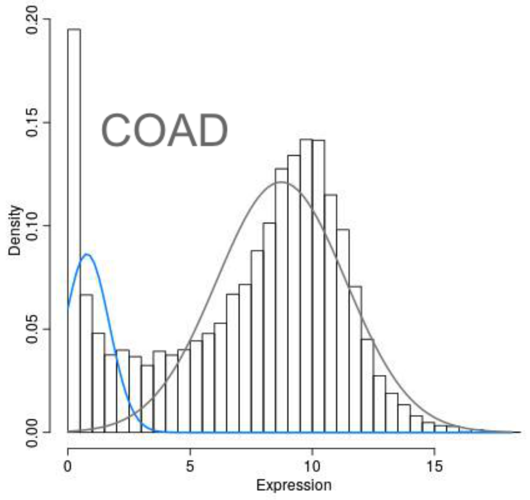

Figure C: Distribution of gene expression data in COAD paired healthy samples

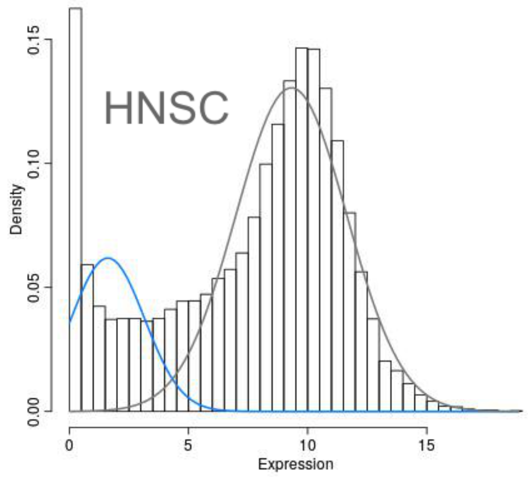

Figure D: Distribution of gene expression data in HNSC cancer samples

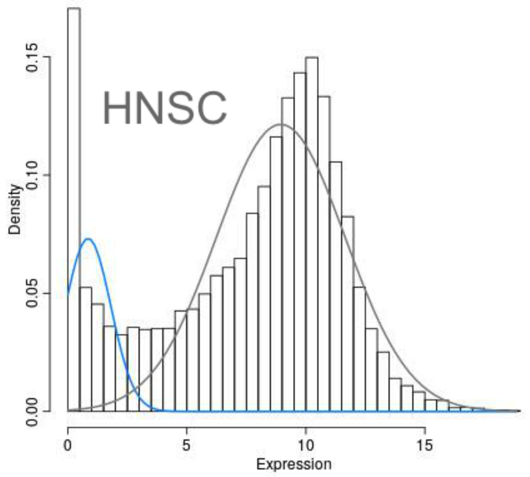

Figure D: Distribution of gene expression data in HNSC paired healthy samples

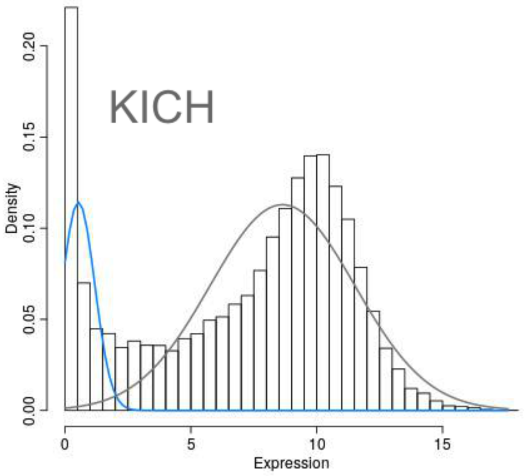

Figure E: Distribution of gene expression data in KICH cancer samples

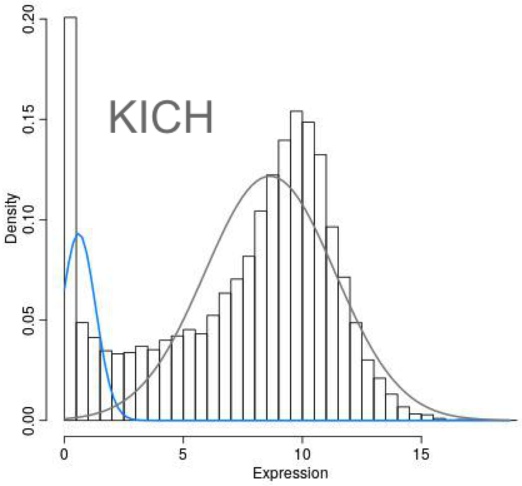

Figure E: Distribution of gene expression data in KICH paired healthy samples

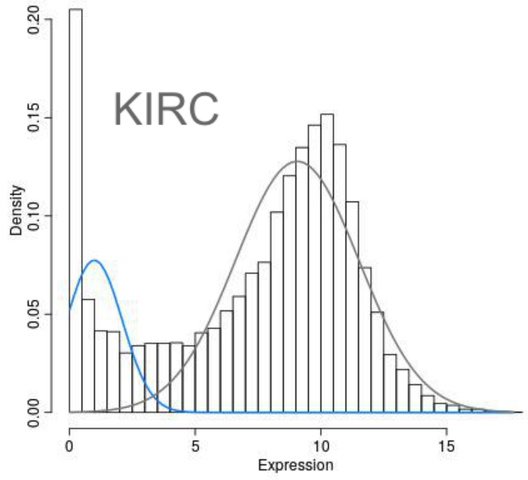

Figure A: Distribution of gene expression data in KIRC cancer samples

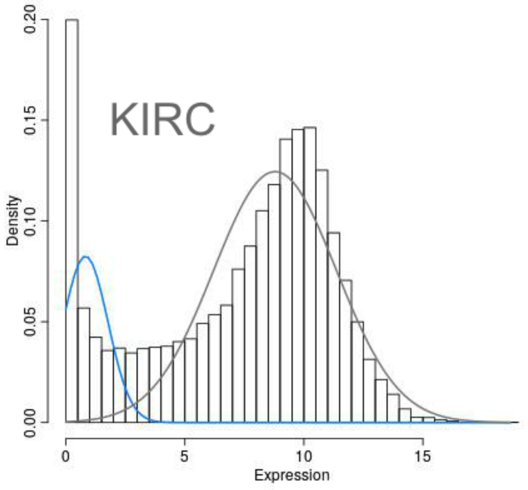

Figure A: Distribution of gene expression data in KIRC paired healthy samples

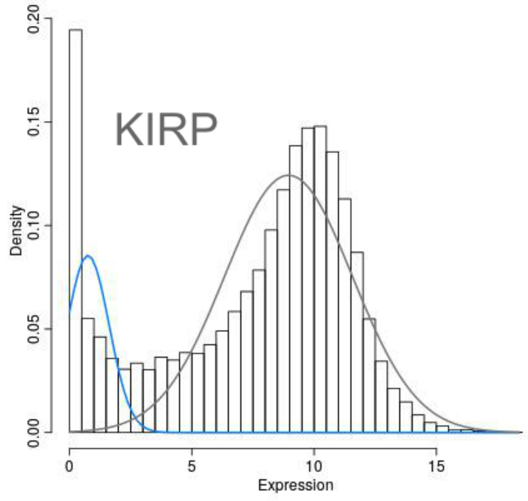

Figure B: Distribution of gene expression data in KIRP cancer samples

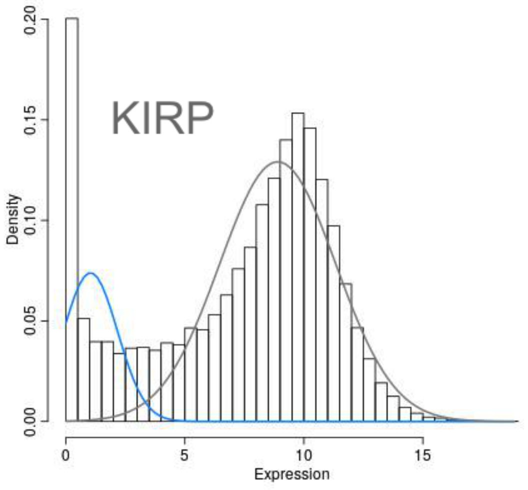

Figure B: Distribution of gene expression data in KIRP paired healthy samples

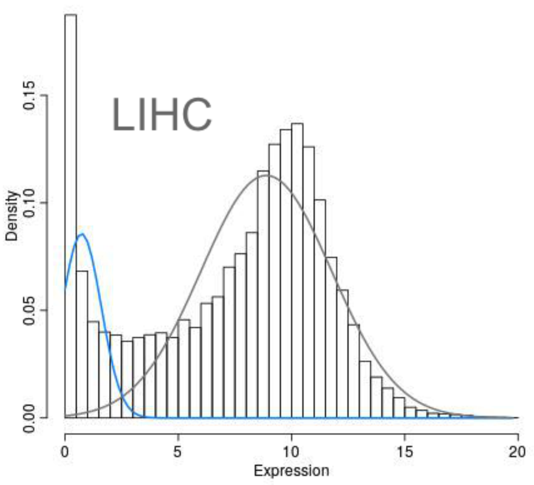

Figure C: Distribution of gene expression data in LIHC cancer samples

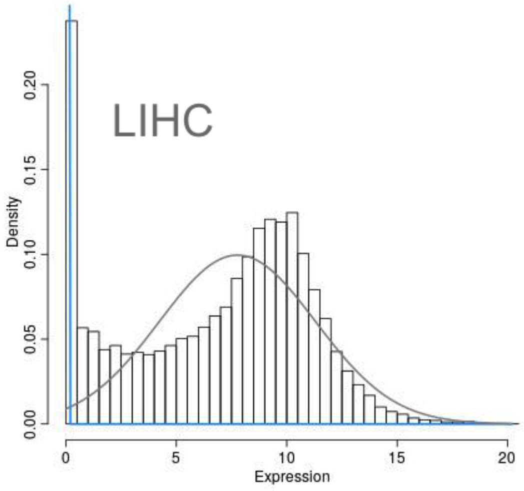

Figure C: Distribution of gene expression data in LIHC paired healthy samples

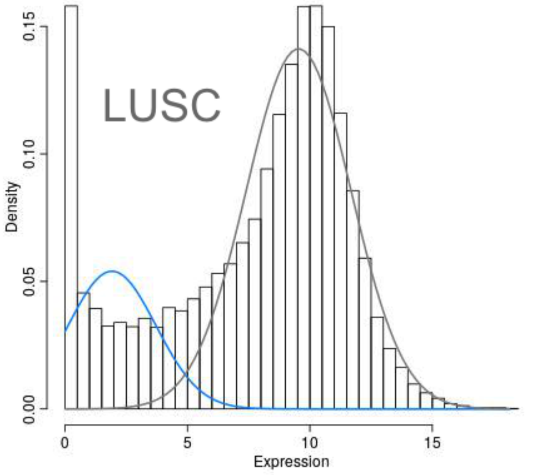

Figure D: Distribution of gene expression data in LUSC cancer samples

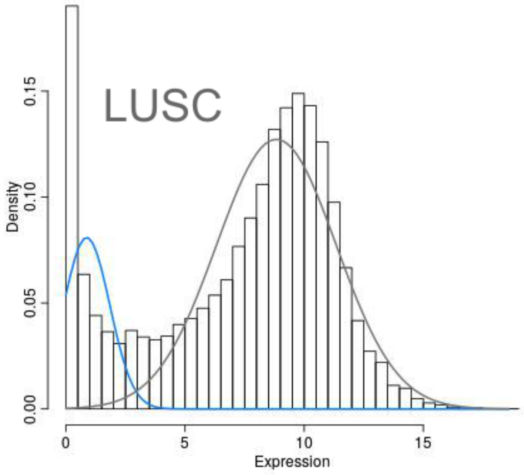

Figure D: Distribution of gene expression data in LUSC paired healthy samples

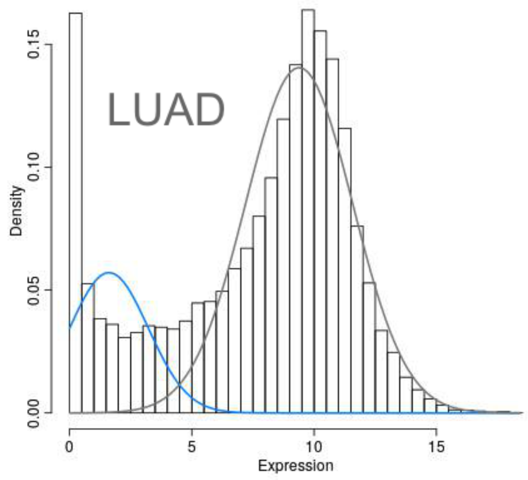

Figure E: Distribution of gene expression data in LUAD cancer samples

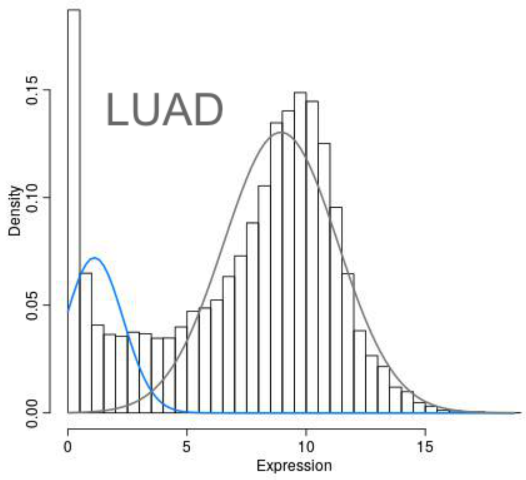

Figure E: Distribution of gene expression data in LUAD paired healthy samples

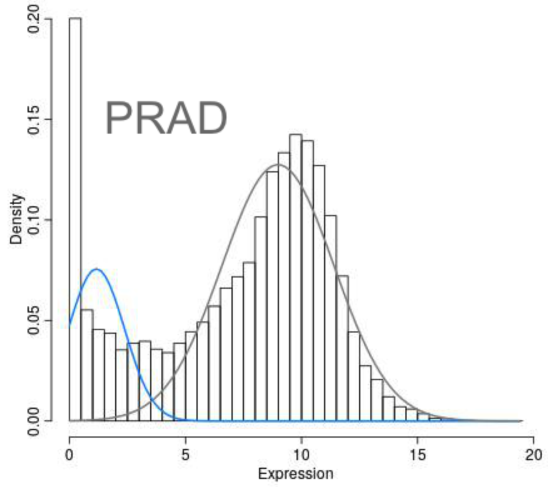

Figure F: Distribution of gene expression data in PRAD cancer samples

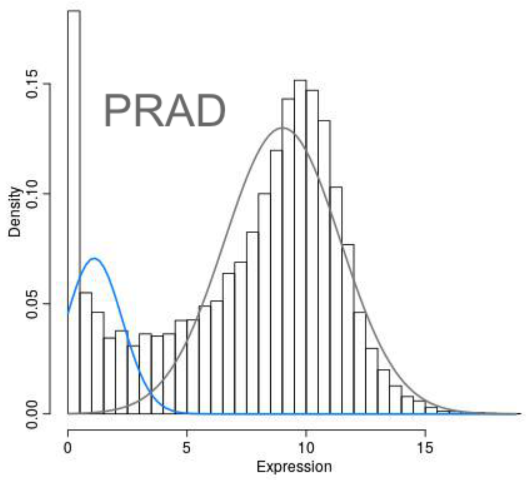

Figure F: Distribution of gene expression data in PRAD paired healthy samples

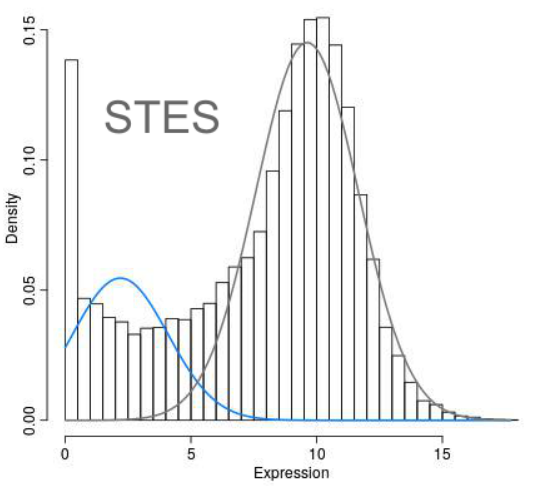

Figure G: Distribution of gene expression data in STES cancer samples

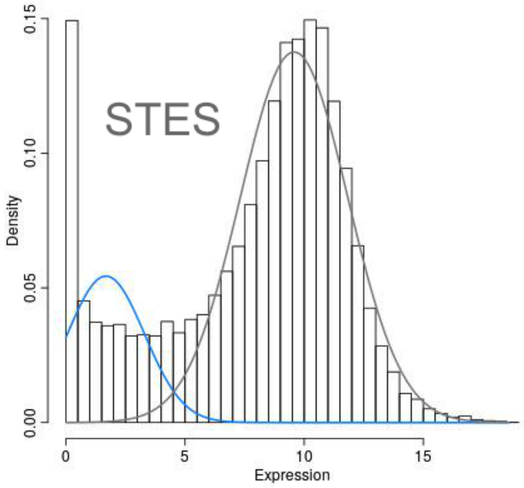

Figure G: Distribution of gene expression data in STES paired healthy samples

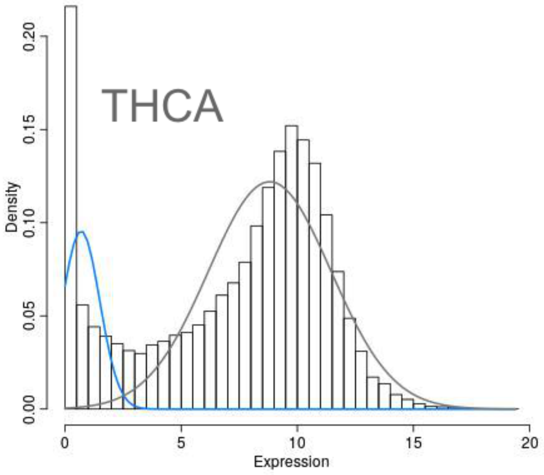

Figure H: Distribution of gene expression data in THCA cancer samples

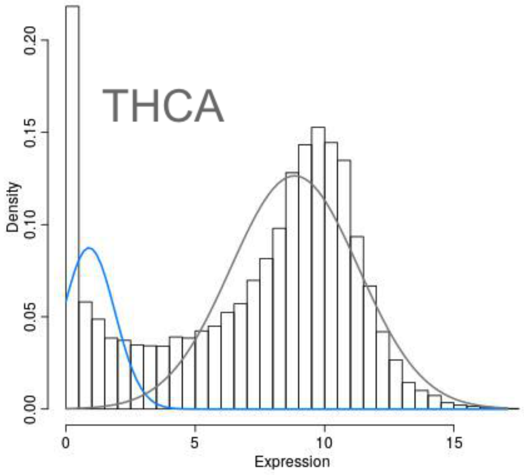

Figure H: Distribution of gene expression data in THCA paired healthy samples

S6 Fig (A-H): For each plot, the left blue curve represents the lowly-expressed genes while the grey curve represents the highly-expressed genes across patients of a cancer type for both healthy and cancer samples, respectively. We used these characteristic peaks as a threshold and only kept the genes with an all-samples probability score of greater than 0.8 for subsequent analysis.
